# Suppressive role exerted by microRNA-29b-1-5p in triple negative breast cancer through SPIN1 regulation

**DOI:** 10.18632/oncotarget.15960

**Published:** 2017-03-07

**Authors:** Rosa Drago-Ferrante, Francesca Pentimalli, Daniela Carlisi, Anna De Blasio, Christian Saliba, Shawn Baldacchino, James Degaetano, Joseph Debono, Gordon Caruana-Dingli, Godfrey Grech, Christian Scerri, Giovanni Tesoriere, Antonio Giordano, Renza Vento, Riccardo Di Fiore

**Affiliations:** ^1^ Laboratory of Biochemistry, Department of Biological, Chemical and Pharmaceutical Sciences and Technologies, University of Palermo, Polyclinic, Palermo, Italy; ^2^ Oncology Research Center of Mercogliano (CROM), Istituto Nazionale per lo Studio e la Cura dei Tumori “Fondazione Giovanni Pascale”, IRCCS, Naples, Italy; ^3^ Laboratory of Biochemistry, Department of Experimental Biomedicine and Clinical Neurosciences, University of Palermo, Polyclinic, Palermo, Italy; ^4^ Centre of Molecular Medicine and Biobanking, University of Malta, Msida, MSD, Malta; ^5^ Department of Pathology, Faculty of Medicine and Surgery, University of Malta, Msida, MSD, Malta; ^6^ Department of Pathology, Mater Dei Hospital, Msida, MSD, Malta; ^7^ Department of Surgery, Mater Dei Hospital, Msida, MSD, Malta; ^8^ Department of Physiology and Biochemistry, Faculty of Medicine and Surgery, University of Malta, Msida, MSD, Malta; ^9^ Sbarro Institute for Cancer Research and Molecular Medicine, Center for Biotechnology, College of Science and Technology, Temple University, Philadelphia, PA, USA; ^10^ Department of Medicine, Surgery & Neuroscience, University of Siena, Siena, Italy; ^11^ Associazione Siciliana per la Lotta contro i Tumori (ASLOT), Palermo, Italy

**Keywords:** MiR-29b-1, triple-negative breast cancer, cancer stem cells, SPIN1, Wnt/β-catenin and Akt signaling pathways

## Abstract

MiR-29 family dysregulation occurs in various cancers including breast cancers. We investigated miR-29b-1 functional role in human triple negative breast cancer (TNBC) the most aggressive breast cancer subtype. We found that miR-29b-1-5p was downregulated in human TNBC tissues and cell lines. To assess whether miR-29b-1-5p correlated with TNBC regenerative potential, we evaluated cancer stem cell enrichment in our TNBC cell lines, and found that only MDA-MB-231 and BT-20 produced primary, secondary and tertiary mammospheres, which were progressively enriched in *OCT4*, *NANOG* and *SOX2* stemness genes. MiR-29b-1-5p expression inversely correlated with mammosphere stemness potential, and miR-29b-1 ectopic overexpression decreased TNBC cell growth, self-renewal, migration, invasiveness and paclitaxel resistance repressing WNT/βcatenin and AKT signaling pathways and stemness regulators. We identified *SPINDLIN1* (*SPIN1*) among predicted miR-29b-1-5p targets. Consistently, *SPIN1* was overexpressed in most TNBC tissues and cell lines and negatively correlated with miR-29b-1-5p. Target site inhibition showed that *SPIN1* seems to be directly controlled by miR-29b-1-5p. Silencing *SPIN1* mirrored the effects triggered by miR-29b-1 overexpression, whereas *SPIN1* rescue by *SPIN1*miScript protector, determined the reversal of the molecular effects produced by the mimic-miR-29b-1-5p. Overall, we show that miR-29b-1 deregulation impacts on multiple oncogenic features of TNBC cells and their renewal potential, acting, at least partly, through SPIN1, and suggest that both these factors should be evaluated as new possible therapeutic targets against TNBC.

## INTRODUCTION

Despite improved diagnostic skills and breakthroughs in effective treatment, breast cancer continues to be the leading cause of cancer deaths among women worldwide, with breast cancer incidence and death rates generally increasing with age [1]. Human triple-negative breast cancer (TNBC), which accounts for approximately 10–20% of all breast tumors, refers to forms that do not express estrogen receptor (ER), progesterone receptor (PR) and human epidermal growth factor receptor 2 (HER2) [2]. These forms present a very difficult therapeutic challenge as are characterized by high heterogeneity, a particularly aggressive nature and by the lack of targeted therapies [3]. Although the metastatic potential in TNBC is similar to that of other breast cancer subtypes, these tumors exhibit the highest therapy resistance and the poorest prognosis, and are associated with a shorter median time to relapse and death [4]. Thus, achieving a better understanding of the molecular mechanisms underlying TNBC is a crucial need to identify novel diagnostic/prognostic biomarkers and to develop novel therapeutic strategies. In particular, it is recognized that, to be effective, therapeutic strategies need to eradicate cancer stem cells (CSCs), a subpopulation of cancer cells that are thought to be at the root of cancer [5]. Indeed, CSCs seem to be the cause of cancer initiation, growth and development, the source for tissue renewal and malignant potential, the crucial component leading to tumor recurrence, therapy resistance, and metastasis [6].

Recently it has been shown that in human cancers the malignant potential also depends on a widespread deregulation of microRNAs (miRNAs, miRs) [7], a class of small non-coding RNAs that regulate gene expression post-transcriptionally and may function as a novel class of oncogenes and tumor suppressor genes [8]. Several studies have shown that miRNAs are involved in the self-renewal and fate decisions of stem cells and that in CSCs these mechanisms are altered [9, 10]. Dysregulation of miRNAs has been reported in human cancers including breast cancer [11, 12]. Mir-29b is known to regulate a number of important genes that mediate carcinogenesis and tumor development in breast cancer [13]. Moreover, in breast cancer patients miR-29b is shown to act as tumor suppressor and low miR-29b expression in primary tumor tissues is a prognostic factor for breast cancer patients [14].

However, although miR-29b is considered to be a tumor suppressor in multiple types of cancers [15, 16], very few studies have investigated the effects of miR-29b-1-5p in human breast cancer cells and none in TNBC cells. This is a particularly important aspect of our research, because it is known that in the big family of miR-29 each member plays often opposing functions. In particular, it has been reported that in mouse breast cancer cells, miR-29b inhibits metastasis by targeting a network of pro-metastatic regulators involved in angiogenesis, collagen remodeling and proteolysis [13], whereas in the metastatic human breast cancer cell line MDA-MB-231, aberrant expression of miR-29b can contribute to migration and invasion [17]. Moreover, in many malignant cells miR-29b was proved to be an epi-miRNA that targets DNA methyltransferases (DNMTs) and/or regulates DNA demethylation pathway members, leading to the downregulation of global DNA methylation [18]. In addition, in human ER+PR+ breast cancer cells, the downregulation of miR-29 members increases mammosphere formation *in vitro* and tumor initiating capability *in vivo* [19].

In our previous studies on osteosarcoma (OS), a tumor in which the miRNA-29 family members (miR-29a/b/c; miR-29s) are often deregulated [20, 21], we found that ectopic expression of miR-29b-1 was able to suppress the stemness properties of the 3AB-OS cell line [22], a novel CSC line by us produced [23], suggesting that miR-29b-1 could be a novel therapeutic agent against OS. This background led us to study the role of miR-29b-1 in TNBC cells.

Here we demonstrated that miR-29b-1-5p expression was significantly downregulated in most TNBC tissues and in all the examined cell lines. MDA-MB-231 and BT-20 cells can produce primary, secondary and tertiary mammospheres which possess great regenerative properties and high levels of stemness genes (*OCT4*, *SOX2* and *NANOG*). In MDA-MB-231 and BT-20 cells, miR-29b-1 ectopic expression strongly decreased cell proliferation, viability, self-renewal, migration and invasiveness, also increasing sensitivity to paclitaxel. We found that *SPIN1* seems to be directly controlled by miR-29b-1-5p and that *SPIN1* silencing mirrored the effects of miR-29b-1 overexpression. Thus, in TNBC cells the simultaneous miR-29b-1-5p down regulation and *SPIN1* up-regulation can potentially be associated with TNBC malignancy and may be a potential new druggable target for TNBC.

## RESULTS

### MiRNA-29b-1-5p is downregulated in TNBC tissues and cell lines

**Figure 1:**
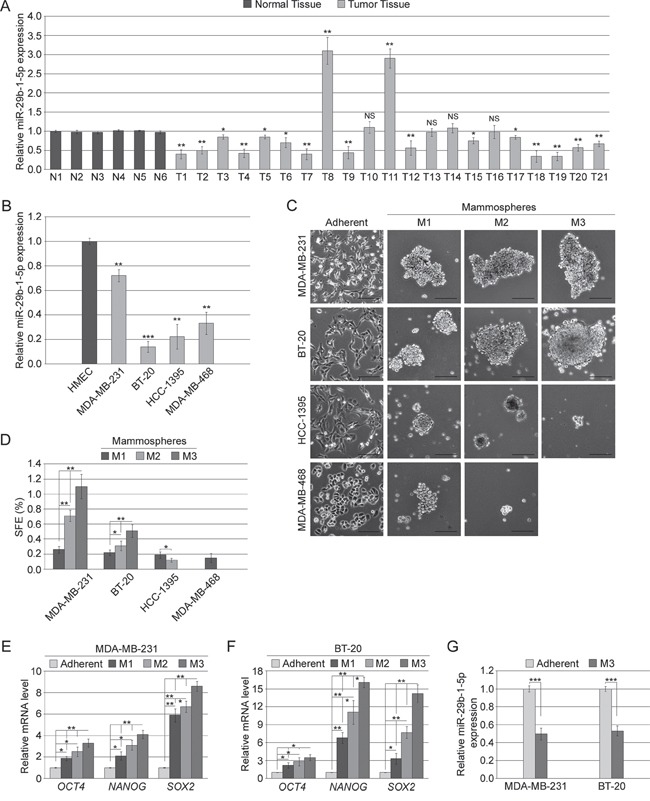
MiR-29b-1-5p expression in TNBC tissues and cell lines, and mammosphere formation ability of TNBC cell lines **(A)** miR-29b-1-5p expression was determined in 21 TNBC specimens (T) compared to 6 controls (N). **(B)** miR-29b-1-5p expression was determined in four human TNBC cell lines compared to normal human mammary epithelial cell line (HMEC). Data represent the mean with standard deviation (n = 3 independent experiments carried out in triplicate); NS, not significant; * P < 0.05; ** P < 0.01; *** P < 0.001. **(C)** Phase contrast microscopy of adherent cells and of primary (M1), secondary (M2) and tertiary (M3) mammospheres formed by TNBC cell lines. The scale bar represents 100 μm. **(D)** Bar graph represents the sphere forming efficiency (SFE) calculated for each passage as described in Materials and Methods. Data represent the mean with standard deviation (n = 3 independent experiments carried out in quadruplicate); * P < 0.05; ** P < 0.01. (**E** and **F**) Real-time RT-PCR analysis of stemness genes in M1, M2 and M3 mammospheres by MDA-MB-231 and BT-20 cells, respectively. Data represent the mean with standard deviation (n = 3 independent experiments carried out in triplicate); * P < 0.05; ** P < 0.01 as compared to adherent cells. **(G)** miR-29b-1-5p expression in tertiary mammospheres (M3) formed by both MDA-MB-231 and BT-20 cell lines compared to adherent cells. Data represent the mean with standard deviation (n = 3 independent experiments carried out in triplicate); ***, P < 0.001.

The expression of miR-29b-1-5p in human triple-negative breast cancer (TNBC) tissues and cell lines, was evaluated by quantitative RT-PCR (qRT-PCR). In TNBC tissues the analysis was carried out in 21 formalin-fixed, paraffin-embedded (FFPE) cancerous tissues, compared to 6 normal human mammary tissues; in TNBC cell lines the analysis was performed in MDA-MB-231, BT-20, HCC1395 and MDA-MB-468 cells compared to HMEC, a normal human mammary epithelial cell line. We found that miR-29b-1-5p expression was downregulated in fifteen of the twenty-one TNBC tissues (71.4%); a potent upregulation was observed in two of the twenty-one tissues (9.5%); no variations were observed in the other four TNBC tissues (Figure 1A). The analysis of miR-29b-1-5p expression in all the four TNBC cell lines studies evidenced its strong downregulation (Figure 1B). These findings suggested that miR-29b-1-5p down-regulation could play a role in TNBC development.

### MiRNA-29b-1-5p and TNBC stem cell features

To assess whether miR-29b-1-5p expression correlated with TNBC regenerative potential, we first evaluated the enrichment in CSCs of the TNBC cell lines. We evaluated their “mammosphere forming ability”, an assay which tests the ability to form organoid spheres in serum free medium in low adherences dishes, which is a recognized property of cells which contain CSCs [24] and have self-renewal potential [25]. In particular, the TNBC cell lines above described were tested for their ability to produce primary, secondary and tertiary mammospheres. Only MDA-MB-231 and BT-20 cell lines were capable of generating mammospheres until the tertiary stage, whereas the HCC-1395 cell line failed to generate tertiary spheres and MDA-MB-468 cell line were even incapable of generating secondary mammospheres, only producing some unstable aggregations (Figure 1C and 1D), thus suggesting that MDA-MB-231 and BT-20 cell lines possess a regenerative capacity greater than the other TNBC cell lines. Because the regenerative capacity depends on stemness properties, we also evaluated the relative expression of stemness genes at the three mammosphere stages. With respect to the adherent cells, from the primary to the tertiary mammosphere stages, in both, MDA-MB-231 cells and BT-20 cell we observed a progressive enrichment in the *OCT4*, *NANOG* and *SOX2* stemness genes (Figure 1E and 1F). Interestingly, in tertiary mammospheres compared to adherent cells, miR-29b-1-5p expression dramatically decreased (Figure 1G), suggesting its inverse correlation with stemness.

### Ectopic overexpression of miR-29b-1 in MDA-MB-231 cells and BT-20 cells

**Figure 2:**
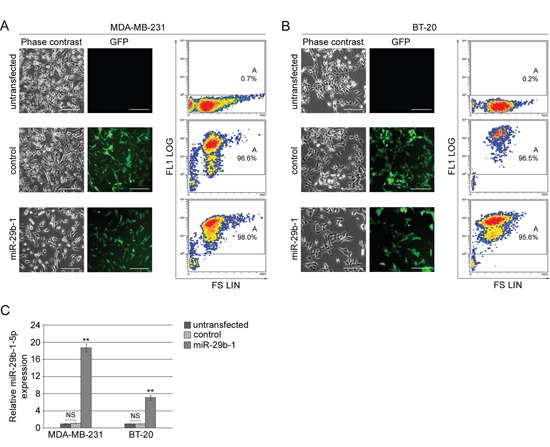
Ectopic expression of miR-29b-1 in MDA-MB-231 and BT-20 cell lines (**A** and **B**) Phase contrast and fluorescence microscopy of untransfected cells and cells stably transfected with either empty vector (control) or miR-29b-1 expressing vector (miR-29b-1). The scale bar represents 100 μm. In flow cytofluorimetric analysis the untransfected cells were used as negative control. The analysis shows the density plots of forward scatter (FS; linear scale) vs FL1 channel (FL1 log scale). Images are representative of four independent experiments. **(C)** Real-time RT-PCR analysis, in both cell lines, of miR-29b-1-5p in untransfected cells, cells stably transfected with either empty vector (control) or miR-29b-1 expressing vector (miR-29b-1). Data represent the mean with standard deviation (n = 4 independent experiments carried out in triplicate); NS, not significant; ** P < 0.01 as compared to untransfected cells.

To determine the role of miR-29b-1 in TNBC cells, MDA-MB-231 cells (Figure 2A) and BT-20 cells (Figure 2B) were stably transfected with either empty vector (control cells) or vector containing pre-miR-29b-1 (miR-29b-1 cells) and compared to untransfected cells. Phase contrast microscopy (left panels) fluorescence microscopy (middle panels) and flow cytometry (right panels) of the green fluorescent protein (GFP) show, in both control and miR-29b-1 cells, a strong positivity for GFP (>92%), which demonstrates a high transfection efficiency. QRT-PCR assays (Figure 2C) evidences that, following pre-miR-29b-1 transfection, miR-29b-1-5p levels were up-regulated in MDA-MB-231 and BT-20 cells, compared with both control and untransfected cells, by approximately 19-fold and 6.5-fold, respectively (Figure 2C).

### MiR-29b-1 inhibited MDA-MB-231 and BT-20 cell proliferation by perturbing cell cycle

**Figure 3:**
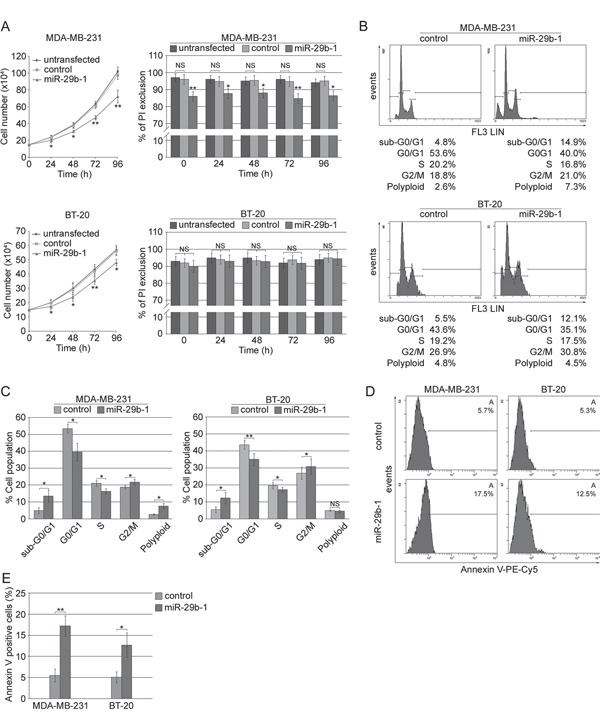
Effect of ectopic expression of miR-29b-1 on cell growth, viability, cell cycle distribution and apoptosis in MDA-MB-231 and BT-20 cell lines **(A)** Cellular growth (left panels) and viability (right panels) in both cell lines (untransfected and transfected cells) were evaluated by cell counting and PI exclusion method, respectively. Data represent the mean with standard deviation (n = 3 independent experiments carried out in triplicate); NS, not significant; * P < 0.05, ** P < 0.01 as compared to untransfected cells. **(B)** Histogram plots of flow cytometry analysis performed in both MDA-MB-231 (top panels) and BT-20 (bottom panels) transfected cells (control and miR-29b-1). **(C)** Cell cycle distribution determined by flow cytometry. Data represent the mean with standard deviation (n = 3 independent experiments carried out in triplicate); NS, not significant; * P < 0.05, ** P < 0.01 as compared to control cells. **(D)** Histogram plots of Annexin V flow cytometry analysis performed in both MDA-MB-231 (left panels) and BT-20 (right panels) transfected cells (control and miR-29b-1) are shown. Images are representative of four independent experiments. **(E)** Graph summarizing the percentage of Annexin V positive cells. Data represent the mean with standard deviation (n = 4 independent experiments carried out in triplicate); * P < 0.05, ** P < 0.01 as compared to control cells.

**Figure 4:**
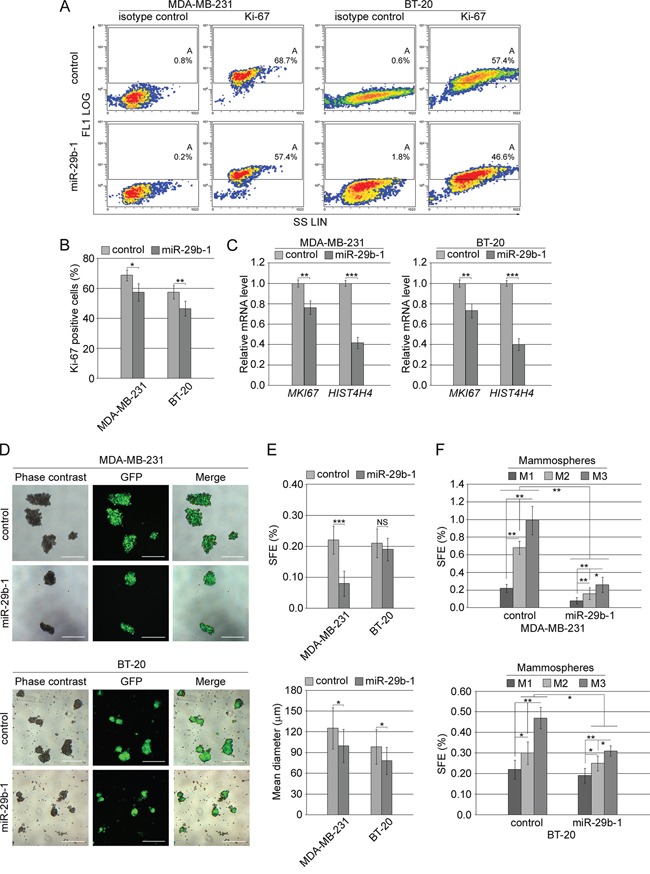
Effect of miR-29b-1 overexpression on cell proliferation markers and self-renewal in MDA-MB-231 and BT-20 cell lines **(A)** Flow cytofluorimetric analysis in both transfected cell lines, of Ki-67 positive cells in comparison with isotype control. Density plots of side scatter (SS; linear scale) vs FL1 channel (FL1 log scale) are shown. Images are representative of four independent experiments. **(B)** Graph summarizing Ki-67 reactivity. Data represent the mean with standard deviation (n = 4 independent experiments carried out in triplicate); * P < 0.05, ** P < 0.01 as compared to control cells. **(C)** Real-time RT-PCR analysis of cell proliferation markers in both the transfected cell lines. Data represent the mean with standard deviation (n = 3 independent experiments carried out in triplicate); ** P < 0.01 ***, P < 0.001 as compared to control cells. **(D)** Phase contrast and fluorescence microscopy and merge images of primary mammospheres from both the transfected cell lines after 10 days in culture. The scale bar represents 200 μm. Images are representative of four independent experiments. **(E)** Bar graphs represent the sphere forming efficiency (SFE) and size of mammospheres. Data represent the mean with standard deviation (n = 4 independent experiments carried out in triplicate); NS, not significant; * P < 0.05; ***, P < 0.001 as compared to control cells. **(F)** Bar graph represents the sphere forming efficiency (SFE) calculated for each passage [primary (M1), secondary (M2) and tertiary (M3) mammospheres]. Data represent the mean with standard deviation (n = 4 independent experiments carried out in triplicate); * P < 0.05; **, P < 0.01.

To investigate the effects of miR-29b-1 overexpression in MDA-MB-231 cells and BT-20 cells (Figure 3A), the cells were incubated for 0-96 h under cultural conditions, then, cell growth was analyzed by cell count, and cell viability by propidium iodide (PI) exclusion. MiR-29b-1 overexpression significantly decreased growth rate in both cell lines (left panels), showing decreased viability in MDA-MB-231 cells, but not in BT-20 cells (right panels). As during these studies we never observed statistically significant differences (P>0.05) between untransfected cells and cells transfected with the empty vector, in subsequent experiments, cells transfected with the empty vector were then used as controls. Cell cycle analysis by flow cytometry evidenced that in MDA-MB-231 cells compared to control cells, (Figure 3B, top panel and 3C, left panel) miR-29b-1 overexpression increased the percentage of cells at the sub-G0-G1 and G2-M-phases by 11.1% and 2.2% respectively, whereas it decreased the percentage of cells at the G0-G1 and S phases by 13.6% and 3.9% respectively. In BT-20 cells (Figure 3B, bottom panel and 3C, right panel) miR-29b-1 overexpression increased the percentage of cells at the sub-G0-G1 and G2-M-phases by 6.6% and 3.9% respectively, whereas at G0-G1 and S phases the percentage of cells decreased by 8.5% and 1.7%. Ectopic miR-29b-1 also increased the rate of polyploidy by 4.7% in MDA-MB-231 cells compared with control cells but not in BT-20 cells. Interestingly, the accumulation of the cells at the sub-G0/G1 phase and flow cytometry analysis of the Annexin V (Figure 3D and 3E) suggest that ectopic miR-29b-1 expression could lead to apoptosis. Cytofluorimetric analysis of the proliferation marker Ki-67 (Figure 4A and 4B) shows that by miR-29b-1 overexpression, both MDA-MB-231 and BT-20 cells resulted to be less Ki-67-positive than control cells. In addition, RT-PCR analyses of the proliferative markers *MKI67* and *HIST4H4*, show that miR-29b-1 markedly decreased their levels (Figure 4C).

### MiR-29b-1 overexpression markedly decreased self-renewal capacity of MDA-MB-231 and BT-20 cells

To test whether, low/absent miR-29b-1 levels are necessary for self-renewal in MDA-MB-231 and BT-20 cells, we studied the effect of miR-29b-1 overexpression by mammosphere assay. By this assay, the number of the formed mammospheres reflects the quantity of cells capable of *in vitro* self-renewal (Sphere forming efficiency, SFE), whereas the mammosphere size measures the self-renewal capacity of each sphere-generating cell [24]. Phase contrast and fluorescence microscopy (Figure 4D, top panel) show that MDA-MB-231 control cells efficiently formed spheres whereas, following miR-29b-1 overexpression, they formed fewer primary mammospheres than control cells. Also BT20 cells formed mammospheres (Figure 4D bottom panel), but they appeared smaller than these formed by MDA-MB-231-mammospheres and their culture medium contained a great number of cells probably released by some loosely packed mammospheres. The same figure also shows that miR-29b-1 decreased their dimension.

In MDA-MB-231 cells the percentage of SFE of miR-29b-1 cells was about 64% lower than control cells (Figure 4E, top panel, left), whereas the mean diameter of miR-29b-1 spheres (bottom panel, left)) was about 22% smaller than in control spheres. In BT20 cells (Figure 4E, top panel, right) the percentage of SFE of miR-29b-1 compared to control cells did not vary, whereas the mean diameter of miR-29b-1 spheres (bottom panel, right) was about 21% smaller than in control spheres. This suggested that in both cell lines the self-renewal capacity of each miR-29b-1 cell-generating sphere was much lower than in control cells.

To further assess whether miR-29b-1 may control mammosphere self-renewal, primary mammospheres were dissociated into single cells and reseeded to analyze the ability to form secondary and tertiary mammospheres. We found, in both, MDA-MB-231 and BT-20 cells (Figure 4F, top and bottom panels) that the inhibition of mammosphere-formation determined by miR-29b-1 overexpression was maintained also in the secondary and tertiary mammospheres. The evidence that at each mammosphere stage the relative self-renewal capacity increased, suggests a progressive enrichment in CSCs. Overall, these data reinforce the idea that miR-29b-1 may be involved in the control of growth and self-renewal capacity of MDA-MB-231 and BT-20 cells.

### MiR-29b-1 overexpression strongly inhibited migration and invasion of MDA-MB-231 and BT-20 cells

**Figure 5:**
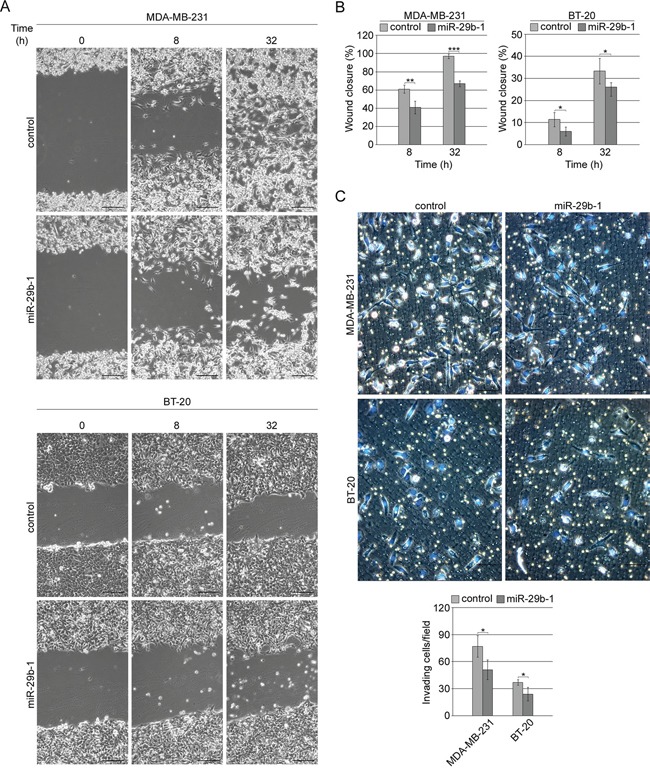
MiR-29b-1 overexpression reduces migration and invasion of MDA-MB-231 and BT-20 cell lines **(A)** Representative phase contrast microscopy from the wound-healing assay. The scale bar represents 100 μm. **(B)** Quantification of the scratch wound-healing assay. The extent of wound closure was quantified by measuring the wound area compared to the initial wound area. Data represent the mean with standard deviation (n = 4 independent experiments carried out in triplicate); * P < 0.05; ** P < 0.01 ***, P < 0.001 as compared to control cells. **(C)** Representative images from the transwell invasion assays. Invading cells were stained with Hoechst 33342 (merged images). The scale bar represents 50 μm. Bar graph represents the number of invading cells per field. Data represent the mean with standard deviation (n = 4 independent experiments carried out in triplicate); * P < 0.05 as compared to control cells.

In MDA-MB-231 cells wound healing assay (Figure 5A, top panel and 5B, left panel) showed that, at 8 h and 32 h after scratching, cells overexpressing miR-29b-1 migrated more slowly than control cells. Indeed, after 32 h, the wound area was almost recovered in control cells whereas in miR-29b-1 cells wound closure was 38% lower. Moreover, miR-29b-1 significantly decreased the invasive ability of MDA-MB-231 cells (Figure 5C, top panel), which was 34% lower than control cells. BT-20 cells (Figure 5A, bottom panel and 5B, right panel) evidenced a migration ability much lower than MDA-MB-231 cells, as at 32 h, the scratch in control cells was very far from being repaired and miR-29b-1 overexpression furthermore lowered (about 10%) cell repairing capacity. Also the relative invasion ability of both MDA-MB-231 and BT-20 was severely impaired by miR-29b-1 overexpression, as assessed through transwell invasion assay (Figure 5C, bottom panels).

### In MDA-MB-231 and BT-20 cells paclitaxel enhances the perturbing effects induced on cell cycle by miR-29b-1 overexpression

**Figure 6:**
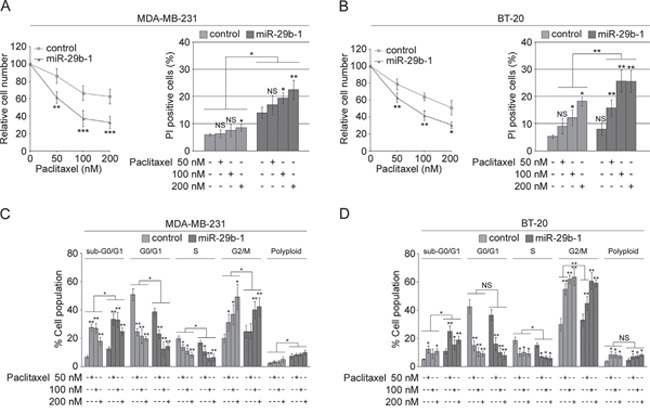
MiR-29b-1 overexpression increases sensitivity of MDA-MB-231 and BT-20 cell lines to paclitaxel (**A** and **B**) Both the transfected cell lines were treated with paclitaxel (50, 100 and 200 nM) for 24 h. Relative cell number was determined by counting the cells and dividing the number of live cells arising from each treatment condition, with respect to the untreated control, multiplied hundred. Dead cells were evaluated using PI exclusion assay. Data represent the mean with standard deviation (n = 4 independent experiments carried out in triplicate); NS, not significant; * P < 0.05; ** P < 0.01 ***, P < 0.001. (**C** and **D**) Cell cycle distribution of both the transfected cell lines using flow cytometry. Data represent the mean with standard deviation (n = 4 independent experiments carried out in triplicate); NS, not significant; * P < 0.05 and ** P < 0.01.

To assess whether miR-29b-1 affects cell susceptibility to paclitaxel (one of the major drugs used for breast cancer chemotherapy), we treated MDA-MB-231 and BT-20 miR-29b-1overexpressing cells and their relative controls with 50, 100 and 200 nM paclitaxel for 24h. In MDA-MB-231 cells (Figure 6A) analysis of relative cell number (left panel) shows that, in control cells, 50, 100 and 200 nM paclitaxel decrease cell growth by 13.7%, 33.4% and 37.3% respectively, whereas in cells overexpressing miR-29b-1 the decrease was 35.4%, 62.6% and 67%, respectively. Analysis of cell viability by PI exclusion (right panel) shows that, in control cells the effect of paclitaxel was significant only at 200 nM which increased the percentage of PI positive cells from 6% to 8.5%. MiR-29b-1 overexpression increased to 14% the percentage of PI positive cells, whereas 100 and 200 nM paclitaxel increased this percentage to 19.4% and 22.5% respectively. In BT-20 cells (Figure 6B), 50, 100 and 200 nM paclitaxel decreased the growth of control cells by 21.3%, 36.1% and 49.1% respectively, whereas in miR-29b-1 cells these concentrations decreased the growth by 37.2%, 58.3% and 69.3% respectively (left panel). PI exclusion (right panel) shows that, in control cells 100 and 200 nM paclitaxel significantly increased the percentage of PI positive cells from 5.3% to 12.2% and 18.3%, respectively. In BT-20 cells overexpressing miR-29b-1 paclitaxel 50 nM and 100 nM increased the percentage of PI positive cells from 7.9% to 15.8% and 25.6%, respectively. No further increases in cytotoxicity were observed with 200 nm paclitaxel. We also assessed whether paclitaxel induces changes on the perturbing activity of miR-29b-1 overexpression on cell cycle. Flow cytometry assay of DNA content shows that, in both MDA-MB-231 and BT-20 cells (Figure 6C and 6D) paclitaxel markedly increased the percentage of cells accumulated in the sub-G0/G1 and G2-M phases of cell cycle, and decreased those in G0/G1 and S phases. Overall, these results suggest that ectopic miR-29b-1 expression sensitizes the cells to the effects of paclitaxel. Interestingly, the effects of paclitaxel were in keeping with the perturbing effects of miR-29b-1 on the cell cycle.

### SPIN1 is under the control of miR-29b-1-5p

**Figure 7:**
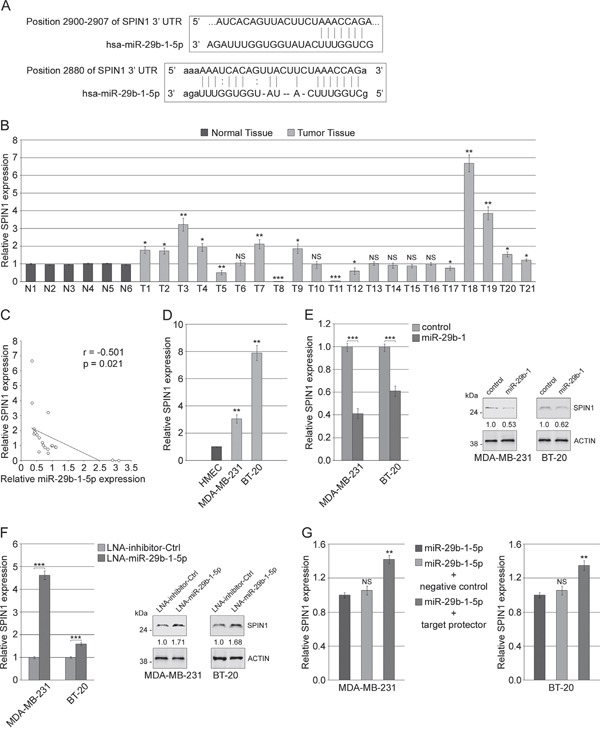
MiR-29b-1-5p targets SPIN1 **(A)** MiR-29b-1-5p predicted target site within SPIN1 3′UTR identified by TargetScan (top) and microRNA (bottom) online software. **(B)** SPIN1 expression was determined by real-time RT-PCR in 21 TNBC specimens (T) compared to 6 controls (N). Data represent the mean with standard deviation (n = 3 independent experiments carried out in triplicate); NS, not significant; * P < 0.05; ** P < 0.01; *** P < 0.001. **(C)** The correlation between miR-29b-1-5p and *SPIN1* mRNA expression in 21 TNBC tissues was evaluated using Pearson's correlation analysis. **(D)** SPIN1 expression in MDA-MB-231 and BT-20 cell lines compared to normal human mammary epithelial cell line (HMEC). Data represent the mean with standard deviation (n = 3 independent experiments carried out in triplicate); ** P < 0.01. **(E)** MDA-MB-231 and BT-20 cells stably transfected with either empty vector (control) or miR-29b-1 expressing vector (miR-29b-1). SPIN1 mRNA and protein expression were determined by Real-time RT-PCR and Western blot analyses, respectively. The numbers below the blot panels indicate the fold change of SPIN1 levels with respect to control. Actin was used as loading control. Data represent the mean with standard deviation (n = 4 independent experiments carried out in triplicate); *** P < 0.001 as compared to control cells. **(F)** MDA-MB-231 and BT-20 cells transfected with either LNA-inhibitor-Ctrl or LNA-miR-29b-1-5p. SPIN1 mRNA and protein expression were determined by Real-time RT-PCR and Western blot analyses, respectively. The numbers below the blot panels indicate the fold change of SPIN1 levels with respect to LNA-inhibitor-Ctrl. Actin was used as loading control. Data represent the mean with standard deviation (n = 4 independent experiments carried out in triplicate); *** P < 0.001 as compared to LNA-inhibitor-Ctrl cells. **(G)** MDA-MB-231 and BT-20 cells transfected with miR-29b-1-5p mimic alone, or co-transfected with miR-29b-1-5p mimic and negative control target protector or co-transfected with miR-29b-1-5p mimic and miScript target protector. *SPIN1* mRNA expression was determined by Real-time RT-PCR after 72 h of transfection. Data represent the mean with standard deviation (n = 4 independent experiments carried out in triplicate); NS, not significant; ** P < 0.01 as compared to miR-29b-1-5p mimic cells.

To identify potential miR-29b-1-5p targets, we used a bioinformatics approach through the MIRDB.org database. The MIRDB algorithm predicted a number of candidate target genes, listed in the Supplementary Table 1, among which we focused on *SPINDLIN1* (*SPIN1*), a histone code reader highly expressed in several types of tumors and strongly implicated in tumorigenesis and tumor growth [26]. Then, we chose to analyze, between the two putative miR binding sites on *SPIN1* 3′UTR identified by Targetscan 7.0 and microRNA.org., the one with the better score (Figure 7A). Eventually, we assessed whether *SPIN1* could be involved in MDA-MB-231 and BT-20 cells tumorigenesis and invasivity and if it could be a miR-29b-1-5p target. Firstly, we evaluated the expression of *SPIN1* in the human TNBC tissues and cell lines described above and characterized by miR-29b-1-5p down regulation. QRT-PCR analysis showed that, in comparison with normal breast tissues, *SPIN1* appears significantly up-regulated in 10 (47.6%) and down-regulated in 5 (23.8%) of the 21 TNBC specimens studied (Figure 7B) and the analysis of its correlation with miR-29b-1-5p overexpression demonstrated that it is negatively correlated with *SPIN1* expression (Figure 7C). We also showed that, in MDA-MB-231 and BT-20 cells compared to the normal human mammary epithelial cell line HMEC, *SPIN1* was strongly upregulated (Figure 7D). Moreover, qRT-PCR and western blot analyses showed that miR-29b-1-5p overexpression strongly impairs SPIN1 mRNA and protein levels (Figure 7E). Accordingly, in both cell lines the inhibition of endogenous miR-29b-1-5p by miR-29b-1-5p-LNA (a miR-29b-1-5p inhibitor), compared to the negative control, resulted in the upregulation of SPIN1 mRNA and protein (Figure 7F). These data suggest that miR-29b-1-5p can regulate *SPIN1* at both mRNA and protein levels. Moreover, after transfection with either hsa-miR-29b-1-5p mimic, or hsa-miR-29b-1-5p mimic plus negative control miScript target protector, or hsa-miR-29b-1-5p mimic plus miScript target protector (Figure 7G), the levels of *SPIN1* mRNA were strongly higher in cells transfected with hsa-miR-29b-1-5p mimic plus miScript target protector than in control cells (cells transfected with hsa-miR-29b-1-5p Mimic or hsa-miR-29b-1-5p Mimic plus Negative control miScript Target Protector). These results suggest that SPIN1 may be directly controlled by miR-29b-1-5p.

### In MDA-MB-231 cells, silencing SPIN1 mirrored the miR-29b-1 overexpression

**Figure 8:**
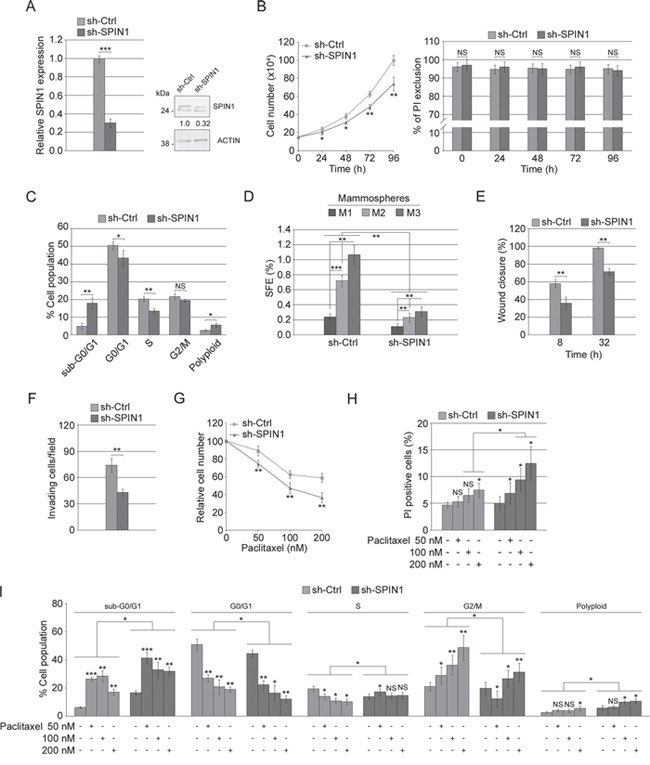
Effect of *SPIN1* silencing in MDA-MB-231 cells **(A)** Real-time RT-PCR and western blot analyses of MDA-MB-231 cells stably transfected with either sh-Ctrl or sh-SPIN1. The numbers below the blot panels indicate the fold change of SPIN1 levels with respect to sh-Ctrl. Actin was used as loading control. Data represent the mean with standard deviation (n = 4 independent experiments carried out in triplicate); NS, not significant; *** P < 0.001 as compared to sh-Ctrl cells. **(B)** Cellular growth and viability in transfected cells (sh-Ctrl or sh-SPIN1). Data represent the mean with standard deviation (n = 4 independent experiments carried out in triplicate); NS, not significant; * P < 0.05, ** P < 0.01 as compared to sh-Ctrl cells. **(C)** Histograms reporting cell cycle distribution assessed by flow cytometry. Data represent the mean with standard deviation (n = 4 independent experiments carried out in triplicate); NS, not significant; * P < 0.05, ** P < 0.01 as compared to sh-Ctrl cells. **(D)** Bar graph represents the sphere forming efficiency (SFE) calculated by counting for each passage [primary (M1), secondary (M2) and tertiary (M3) mammospheres]. Data represent the mean with standard deviation (n = 4 independent experiments carried out in triplicate); ** P < 0.01; ***, P < 0.001. **(E)** Quantification of the scratch wound-healing assay. The extent of wound closure was quantified by measuring the wound area compared with the initial wound area. Data represent the mean with standard deviation (n = 4 independent experiments carried out in triplicate); ** P < 0.01 as compared to sh-Ctrl cells. **(F)** Bar graph of the invasion assay. Bars represents the mean number of invading cells per field. Data represent the mean with standard deviation (n = 4 independent experiments carried out in triplicate); ** P < 0.01 as compared to sh-Ctrl cells. (**G, H** and **I**) Cell growth, cell viability and cell cycle distribution of transfected cells treated with paclitaxel (50, 100 and 200 nM) for 24 h. Data represent the mean with standard deviation (n = 4 independent experiments carried out in triplicate); NS, not significant; * P < 0.05; ** P < 0.01 and *** P < 0.001.

To explore the function of SPIN1 in TNBC cells, MDA-MB-231 cells (the most aggressive cell line within our panel) were stably transfected with either sh-SPIN1 or sh-Ctrl. QRT-PCR and western blot analyses show that, compared to sh-Ctrl cells, in sh-SPIN1 cells, SPIN1 levels profoundly lowered (Figure 8A). Then, using sh-SPIN1 cells compared to sh-Ctrl cells, we assessed cell proliferation, mammosphere-forming ability, migration/invasive ability and paclitaxel sensitivity. The results showed that (Figure 8B) sh-SPIN1 cells have a proliferative rate lower than sh-Ctrl cells (left panel), whereas their viability appeared unmodified (right panel). In addition, silencing *SPIN1* led to a strong perturbation of the cell cycle with an increase of cell percentage in subG0-G1 phase, a significant decrease in G0-G1 and S phases and an increase in polyploidy rate (Figure 8C). These results were very similar to those already reported in Figure 3B (top panel) and 3C (left panel) for MDA-MB-231 cells overexpressing miR-29b-1. Also for mammosphere formation, wound closure, cell invasivity and paclitaxel effects *SPIN1* silencing mimicked the effects of miR-29b-1 overexpression. Indeed, *SPIN1* silencing strongly inhibited the ability to form primary, secondary and tertiary mammospheres (Figure 8D), thus suggesting that *SPIN1* is involved in the control of self-renewal capacity of these cells. Moreover, sh-SPIN1 cells migrated more slowly (Figure 8E) and were less invasive (Figure 8F) than sh-Ctrl cells. Finally, sh-SPIN1 MDA-MB-231 cells were more responsive to paclitaxel treatment as shown by cell growth (Figure 8G), cell viability (Figure 8H) and cell cycle (Figure 8I) analysis.

Together, these results suggest that *SPIN1* silencing induces a phenotype similar to that produced by miR-29b-1 overexpression also proposing that *SPIN1* may be, at least in part, responsible for the effects triggered by miR-29b-1.

### In MDA-MB-231 and BT-20 cells miR-29b-1 overexpression represses WNT/β-catenin and Akt signalling pathways through *SPIN1*

Wnt/β-catenin and AKT signaling have been found to be aberrantly activated and to play crucial roles in the development and progression of breast cancer [27]. To evaluate the effects of miR-29b-1 overexpression on these pathways and the possible role of *SPIN1*, we employed MDA-MB-231 and BT-20 TNBC cells in which either miR-29b-1 was up/down-regulated or *SPIN1* expression was silenced.

**Figure 9:**
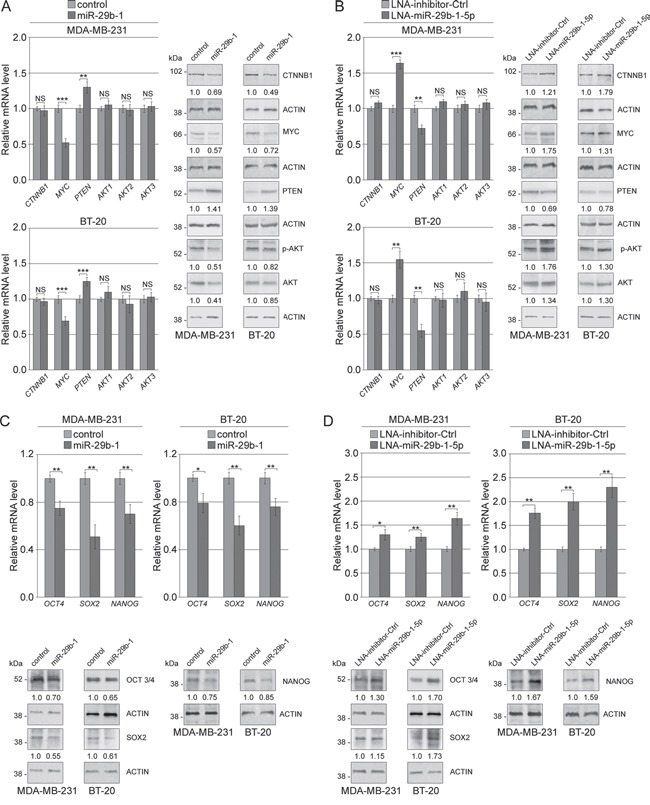
MiR-29b-1 represses Wnt/β-catenin and Akt signalling pathways through *SPIN1* in TNBC cells (**A** and **B**) Evaluation of mRNA and protein expression of Wnt/β-catenin, Akt pathway and stemness regulators in MDA-MB-231 and BT-20 cells stably transfected with empty vector (control) and miR-29b-1 expressing vector (miR-29b-1). Data represent the mean with standard deviation (n = 4 independent experiments carried out in triplicate); NS, not significant; * P < 0.05; ** P < 0.01 and *** P < 0.001 as compared to control cells. The numbers below the blot panels indicate the fold change of the signaling component levels with respect to control. Actin was used as loading control. (**C** and **D**) Evaluation of mRNA and protein expression of Wnt/β-catenin, Akt pathway and stemness regulators in MDA-MB-231 and BT-20 cells transfected with LNA-inhibitor-Ctrl and LNA-miR-29b-1-5p. Data represent the mean with standard deviation (n = 4 independent experiments carried out in triplicate); NS, not significant; * P < 0.05; ** P < 0.01 and *** P < 0.001 as compared to LNA-inhibitor-Ctrl cells. The numbers below the blot panels indicate the fold change of the signaling component levels with respect to LNA-inhibitor-Ctrl. Actin was used as loading control.

**Figure 10:**
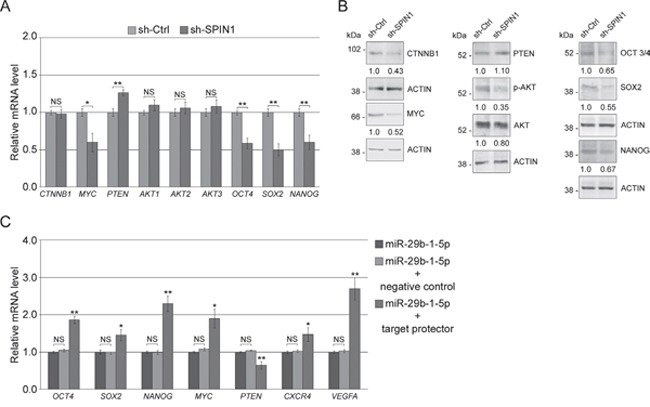
*SPIN1* silencing represses WNT/β-catenin, Akt signaling pathways and stemness regulators in MDA-MB-231 cells **(A)** MDA-MB-231 cells stably transfected with either sh-Ctrl or sh-SPIN1. Real-time RT-PCR analysis of Wnt/β-catenin, Akt pathway components and stemness genes. Data represent the mean with standard deviation (n = 4 independent experiments carried out in triplicate); NS, not significant; * P < 0.05 and ** P < 0.01 as compared to sh-Ctrl. **(B)** Western blot analysis of Wnt/β-catenin and Akt pathway components and stemness proteins. The numbers below the blot panels indicate the fold change of the signaling component levels with respect to sh-Ctrl. Actin was used as loading control. **(C)** MDA-MB-231 cells transfected with miR-29b-1-5p mimic alone, or co-transfected with miR-29b-1-5p mimic and negative control target protector or co-transfected with miR-29b-1-5p mimic and miScript target protector. mRNAs expression was determined by Real-time RT-PCR after 72 h of transfection. Data represent the mean with standard deviation (n = 4 independent experiments carried out in triplicate); NS, not significant; * P < 0.05 and ** P < 0.01 as compared to miR-29b-1-5p mimic cells.

QRT-PCR analysis (Figure 9A, left panel) showed that miR-29b-1 overexpression, compared to control cells, markedly decreased *MYC* levels whereas significantly increased *PTEN* levels. However miR-29b-1 did not change *AKT1,2,3* and β-catenin (*CTNNB1*) levels. Western blot analysis (Figure 9A, right panel) confirmed the effects of miR-29b-1 on MYC and PTEN expression, whereas, in contrast to mRNA analysis, it evidenced lower β-catenin and AKT1,2,3 levels. Also pAKT apparently lowered, but this probably depended on the AKT decrease. Since in several types of tumors, including breast cancer Wnt/β-catenin and Akt- signaling pathways are implicated in the regulation of self-renewal of CSCs, and recently Akt has been identified as an upstream regulator of SOX2 protein in breast carcinoma [28], we also investigated whether miR-29b-1 affected the expression of *OCT4*, *SOX2* and *NANOG*, key stemness genes. QRT-PCR and western blot analyses (Figure 9C) show that miR-29b-1 overexpression potently decreased the mRNA and protein levels of these genes (in particular SOX2). Overall, these results suggest that ectopic miR-29b-1 represses WNT/β-catenin and Akt signaling pathways. Thus, to assess if miR-29b-1 functions are required for the control of WNT/β-catenin and Akt signaling, we inhibited the endogenous miR-29b-1-5p by the miR-29b-1-5p-LNA inhibitor. The results show (Figure 9B and 9D) that this inhibitor determined opposite effects to those obtained by miR-29b-1 overexpression (Figure 9A and 9C). We also evaluated the effect of *SPIN1*-silencing on WNT/β-catenin and Akt signaling. QRT/PCR and western blot analyses (Figure 10A and 10B) evidenced effects very similar to those determined by miR-29b-1 overexpression. The only difference was that, in confront to that evidenced by miR-29b-1 overexpression (Figure 9A) here western blot analysis evidences a lowering in pAKT levels stronger than in AKT levels, suggesting that SPIN1 could even control AKT phosphorylation. To further elucidate the role of *SPIN1*, we also analyzed whether, using *SPIN1* miScript protector, the effects produced by the mimic-miR-29b-1-5p could be reversed. The results reported in Figure 10C demonstrate that *SPIN1* rescue determines the reversal of the molecular effects produced by the mimic-miR-29b-1-5p on the expression of genes (*OCT4, SOX2, NANOG, MYC, PTEN, CXCR4* and *VEGFA*) involved in the cell functions here analyzed.

Taken together, these data suggest that miR-29b-1-5p by downregulating *SPIN1* inhibits both WNT and Akt signaling pathways. This could contribute to the effects induced by miR-29b-1 on proliferation, self-renewal, migration, invasion and chemosensitivity in TNBC cells.

## DISCUSSION

TNBC, is the most aggressive breast cancer subtype with a high propensity for metastasis and a poor prognosis [2]. Moreover, as the molecular profile of this cancer makes it unresponsive to hormonal treatment, it lacks a targeted therapy and the patients are more susceptible to relapse thus aggravating the prognosis [29, 30]. Although many effective options have been developed for treating breast cancer, unfortunately TNBC remains a subtype that is difficult to treat. Therefore, the search for new prognostic/diagnostic biomarkers and therapeutic targets is an urgent and imperative occurrence.

MiRNAs are small non-coding endogenous RNAs (around 22–25 nucleotides in length), involved in the regulation of gene expression [31, 32]. Since their discovery, a plethora of miRNAs have been identified to be involved in the gene silencing in cancer. A lot of research has shed light on miRNA functions showing that they can act as either oncogenes or tumor suppressor genes in breast cancer and more specifically in TNBC [33, 34, 35], even suggesting that they could be therapeutic targets [36]. However, although various mechanisms underlying both miRNA biogenesis and normal or deregulated expression have been extensively described [37], many causes leading to their abnormal expression in cancer remain largely ununderstood and are becoming an emerging theme in cancer research.

It has been demonstrated that the miR-29 family might function as a tumor suppressor and that expression of these miRNAs inhibits cell proliferation, promotes apoptosis of cancer cells, and suppresses tumorigenicity by targeting multiple oncogenes [38, 39]. Loss or downregulation of these miRNAs have been correlated with a higher-risk to develop several cancers including chronic lymphocytic leukemia, lung cancer, invasive breast cancer, cholangiocarcinoma [11].

MiR-29 family consists of four closely related members (miR-29a, miR-29b-1, miR-29b-2 and miR-29c). However, albeit the miR-29 family members share a common seed region sequence (thus predicting that they target overlapping sets of genes), they frequently exhibit differential regulation and different subcellular distribution, suggesting different functional importance [40]. Dysregulation of miR-29 family has been reported in various cancers including breast cancers [15, 16, 34, 41, 42]. MiR-29b has been reported to have many targets and to regulate ECM protein expression and tumor microenvironment. Enrichment of miR-29b has been found in luminal breast cancers where it inhibits metastasis by targeting a network of pro-metastatic regulators involved in angiogenesis, collagen remodeling and proteolysis as VEGFA, PDGF, MMP9, indirectly affecting differentiation and epithelial plasticity, with loss of miR-29b increasing metastasis and promoting a mesenchymal phenotype. Moreover, in human luminal breast cancer, GATA3 - which works by regulating miRNA-29b expression - emerged as a strong predictor of clinical outcome [13].

Anyhow, to our knowledge, to date, the functional expression and the role of miR-29b-1-5p in breast cancer, particularly in TNBC cancer, have not yet been elucidated.

Here we evaluated miR-29b-1-5p expression in human TNBC specimens and cell lines, employing both human TNBC FFPE tissues and TNBC cell lines. We have found that miR-29b-1-5p expression -compared to normal conditions- was significantly down-regulated. This solidly suggested that miR-29b-1-5p downregulation could play a role in TNBC development.

It is well known that cancers are diseases driven by CSCs that are responsible for poor clinical outcome, because they are the cause of reduced sensitivity to therapies, tumor regrowth, metastatic spread and morbidity and mortality of patients. Indeed, most tumors recur after an apparently successful eradication, probably since CSCs survive and restore tumor growth [43]. Here, we evaluated the enrichment in CSCs of the TNBC cell lines, by their ability to produce primary, secondary and tertiary mammospheres. We demonstrated that MDA-MB-231 and BT-20 cells possess a regenerative capacity greater than other TNBC cells within our panel as they were the only capable of generating mammospheres until the tertiary stage. We also evaluated in them the expression of *OCT4*, *NANOG* and *SOX2* -stemness genes which can control the regenerative capacity of the cells [44]- showing that, compared to the adherent cells, from the primary to the tertiary mammospheres stages, there was a progressive enrichment in the expression of these genes. Similarly to the tumors from which derive, TNBC cells are characterized by a strong heterogeneity at the genetic and molecular levels, which affects their phenotypes such as aggressiveness, metastatic ability and response to drugs, [45]. Such high rate of heterogeneity is likely to underlie the different mammosphere-forming ability observed in our cell lines.

Interestingly, in tertiary mammospheres we also observed a sharp decrease of miR-29b-1-5p expression with an inverse correlation with stemness. Thus, to understand the role of miR-29b-1 in TNBC cells, it was ectopically overexpressed in MDA-MB-231 and BT-20 cells. We demonstrated that this overexpression potently decreased cell growth and viability, and strongly perturbed cell cycle. Moreover, ectopic miR-29b-1 markedly decreased self-renewal capacity of the cells even inhibiting their migration and invasive properties.

Aberrant expression of miRNAs has been associated with cancer chemoresistance, including resistance to paclitaxel [46] an anticancer drug belonging to the class of taxanes, which is a front-line chemotherapeutic agent for treating breast cancer [47]. Recently, it has been observed that in breast cancer cells miR-16 overexpression promoted Taxol-induced cytotoxicity and it has been suggested that miR-16 overexpression could be a strategy to overcome Taxol resistance in breast cancer [48].

In this study we analyzed the effects of paclitaxel on MDA-MB-231 and BT-20 cells in which miR-29b-1 was either downregulated or was ectopically overexpressed. We observed that, after miR-29b-1 overexpression, paclitaxel effects were more potent than in cells in which miR-29b-1 was downregulated. Moreover, the effects of paclitaxel on cell cycle were in keeping with the perturbing effects induced by miR-29b-1 overexpression.

Searching putative miR-29b-1-5p targets we have found an algorithm predicting *SPIN1* as potential target gene. It is known that SPIN1 is a histone code reader highly expressed in several types of cancers and strongly implicated in tumorigenesis and tumor growth [26, 49]. Its expression is upregulated in clinical tumor specimens and its ectopic expression promotes cancer cell proliferation through activation of WNT/β-catenin signaling [49]. We have shown that, in most TNBC specimens studied, *SPIN1* appeared significantly upregulated and negatively correlated with miR-29b-1-5p expression. We also found a strong *SPIN1* expression in both MDA-MB-231 and BT-20 cells, which was remarkably reduced following ectopic miR-29b-1-5p overexpression. Conversely, inhibiting endogenous miR-29b-1-5p, resulted in the upregulation of SPIN1 mRNA and protein, according to the fact that miR-29b-1-5p and *SPIN1* show an inverse correlation. In addition, using miScript target protector for *SPIN1*, our results suggested that *SPIN1* may be directly controlled by miR-29b-1-5p. Moreover, in MDA-MB-231 cells, cell growth, viability, self-renewal, responsiveness to paclitaxel, migration and invasion, similarly changed by either miR-29b-1 overexpression or *SPIN1*-silencing. Overall, these results suggest that miR-29b-1-5p could directly control *SPIN1* and that it could exert its effects by keeping *SPIN1* downregulated.

As Wnt/β-catenin and AKT signalling have been found to be aberrantly activated and to play crucial roles in the development and progression of breast cancer [27, 50, 51], we also analysed the effects of miR-29b-1 overexpression and the possible role of *SPIN1* on these pathways. We have found that, upon miR-29b-1 upregulation, the Wnt and Akt signaling pathways deeply fell in MDA-MB-231 and BT-20 cells. Moreover, miR-29b-1 overexpression markedly decreased *MYC* levels also lowering the expression of the key stemness genes *OCT4*, *SOX2* and *NANOG*. Conversely, the inhibition of miR-29b-1-5p produced opposite effects. It is interesting to note that in TNBC patients MYC expression has been found disproportionally elevated [52]. In addition, it has been reported that miR-29b-1/mir-29a promoter sequence is regulated by MYC, contributing to mir-29 downregulation in human malignancies [53]. We also showed that *SPIN1*-silencing decreased both the expression of the key stemness genes and MYC levels, thus mirroring the effects determined by miR-29b-1 overexpression. Moreover, using *SPIN1* miScript protector, we demonstrated that *SPIN1* rescue reversed the molecular effects produced by the mimic-miR-29b-1-5p on the expression of genes (*OCT4, SOX2, NANOG, MYC, PTEN, CXCR4* and *VEGFA*) involved in the cell functions analysed. These results are particularly intriguing as it is known that in samples of breast cancer patients *VEGF* mRNA levels are correlated with *CXCR4* mRNA levels, and that the CXCR4/CXCL12 signaling axis can induce angiogenesis and tumor progression by increasing expression of VEGF through the activation of PI3K/Akt pathway [54]. In conclusion, the results show that miR-29b-1-5p inhibits both WNT and Akt signaling pathways by downregulating *SPIN1* and suggest that miR-29b-1-5p, through *SPIN1*, might regulate the expression of *MYC*, *CXCR4* and *VEGFA* counteracting their contribution to tumorigenesis.

Overall, our data show that miR-29b-1-5p deregulation impacts on multiple oncogenic features of TNBC cells and their renewal potential, acting through SPIN1 action and suggest that both these factors should be further evaluated as new possible therapeutic targets against TNBC.

## MATERIALS AND METHODS

### Tissue samples

Twenty-six breast tissue samples were collected in this study, including six normal breast tissues as control and twenty-one specimens diagnosed as TNBC (Supplementary Table 2). The specimens were obtained during surgery and were formalin fixed and embedded in paraffin (FFPE) using standard methods. Apart from T16 that was a recurrence case, none of the patients included in the present study had received chemotherapy or radiation therapy prior to the study, and their complete clinical data were available, including age, histologic type, lymph node status, tumor size, stage, local relapse, distant metastatic relapse, ER status, PR status, and HER2 status. Histologic type was based on the TNM staging system, and the types were reclassified according to WHO classification and tumor stage (American Joint Committee on Cancer classification). The procedure for collecting and using the tissue samples was approved by the University of Malta, Research Ethical Committee, in accordance with the ethical standards as established in the Declaration of Helsinki.

### RNA-isolation and real-time RT-PCR for detection of miR-29b-1-5p and *SPIN1* in FFPE samples

Total RNA was isolated from FFPE tissues (five sections of 10 μm in thickness) using the RNA isolation kit FFPE (300115, Exiqon A/S, Vedbaek, Denmark) according to the manufacturer's instructions. All samples were analyzed on a Nanodrop 2000 instrument (Thermo Fisher Scientific, Waltham, USA).

For miR-29b-1-5p detection, cDNA samples were made out 40 ng of total RNA using the miRCURY LNA™ Universal RT microRNA PCR Universal cDNA Synthesis kit II (203301, Exiqon A/S) according to the manufacturer's recommendations. Afterward, real-time PCR was performed using 4μL of cDNA product, hsa-miR-29b-1-5p LNA™ primers (204261, Exiqon A/S) and ExiLENT SYBR Green master mix (203403, Exiqon A/S). PCR was performed under the following conditions: 95°C for 10 minutes, followed by 40 cycles of 95°C for 10 seconds and 60°C for 1 minute.

For *SPIN1* detection, 100 ng of total RNA was reverse transcribed by using the iScript™ cDNA Synthesis Kit (170-8890, Bio-Rad Laboratories S.r.l., Segrate, Milan, Italy), according to the manufacturer's instructions. The resulting cDNAs were used for quantitative analysis by real-time PCR (qPCR) using the IQ SYBR Green Supermix (170-8882, Bio-Rad) and the QuantiTect primers (Qiagen, Milan, Italy) for SPIN1 (QT00024584). PCR cycling was performed as follows: 95°C for 10min; 40 cycles of 95°C for 30 sec, 60°C for 60 sec, 72°C for 30 sec; a final extension at 72°C for 5 min.

All real-time PCR reactions were performed in triplicate. To ensure that the RNA samples were not contaminated with genomic DNA, we included a no reverse transcriptase control (no RT) during each run of real-time RT-PCR. Furthermore, to check the accuracy of amplifications, we included a negative control in each run by eliminating the cDNA sample in the tube. Real-time PCR and data collection were performed on an IQ5 cycler instrument (Bio-Rad Laboratories S.r.l., Segrate, Milan, Italy); qPCR data were analyzed by IQ5 cycler software.

For miR-29b-1-5p detection, hsa-miR-24-3p (204260, Exiqon A/S), hsa-miR-26b-3p (204117, Exiqon A/S) and U6 snRNA (203907, Exiqon A/S) were used as control genes. Hsa-miR-24-3p (mean Ct = 21.71, S.D. = 0.91) and hsa-miR-26b-3p (mean Ct = 26.83, S.D. = 0.93) showed the least variation between the 27 samples and were used as the endogenous reference. U6 was excluded because of too much variation (mean Ct = 22.75, S.D. = 2.03) between the different samples. The relative amount of *SPIN1* was normalized to *GAPDH* (QT01192646, Qiagen). Data were calculated using the comparative 2^−ΔΔCt^ method [55].

### Cell lines

Human Mammary Epithelial Cells (HMECs) were purchased from Lonza (Walkersville, MD, USA) and grown according to the manufacturer's instructions. Human TNBC cell lines MDA-MB-231 and MDA-MB-468 were obtained from Interlab Cell Line Collection (ICLC, National Institute of Cancer Research, Genoa, Italy); BT20 and HCC-1395 cell lines from America Type Culture Collection (ATCC, Manassas, VA, USA). All cells were maintained according to supplier's instructions and grown in an incubator at 37 °C in a humidified atmosphere containing 5% CO2.

### Mammosphere formation assay

MDA-MB-231, BT20, HCC-1395 and MDA-MB-468 cells were plated in 6-well ultra-low attachment plates (Corning Costar, Euroclone, Pero (MI), Italy) at appropriate density (5,000, 10,000, 15,000 and 20,000 viable cells per ml respectively) in a stem cell medium to form mammospheres as previously described [56]. The stem cell medium was changed every 3 days, and cells were observed every day by an inverted phase contrast microscope equipped with a computer-imaging system (Leica DM IRB, Leica Microsystems Srl, Milan, Italy). After incubation for 10 days, the number of mammospheres that were larger than 50 μm in diameter (determined using the ImageJ software) was counted. For propagation, mammospheres were collected by gentle centrifugation, dissociated to single cells and then cultured to generate mammospheres of the next generation. Three passages were performed at intervals of 10 days. Sphere formation efficiency (SFE) at each passage was calculated by dividing the total number of spheres formed by the total number of live cells seeded, multiplied by hundred.

### Plasmids and stable transfection

Vector construction for miR-29b-1 expression was previously described [22]. For stable transfection, MDA-MB-231 and BT20 cells were plated in 6-well dishes until they reached 90% confluence and then transfected with 3 μg of pCDHCMV-MCS-EF1-copGFP-T2A-PURO-miR-29b-1 or empty vector as a control (hereafter indicated as miR-29b-1 cells and control cells, respectively), using TransIT-X2™ Dynamic Delivery System (MIR 6003, Mirus Bio LLC, Madison, WI, USA) according to manufacturer's instructions. Two days after transfections the cells were transferred in 100 mm dishes in selective medium containing 0.5 μg/ml puromycin (sc-108071, Santa Cruz Biotechnology, Santa Cruz, CA, USA); the medium was replaced every 3-4 days. A plate of untransfected cells was used as a control for the selection. To assess transfection efficiency, green fluorescent protein (GFP) expressing cells were analyzed by fluorescence microscopy and flow cytometry as described previously [22].

For *SPIN1*-knockdown, MDA-MB-231 cells were allowed to grow till 90% confluency and then transfected with 2 μg of SPIN1 shRNA plasmid (sc-92696-SH, Santa Cruz Biotechnology) or Ctrl shRNA plasmid-A (sc-108060, Santa Cruz Biotechnology) using TransIT-X2™ Dynamic Delivery System (Mirus Bio LLC) according to manufacturer's instructions. For selection of stably transfected cells, we proceeded with puromycin selection as described above.

### Transient transfection

Both MDA-MB-231 and BT-20 cell lines were allowed to grow till 90% confluence and then transfected with a locked nucleic acid (LNA) probe containing a sequence specific antisense oligonucleotide targeting has-miR-29b-1-5p, miRCURY LNA™ miR-29b-1-5p Power Inhibitor (50 nM, 427008-8, Exiqon A/S). Transfection was performed using TransIT-X2™ Dynamic Delivery System (Mirus Bio LLC) according to manufacturer's instructions. A scrambled miRNA sequence, miRCURY LNA™ Power Inhibitor Control (50 nM, 199020-00, Exiqon A/S) served as a negative control. Cells were examined for each experiment 72 h after transfection.

### Cell proliferation, viability, apoptosis, Ki-67 expression and cell cycle analysis

Cellular growth and viability were evaluated by trypan blue (TB) exclusion assay as previously described [57]. Cell viability was also evaluated by propidium iodide (PI) exclusion test using flow cytometry assay. Similar to TB, PI has the ability to penetrate into cells that have lost plasma membrane integrity (dead cells) and to complex with DNA [58]. Briefly, cells were resuspended in 1 ml of PBS. PI (P4170, Sigma-Aldrich S.r.l., Milan, Italy) was added at 2 μg/ml for dead cell exclusion. Cells were incubated with the dye for at least 10 min at 4°C in the dark and submitted to flow cytometric analysis.

Apoptosis was evaluated using Annexin V-PE-Cy5 Detection Kit (KA0718, Abnova Corporation, Taipei City, Taiwan) according to manufacturer's instructions. Briefly, cells were trypsinized, centrifuged and washed once with serum-containing media before incubation with Annexin V-PE-Cy5. After another centrifugation, cells were resuspended in 1X Annexin V binding buffer at a concentration of 5 × 10^5^ cells/500μl. Cell suspensions were incubated with 5 μl of Annexin V-PE-Cy5 for 5 min at a room temperature in the dark. Unstained cells were used as negative control. Analysis was performed by flow cytometry.

Flow cytometric analysis of Ki-67 and cell cycle were performed as previously reported [22]

All flow cytometric analyses were performed by a COULTER EPICS XL (Beckman Coulter S.r.l., Cassina De Pecchi, Milan, Italy) equipped with a blue argon laser (488 nm). PI fluorescence was measured in the FL3 channel using a 620-nm BP filter and PE-Cy5 fluorescence was measured in the FL4 channel using a 675 nm BP filter. At least 1 × 10^4^ events were acquired. Data were analyzed by Expo 32 software (Beckman Coulter).

### *In vitro* scratch and invasion assays

MDA-MB-231 and BT-20 cells were seeded in 6-well plates at a density of 1 × 10^6^ cells/well in culture medium and grown to confluence. Then, cells were starved for 24 hours, and scraped with a 200-μl pipette tip to scratch the confluent cell monolayer. Culture medium was replaced with medium containing 0.1% FBS to minimize cell proliferation. Images of the wound area were captured by microscope (100x magnification) at the indicated time points. The extent of wound closure was determined by measuring through with the ImageJ software the area of cells that migrated into the wound and then dividing by the total area of wound.

Invasion assays were performed using 6-well invasion chamber system (Corning, Euroclone). MDA-MB-231 and BT-20 cells were seeded in the upper chamber at 1.5 × 10^5^ cells/well in culture medium serum-free. Culture medium containing 10% FBS (used as a chemoattractant) was placed in the bottom well. After 48 h, non migratory cells in the upper chamber were removed with a cotton-tip applicator, while invaded cells were counted and imaged by microscope after staining with Hoechst 33342 (2.5μg/ml, B2261, Sigma-Aldrich). The number of invading cells was determined by counting five high-powered fields (200x magnification) on each membrane.

### Paclitaxel treatment

Paclitaxel was purchased from Sigma (T1912, Sigma-Aldrich) and dissolved in dimethyl sulfoxide as previously reported [59]. Both MDA-MB-231 and BT-20 cells were cultured to 150,000 cells/well in 6 well plates (Corning Costar, Euroclone) in culture medium. After 24 h cells were treated with 50, 100 and 200 nM of paclitaxel for 24 h. Cellular growth, cell viability and cell cycle phase distribution were evaluated as described above.

### Bioinformatic prediction of miRNA targets

Predicted has-miR-29b-1-5p target genes were obtained using MIRDB database (http://mirdb.org). All the targets were predicted by the bioinformatics tool, MirTarget (developed by analyzing thousands of miRNA-target interactions from high-throughput sequencing experiments).

After selecting SPIN1 as predicted target of hsa-miR-29b-1-5p, the two target sites of SPIN1 3′UTR, predicted by MIRDB, were searched by the two widely used miRNA target prediction programs: Targetscan 7.0 (http://www.targetscan.org) and microRNA.org.

### Target protector analysis

The miScript Target Protector for the miR-29b-1-5p binding site in the 3′UTR of *SPIN1* mRNA was obtained from Qiagen (target binding site sequence provided: 5′-AAAUCACAGUUACUUCUAAACCAGAUUUCA-3′; MTP0077240). The miScript Target Protector is single-stranded, modified RNA that specially interferes with the interaction of a miRNA with a single target, while leaving the regulation of other targets of the same miRNA unaffected. miRCURY LNA™ miR-29b-1-5p Mimic (25 nM; 470851-001; Exiqon A/S) was transfected alone or co-transfected with miScript Target Protector (25 nM) into MDA-MB-231 and BT-20 cells according to the manufacturer's protocol. miRCURY LNA™ miR-29b-1-5p Mimic and Negative Control Target Protector (25 nM; MTP0000002; Qiagen) designed not to bind the mRNA of mammals were co-transfected into TNBC cell lines as a negative control. After 72 h of transfection, the total RNA was isolated and the mRNA expression levels of *SPIN1* were measured by real-time RT-PCR.

### RNA extraction and real-time RT-PCR for cell samples

Total RNA extraction, cDNA synthesis and real-time PCR for miRNAs and mRNAs detection were performed as previously described [22]. For mRNAs detection the following QuantiTect primers (Qiagen) were used: *CTNNB1* (QT00077882), *MYC* (QT00035406), *PTEN* (QT00035406), *AKT1* (QT00085379), *AKT2* (QT00085001), *AKT3* (QT00082138), *OCT4* (*POU5F1*: QT00210840), *SOX2* (QT00237601), *NANOG* (QT01025850), *CXCR4* (QT00223188), *VEGFA* (QT01010184), *H4* (*HIST4H4*: QT00218050) and *MKI67* (QT00014203). The relative amount of mRNAs and miRNAs was normalized to *GAPDH* (QT01192646) and *U6* snRNA (203907, Exiqon A/S), respectively. Data were calculated using the comparative 2^−ΔΔCt^ method [55].

### Western blot

Cell lysates and protein samples were prepared as previously reported [60]. Proteins were resolved by SDS-polyacrylamide gel electrophoresis and transferred to a nitrocellulose membrane (Bio-Rad) for detection with primary antibodies against SPIN1 (diluted 1:500, 19531-1-AP; Proteintech Group, Inc., Rosemont, IL, USA), CTNNB1 (diluted 1:500, sc-59737, Santa Cruz Biotechnology), MYC (diluted 1:500, sc-40, Santa Cruz Biotechnology), PTEN (diluted 1:500, sc-9145, Santa Cruz Biotechnology), AKT1/2/3 (diluted 1:500, sc-8312, Santa Cruz Biotechnology), p-AKT1/2/3 (Ser 473)-R (diluted 1:500, sc-7985-R, Santa Cruz Biotechnology), OCT 3/4 (diluted 1:300, sc-5279, Santa Cruz Biotechnology), SOX2 (diluted 1:300, sc-20088, Santa Cruz Biotechnology), NANOG (diluted 1:300, sc-293121, Santa Cruz Biotechnology) and ACTIN (diluted 1:500, A5060, Sigma-Aldrich). The membranes were then incubated with the appropriate horseradish peroxidase (HRP)-conjugated secondary antibodies (diluted 1:5000, Perce, Thermo Fisher Scientific). The protein bands were revealed with an enhanced chemiluminescence detection system (ECL; Bio-Rad) and visualized by ChemiDoc XRS system (Bio-Rad) and Quality One 4.5.2 (Biorad) software. Finally, protein levels were normalized using ACTIN levels. Protein bands were quantified densitometrically.

### Statistical analysis

Data were represented as mean ± S.D. The significance of the differences between groups was assessed with a two-tailed Student's t-test using Microsoft Excel. Differences were considered significant when P<0.05.

The relationship between miR-29b-1-5p and *SPIN1* expression was determined by evaluating Pearson's correlation coefficient (r). This coefficient, for continuous (interval level) data, ranges from −1 to +1. A correlation>0.8 is generally described as strong, whereas a correlation <0.5 is described as weak.

## SUPPLEMENTARY MATERIALS FIGURES AND TABLES







## References

[1] American Cancer Society (2013). Breast Cancer Facts & Figures 2013-2014.

[2] Foulkes WD, Smith IE, Reis-Filho JS (2010). Triple-negative breast cancer. N Engl J Med.

[3] Aysola K, Desai A, Welch C, Xu J, Qin Y, Reddy V, Matthews R, Owens C, Okoli J, Beech DJ, Piyathilake CJ, Reddy SP, Rao VN (2013). Triple Negative Breast Cancer - An Overview. Hereditary Genet.

[4] Hudis CA, Gianni L (2011). Triple-negative breast cancer: an unmet medical need. Oncologist.

[5] Clevers H (2011). The cancer stem cell: Premises, promises and challenges. Nat Med.

[6] Dick JE (2009). Looking ahead in cancer stem cell research. Nat Biotechnol.

[7] Drakaki A, Iliopoulos D (2009). MicroRNA Gene Networks in Oncogenesis. Current Genomics.

[8] Bartel DP (2009). MicroRNAs: target recognition and regulatory functions. Cell.

[9] Hatfield S, Ruohola-Baker H (2008). MicroRNA and stem cell function. Cell Tissue Res.

[10] Hatfield SD, Shcherbata HR, Fischer KA, Nakahara K, Carthew RW, Ruohola-Baker H (2005). Stem cell division is regulated by the microRNA pathway. Nature.

[11] Melo SA, Esteller M (2011). Dysregulation of microRNAs in cancer: Playing with fire. FEBS Lett.

[12] Wu Z, Huang X, Zou Q, Guo Y (2013). The inhibitory role of Mir-29 in growth of breast cancer cells. J Exp Clin Cancer Res.

[13] Chou J, Lin JH, Brenot A, Kim JW, Provot S, Werb Z (2013). GATA3 suppresses metastasis and modulates the tumour microenvironment by regulating microRNA-29b expression. Nat Cell Biol.

[14] Shinden Y, Iguchi T, Akiyoshi S, Ueo H, Ueda M, Hirata H, Sakimura S, Uchi R, Takano Y, Eguchi H, Sugimachi K, Kijima Y, Natsugoe S, Mimori K (2015). miR-29b is an indicator of prognosis in breast cancer patients. Mol Clin Oncol.

[15] Garzon R, Heaphy CE, Havelange V, Fabbri M, Volinia S, Tsao T, Zanesi N, Kornblau SM, Marcucci G, Calin GA, Andreeff M, Croce CM (2009). MicroRNA 29b functions in acute myeloid leukemia. Blood.

[16] Xiong Y, Fang JH, Yun JP, Yang J, Zhang Y, Jia WH, Zhuang SM (2010). Effects of microRNA-29 on apoptosis, tumorigenicity, and prognosis of hepatocellular carcinoma. Hepatology.

[17] Wang C, Bian Z, Wei D, Zhang JG (2011). MiR-29b regulates migration of human breast cancer cells. Mol Cell Biochem.

[18] Yan B, Guo Q, Fu FJ, Wang Z, Yin Z, Wei YB, Yang JR (2015). The role of miR-29b in cancer: regulation, function, and signaling. Onco Targets Ther.

[19] Cittelly DM, Finlay-Schultz J, Howe EN, Spoelstra NS, Axlund SD, Hendricks P, Jacobsen BM, Sartorius CA, Richer JK (2013). Progestin suppression of miR-29 potentiates dedifferentiation of breast cancer cells via KLF4. Oncogene.

[20] Jones KB, Salah Z, Del Mare S, Galasso M, Gaudio E, Nuovo GJ, Lovat F, LeBlanc K, Palatini J, Randall RL, Volinia S, Stein GS, Croce CM (2012). miRNA signatures associate with pathogenesis and progression of osteosarcoma. Cancer Res.

[21] Zhang W, Qian JX, Yi HL, Yang ZD, Wang CF, Chen JY, Wei XZ, Fu Q, Ma H (2012). The microRNA-29 plays a central role in osteosarcoma pathogenesis and progression. Mol Biol (Mosk).

[22] Di Fiore R, Drago-Ferrante R, Pentimalli F, Di Marzo D, Forte IM, D’Anneo A, Carlisi D, De Blasio A, Giuliano M, Tesoriere G, Giordano A, Vento R (2014). MicroRNA-29b-1 impairs *in vitro* cell proliferation, self-renewal and chemoresistance of human osteosarcoma 3AB-OS cancer stem cells. Int J Oncol.

[23] Di Fiore R, Santulli A, Ferrante RD, Giuliano M, De Blasio A, Messina C, Pirozzi G, Tirino V, Tesoriere G, Vento R (2009). Identification and expansion of human osteosarcoma-cancer-stem cells by long-term 3-aminobenzamide treatment. J Cell Physiol.

[24] Dontu G, Abdallah WM, Foley JM, Jackson KW, Clarke MF, Kawamura MJ, Wicha MS (2003). *In vitro* propagation and transcriptional profiling of human mammary stem/progenitor cells. Genes Dev.

[25] Ponti D, Costa A, Zaffaroni N, Pratesi G, Petrangolini G, Coradini D, Pilotti S, Pierotti MA, Daidone MG (2005). Isolation and *in vitro* propagation of tumorigenic breast cancer cells with stem/progenitor cell properties. Cancer Res.

[26] Franz H, Greschik H, Willmann D, Ozretić L, Jilg CA, Wardelmann E, Jung M, Buettner R, Schüle R (2015). The histone code reader SPIN1 controls RET signaling in liposarcoma. Oncotarget.

[27] Cai J, Guan H, Fang L, Yang Y, Zhu X, Yuan J, Wu J, Li M (2013). MicroRNA-374a activates Wnt/β-catenin signaling to promote breast cancer metastasis. J Clin Invest.

[28] Schaefer T, Wang H, Mir P, Konantz M, Pereboom TC, Paczulla AM, Merz B, Fehm T, Perner S, Rothfuss OC, Kanz L, Schulze-Osthoff K, Lengerke C (2015). Molecular and functional interactions between AKT and SOX2 in breast carcinoma. Oncotarget.

[29] von Minckwitz G, Untch M, Blohmer JU, Costa SD, Eidtmann H, Fasching PA, Gerber B, Eiermann W, Hilfrich J, Huober J, Jackisch C, Kaufmann M, Konecny GE (2012). Definition and impact of pathologic complete response on prognosis after neoadjuvant chemotherapy in various intrinsic breast cancer subtypes. J Clin Oncol.

[30] Heitz F, Harter P, Lueck HJ, Fissler-Eckhoff A, Lorenz-Salehi F, Scheil-Bertram S, Traut A, du Bois A (2009). Triple-negative and HER2-overexpressing breast cancers exhibit an elevated risk and an earlier occurrence of cerebral metastases. Eur J Cancer.

[31] Kim VN, Nam JW (2006). Genomics of microRNA. Trends Genet.

[32] Lin SL, Kim H, Ying SY (2008). Intron-mediated RNA interference and microRNA (miRNA). Front Biosci.

[33] Yang F, Zhang W, Shen Y, Guan X (2015). Identification of dysregulated microRNAs in triple-negative breast cancer (review). Int J Oncol.

[34] van Schooneveld E, Wildiers H, Vergote I, Vermeulen PB, Dirix LY, Van Laere SJ (2015). Dysregulation of microRNAs in breast cancer and their potential role as prognostic and predictive biomarkers in patient management. Breast Cancer Res.

[35] Gyparaki MT, Basdra EK, Papavassiliou AG (2014). MicroRNAs as regulatory elements in triple negative breast cancer. Cancer Lett.

[36] Ghelani HS, Rachchh MA, Gokani RH (2012). MicroRNAs as newer therapeutic targets: A big hope from a tiny player. J Pharmacol Pharmacother.

[37] Jansson MD, Lund AH (2012). MicroRNA and cancer. Mol Oncol.

[38] Zhang X, Zhao X, Fiskus W, Lin J, Lwin T, Rao R, Zhang Y, Chan JC, Fu K, Marquez VE, Chen-Kiang S, Moscinski LC, Seto E (2012). Coordinated silencing of MYC-mediated miR-29 by HDAC3 and EZH2 as a therapeutic target of histone modification in aggressive B-Cell lymphomas. Cancer Cell.

[39] Yan B, Guo Q, Fu FJ, Wang Z, Yin Z, Wei YB, Yang JR (2015). The role of miR-29b in cancer: regulation, function, and signaling. Onco Targets Ther.

[40] Kriegel AJ, Liu Y, Fang Y, Ding X, Liang M (2012). The miR-29 family: genomics, cell biology, and relevance to renal and cardiovascular injury. Physiol Genomics.

[41] Pekarsky Y, Croce CM (2010). Is miR-29 an oncogene or tumor suppressor in CLL?. Oncotarget.

[42] Wang B, Li W, Liu H, Yang L, Liao Q, Cui S, Wang H, Zhao L (2014). miR-29b suppresses tumor growth and metastasis in colorectal cancer via downregulating Tiam1 expression and inhibiting epithelial-mesenchymal transition. Cell Death Dis.

[43] Velasco-Velázquez MA, Homsi N, De La Fuente M, Pestell RG (2012). Breast cancer stem cells. Int J Biochem Cell Biol.

[44] Yeo JC, Ng HH (2013). The transcriptional regulation of pluripotency. Cell Res.

[45] Chavez KJ, Garimella SV, Lipkowitz S (2010). Triple negative breast cancer cell lines: one tool in the search for better treatment of triple negative breast cancer. Breast Dis.

[46] Cui SY, Wang R, Chen LB (2013). MicroRNAs: key players of taxane resistance and their therapeutic potential in human cancers. J Cell Mol Med.

[47] Conlin AK, Seidman AD (2007). Taxanes in breast cancer: an update. Curr Oncol Rep.

[48] Tang X, Jin L, Cao P, Cao K, Huang C, Luo Y, Ma J, Shen S, Tan M, Li X, Zhou M (2016). MicroRNA-16 sensitizes breast cancer cells to paclitaxel through suppression of IKBKB expression. Oncotarget.

[49] Wang JX, Zeng Q, Chen L, Du JC, Yan XL, Yuan HF, Zhai C, Zhou JN, Jia YL, Yue W, Pei XT (2012). SPINDLIN1 promotes cancer cell proliferation through activation of WNT/TCF-4 signaling. Mol Cancer Res.

[50] Bilir B, Kucuk O, Moreno CS (2013). Wnt signaling blockage inhibits cell proliferation and migration, and induces apoptosis in triple-negative breast cancer cells. J Transl Med.

[51] Tokunaga E, Kimura Y, Mashino K, Oki E, Kataoka A, Ohno S, Morita M, Kakeji Y, Baba H, Maehara Y (2006). Activation of PI3K/Akt signaling and hormone resistance in breast cancer. Breast Cancer.

[52] Horiuchi D, Kusdra L, Huskey NE, Chandriani S, Lenburg ME, Gonzalez-Angulo AM, Creasman KJ, Bazarov AV, Smyth JW, Davis SE, Yaswen P, Mills GB, Esserman LJ, Goga A (2012). MYC pathway activation in triple-negative breast cancer is synthetic lethal with CDK inhibition. J Exp Med.

[53] Mott JL, Kurita S, Cazanave SC, Bronk SF, Werneburg NW, Fernandez-Zapico ME (2010). Transcriptional suppression of mir-29b-1/mir-29a promoter by c-Myc, hedgehog, and NF-kappaB. J Cell Biochem.

[54] Liang Z, Brooks J, Willard M, Liang K, Yoon Y, Kang S, Shim H (2007). CXCR4/CXCL12 axis promotes VEGF-mediated tumor angiogenesis through Akt signaling pathway. Biochem Biophys Res Commun.

[55] Livak KJ, Schmittgen TD (2001). Analysis of relative gene expression data using real-time quantitative PCR and the 2(−Delta Delta C(T)) Method. Methods.

[56] Carlisi D, Buttitta G, Di Fiore R, Scerri C, Drago-Ferrante R, Vento R, Tesoriere G (2016). Parthenolide and DMAPT exert cytotoxic effects on breast cancer stem-like cells by inducing oxidative stress, mitochondrial dysfunction and necrosis. Cell Death Dis.

[57] Di Fiore R, Marcatti M, Drago-Ferrante R, D’Anneo A, Giuliano M, Carlisi D, De Blasio A, Querques F, Pastore L, Tesoriere G, Vento R (2014). Mutant p53 gain of function can be at the root of dedifferentiation of human osteosarcoma MG63 cells into 3AB-OS cancer stem cells. Bone.

[58] Avelar-Freitas BA, Almeida VG, Pinto MC, Mourão FA, Massensini AR, Martins-Filho OA, Rocha-Vieira E, Brito-Melo GE (2014). Trypan blue exclusion assay by flow cytometry. Braz J Med Biol Res.

[59] Drago-Ferrante R, Santulli A, Di Fiore R, Giuliano M, Calvaruso G, Tesoriere G, Vento R (2008). Low doses of paclitaxel potently induce apoptosis in human retinoblastoma Y79 cells by up-regulating E2F1. Int J Oncol.

[60] De Blasio A, Di Fiore R, Morreale M, Carlisi D, Drago-Ferrante R, Montalbano M, Scerri C, Tesoriere G, Vento R (2016). Unusual roles of caspase-8 in triple-negative breast cancer cell line MDA-MB-231. Int J Oncol.

