# Supplementary material for: Suppressive role exerted by microRNA-29b-1-5p in triple negative breast cancer through SPIN1 regulation
Source: Oncotarget. 2017 Mar 7;8(17):28939–58. doi: 10.18632/oncotarget.15960 (PMC5438704; doi:10.18632/oncotarget.15960)
Supplement: Supplementary file 1 [file oncotarget-08-28939-s001.pdf]

Suppressive role exerted by microRNA-29b-1-5p in triple negative breast cancer through SPIN1 regulation

SUPPLEMENTARY TABLES

Supplementary Table 1: 393 predicted targets for hsa-miR-29b-1-5p in miRDB

See Supplementary File 1

Supplementary Table 2: Clinicopathologic characteristics of patients

See Supplementary File 2
