# Supplementary material for: Suppressive role exerted by microRNA-29b-1-5p in triple negative breast cancer through SPIN1 regulation
Source: Oncotarget. 2017 Mar 7;8(17):28939–58. doi: 10.18632/oncotarget.15960 (PMC5438704; doi:10.18632/oncotarget.15960)
Supplement: Supplementary file 2 [file oncotarget-08-28939-s002.doc]

| Supplementary Table 1: **393 predicted targets for hsa-miR-29b-1-5p in miRDB** | | | | | |
| --- | --- | --- | --- | --- | --- |
| **Target Detail** | **Target Rank** | **Target Score** | **miRNA Name** | **Gene Symbol** | **Gene Description** |
| [Details](http://mirdb.org/cgi-bin/target_detail.cgi?targetID=1317275) | 1 | 100 | hsa-miR-29b-1-5p | [LIN9](http://www.ncbi.nlm.nih.gov/entrez/query.fcgi?db=gene&cmd=Retrieve&dopt=full_report&list_uids=286826) | lin-9 homolog (C. elegans) |
| [Details](http://mirdb.org/cgi-bin/target_detail.cgi?targetID=1316992) | 2 | 100 | hsa-miR-29b-1-5p | [USP28](http://www.ncbi.nlm.nih.gov/entrez/query.fcgi?db=gene&cmd=Retrieve&dopt=full_report&list_uids=57646) | ubiquitin specific peptidase 28 |
| [Details](http://mirdb.org/cgi-bin/target_detail.cgi?targetID=1317113) | 3 | 100 | hsa-miR-29b-1-5p | [PWWP2A](http://www.ncbi.nlm.nih.gov/entrez/query.fcgi?db=gene&cmd=Retrieve&dopt=full_report&list_uids=114825) | PWWP domain containing 2A |
| [Details](http://mirdb.org/cgi-bin/target_detail.cgi?targetID=1317269) | 4 | 99 | hsa-miR-29b-1-5p | [NEUROD1](http://www.ncbi.nlm.nih.gov/entrez/query.fcgi?db=gene&cmd=Retrieve&dopt=full_report&list_uids=4760) | neuronal differentiation 1 |
| [Details](http://mirdb.org/cgi-bin/target_detail.cgi?targetID=1317114) | 5 | 99 | hsa-miR-29b-1-5p | [FBXO33](http://www.ncbi.nlm.nih.gov/entrez/query.fcgi?db=gene&cmd=Retrieve&dopt=full_report&list_uids=254170) | F-box protein 33 |
| [Details](http://mirdb.org/cgi-bin/target_detail.cgi?targetID=1317108) | 6 | 99 | hsa-miR-29b-1-5p | [SPIN1](http://www.ncbi.nlm.nih.gov/entrez/query.fcgi?db=gene&cmd=Retrieve&dopt=full_report&list_uids=10927) | spindlin 1 |
| [Details](http://mirdb.org/cgi-bin/target_detail.cgi?targetID=1316995) | 7 | 99 | hsa-miR-29b-1-5p | [ZDHHC5](http://www.ncbi.nlm.nih.gov/entrez/query.fcgi?db=gene&cmd=Retrieve&dopt=full_report&list_uids=25921) | zinc finger, DHHC-type containing 5 |
| [Details](http://mirdb.org/cgi-bin/target_detail.cgi?targetID=1317012) | 8 | 98 | hsa-miR-29b-1-5p | [EWSR1](http://www.ncbi.nlm.nih.gov/entrez/query.fcgi?db=gene&cmd=Retrieve&dopt=full_report&list_uids=2130) | EWS RNA-binding protein 1 |
| [Details](http://mirdb.org/cgi-bin/target_detail.cgi?targetID=1316987) | 9 | 98 | hsa-miR-29b-1-5p | [MARK1](http://www.ncbi.nlm.nih.gov/entrez/query.fcgi?db=gene&cmd=Retrieve&dopt=full_report&list_uids=4139) | MAP/microtubule affinity-regulating kinase 1 |
| [Details](http://mirdb.org/cgi-bin/target_detail.cgi?targetID=1317211) | 10 | 98 | hsa-miR-29b-1-5p | [CGNL1](http://www.ncbi.nlm.nih.gov/entrez/query.fcgi?db=gene&cmd=Retrieve&dopt=full_report&list_uids=84952) | cingulin-like 1 |
| [Details](http://mirdb.org/cgi-bin/target_detail.cgi?targetID=1317343) | 11 | 98 | hsa-miR-29b-1-5p | [ACTC1](http://www.ncbi.nlm.nih.gov/entrez/query.fcgi?db=gene&cmd=Retrieve&dopt=full_report&list_uids=70) | actin, alpha, cardiac muscle 1 |
| [Details](http://mirdb.org/cgi-bin/target_detail.cgi?targetID=1317117) | 12 | 98 | hsa-miR-29b-1-5p | [MMD](http://www.ncbi.nlm.nih.gov/entrez/query.fcgi?db=gene&cmd=Retrieve&dopt=full_report&list_uids=23531) | monocyte to macrophage differentiation-associated |
| [Details](http://mirdb.org/cgi-bin/target_detail.cgi?targetID=1317088) | 13 | 97 | hsa-miR-29b-1-5p | [PAFAH1B2](http://www.ncbi.nlm.nih.gov/entrez/query.fcgi?db=gene&cmd=Retrieve&dopt=full_report&list_uids=5049) | platelet-activating factor acetylhydrolase 1b, catalytic subunit 2 (30kDa) |
| [Details](http://mirdb.org/cgi-bin/target_detail.cgi?targetID=1317232) | 14 | 97 | hsa-miR-29b-1-5p | [WDR26](http://www.ncbi.nlm.nih.gov/entrez/query.fcgi?db=gene&cmd=Retrieve&dopt=full_report&list_uids=80232) | WD repeat domain 26 |
| [Details](http://mirdb.org/cgi-bin/target_detail.cgi?targetID=1317195) | 15 | 97 | hsa-miR-29b-1-5p | [SOCS5](http://www.ncbi.nlm.nih.gov/entrez/query.fcgi?db=gene&cmd=Retrieve&dopt=full_report&list_uids=9655) | suppressor of cytokine signaling 5 |
| [Details](http://mirdb.org/cgi-bin/target_detail.cgi?targetID=1316991) | 16 | 96 | hsa-miR-29b-1-5p | [C15orf27](http://www.ncbi.nlm.nih.gov/entrez/query.fcgi?db=gene&cmd=Retrieve&dopt=full_report&list_uids=123591) | chromosome 15 open reading frame 27 |
| [Details](http://mirdb.org/cgi-bin/target_detail.cgi?targetID=1317241) | 17 | 96 | hsa-miR-29b-1-5p | [STC1](http://www.ncbi.nlm.nih.gov/entrez/query.fcgi?db=gene&cmd=Retrieve&dopt=full_report&list_uids=6781) | stanniocalcin 1 |
| [Details](http://mirdb.org/cgi-bin/target_detail.cgi?targetID=1317279) | 18 | 96 | hsa-miR-29b-1-5p | [TECPR2](http://www.ncbi.nlm.nih.gov/entrez/query.fcgi?db=gene&cmd=Retrieve&dopt=full_report&list_uids=9895) | tectonin beta-propeller repeat containing 2 |
| [Details](http://mirdb.org/cgi-bin/target_detail.cgi?targetID=1317087) | 19 | 96 | hsa-miR-29b-1-5p | [LHFPL2](http://www.ncbi.nlm.nih.gov/entrez/query.fcgi?db=gene&cmd=Retrieve&dopt=full_report&list_uids=10184) | lipoma HMGIC fusion partner-like 2 |
| [Details](http://mirdb.org/cgi-bin/target_detail.cgi?targetID=1317177) | 20 | 96 | hsa-miR-29b-1-5p | [FA2H](http://www.ncbi.nlm.nih.gov/entrez/query.fcgi?db=gene&cmd=Retrieve&dopt=full_report&list_uids=79152) | fatty acid 2-hydroxylase |
| [Details](http://mirdb.org/cgi-bin/target_detail.cgi?targetID=1317169) | 21 | 96 | hsa-miR-29b-1-5p | [UQCC1](http://www.ncbi.nlm.nih.gov/entrez/query.fcgi?db=gene&cmd=Retrieve&dopt=full_report&list_uids=55245) | ubiquinol-cytochrome c reductase complex assembly factor 1 |
| [Details](http://mirdb.org/cgi-bin/target_detail.cgi?targetID=1317106) | 22 | 95 | hsa-miR-29b-1-5p | [HIPK1](http://www.ncbi.nlm.nih.gov/entrez/query.fcgi?db=gene&cmd=Retrieve&dopt=full_report&list_uids=204851) | homeodomain interacting protein kinase 1 |
| [Details](http://mirdb.org/cgi-bin/target_detail.cgi?targetID=1316963) | 23 | 95 | hsa-miR-29b-1-5p | [TNPO3](http://www.ncbi.nlm.nih.gov/entrez/query.fcgi?db=gene&cmd=Retrieve&dopt=full_report&list_uids=23534) | transportin 3 |
| [Details](http://mirdb.org/cgi-bin/target_detail.cgi?targetID=1317220) | 24 | 95 | hsa-miR-29b-1-5p | [NR2C2](http://www.ncbi.nlm.nih.gov/entrez/query.fcgi?db=gene&cmd=Retrieve&dopt=full_report&list_uids=7182) | nuclear receptor subfamily 2, group C, member 2 |
| [Details](http://mirdb.org/cgi-bin/target_detail.cgi?targetID=1316998) | 25 | 95 | hsa-miR-29b-1-5p | [PAK7](http://www.ncbi.nlm.nih.gov/entrez/query.fcgi?db=gene&cmd=Retrieve&dopt=full_report&list_uids=57144) | p21 protein (Cdc42/Rac)-activated kinase 7 |
| [Details](http://mirdb.org/cgi-bin/target_detail.cgi?targetID=1317010) | 26 | 94 | hsa-miR-29b-1-5p | [FAM84A](http://www.ncbi.nlm.nih.gov/entrez/query.fcgi?db=gene&cmd=Retrieve&dopt=full_report&list_uids=151354) | family with sequence similarity 84, member A |
| [Details](http://mirdb.org/cgi-bin/target_detail.cgi?targetID=1317047) | 27 | 94 | hsa-miR-29b-1-5p | [DIXDC1](http://www.ncbi.nlm.nih.gov/entrez/query.fcgi?db=gene&cmd=Retrieve&dopt=full_report&list_uids=85458) | DIX domain containing 1 |
| [Details](http://mirdb.org/cgi-bin/target_detail.cgi?targetID=1317006) | 28 | 94 | hsa-miR-29b-1-5p | [USP39](http://www.ncbi.nlm.nih.gov/entrez/query.fcgi?db=gene&cmd=Retrieve&dopt=full_report&list_uids=10713) | ubiquitin specific peptidase 39 |
| [Details](http://mirdb.org/cgi-bin/target_detail.cgi?targetID=1317170) | 29 | 94 | hsa-miR-29b-1-5p | [PLA2R1](http://www.ncbi.nlm.nih.gov/entrez/query.fcgi?db=gene&cmd=Retrieve&dopt=full_report&list_uids=22925) | phospholipase A2 receptor 1, 180kDa |
| [Details](http://mirdb.org/cgi-bin/target_detail.cgi?targetID=1317274) | 30 | 94 | hsa-miR-29b-1-5p | [EXT1](http://www.ncbi.nlm.nih.gov/entrez/query.fcgi?db=gene&cmd=Retrieve&dopt=full_report&list_uids=2131) | exostosin glycosyltransferase 1 |
| [Details](http://mirdb.org/cgi-bin/target_detail.cgi?targetID=1316959) | 31 | 94 | hsa-miR-29b-1-5p | [FAM222B](http://www.ncbi.nlm.nih.gov/entrez/query.fcgi?db=gene&cmd=Retrieve&dopt=full_report&list_uids=55731) | family with sequence similarity 222, member B |
| [Details](http://mirdb.org/cgi-bin/target_detail.cgi?targetID=1317096) | 32 | 94 | hsa-miR-29b-1-5p | [PANK3](http://www.ncbi.nlm.nih.gov/entrez/query.fcgi?db=gene&cmd=Retrieve&dopt=full_report&list_uids=79646) | pantothenate kinase 3 |
| [Details](http://mirdb.org/cgi-bin/target_detail.cgi?targetID=1317228) | 33 | 94 | hsa-miR-29b-1-5p | [TMTC2](http://www.ncbi.nlm.nih.gov/entrez/query.fcgi?db=gene&cmd=Retrieve&dopt=full_report&list_uids=160335) | transmembrane and tetratricopeptide repeat containing 2 |
| [Details](http://mirdb.org/cgi-bin/target_detail.cgi?targetID=1317326) | 34 | 94 | hsa-miR-29b-1-5p | [HOXC4](http://www.ncbi.nlm.nih.gov/entrez/query.fcgi?db=gene&cmd=Retrieve&dopt=full_report&list_uids=3221) | homeobox C4 |
| [Details](http://mirdb.org/cgi-bin/target_detail.cgi?targetID=1317101) | 35 | 94 | hsa-miR-29b-1-5p | [FOLR1](http://www.ncbi.nlm.nih.gov/entrez/query.fcgi?db=gene&cmd=Retrieve&dopt=full_report&list_uids=2348) | folate receptor 1 (adult) |
| [Details](http://mirdb.org/cgi-bin/target_detail.cgi?targetID=1317286) | 36 | 93 | hsa-miR-29b-1-5p | [HMBS](http://www.ncbi.nlm.nih.gov/entrez/query.fcgi?db=gene&cmd=Retrieve&dopt=full_report&list_uids=3145) | hydroxymethylbilane synthase |
| [Details](http://mirdb.org/cgi-bin/target_detail.cgi?targetID=1317304) | 37 | 93 | hsa-miR-29b-1-5p | [GABRB2](http://www.ncbi.nlm.nih.gov/entrez/query.fcgi?db=gene&cmd=Retrieve&dopt=full_report&list_uids=2561) | gamma-aminobutyric acid (GABA) A receptor, beta 2 |
| [Details](http://mirdb.org/cgi-bin/target_detail.cgi?targetID=1317104) | 38 | 93 | hsa-miR-29b-1-5p | [NR3C1](http://www.ncbi.nlm.nih.gov/entrez/query.fcgi?db=gene&cmd=Retrieve&dopt=full_report&list_uids=2908) | nuclear receptor subfamily 3, group C, member 1 (glucocorticoid receptor) |
| [Details](http://mirdb.org/cgi-bin/target_detail.cgi?targetID=1317260) | 39 | 93 | hsa-miR-29b-1-5p | [ASB7](http://www.ncbi.nlm.nih.gov/entrez/query.fcgi?db=gene&cmd=Retrieve&dopt=full_report&list_uids=140460) | ankyrin repeat and SOCS box containing 7 |
| [Details](http://mirdb.org/cgi-bin/target_detail.cgi?targetID=1317159) | 40 | 93 | hsa-miR-29b-1-5p | [TIPARP](http://www.ncbi.nlm.nih.gov/entrez/query.fcgi?db=gene&cmd=Retrieve&dopt=full_report&list_uids=25976) | TCDD-inducible poly(ADP-ribose) polymerase |
| [Details](http://mirdb.org/cgi-bin/target_detail.cgi?targetID=1317244) | 41 | 93 | hsa-miR-29b-1-5p | [CA13](http://www.ncbi.nlm.nih.gov/entrez/query.fcgi?db=gene&cmd=Retrieve&dopt=full_report&list_uids=377677) | carbonic anhydrase XIII |
| [Details](http://mirdb.org/cgi-bin/target_detail.cgi?targetID=1317296) | 42 | 92 | hsa-miR-29b-1-5p | [CNEP1R1](http://www.ncbi.nlm.nih.gov/entrez/query.fcgi?db=gene&cmd=Retrieve&dopt=full_report&list_uids=255919) | CTD nuclear envelope phosphatase 1 regulatory subunit 1 |
| [Details](http://mirdb.org/cgi-bin/target_detail.cgi?targetID=1317254) | 43 | 91 | hsa-miR-29b-1-5p | [MYO1B](http://www.ncbi.nlm.nih.gov/entrez/query.fcgi?db=gene&cmd=Retrieve&dopt=full_report&list_uids=4430) | myosin IB |
| [Details](http://mirdb.org/cgi-bin/target_detail.cgi?targetID=1317105) | 44 | 91 | hsa-miR-29b-1-5p | [NUAK2](http://www.ncbi.nlm.nih.gov/entrez/query.fcgi?db=gene&cmd=Retrieve&dopt=full_report&list_uids=81788) | NUAK family, SNF1-like kinase, 2 |
| [Details](http://mirdb.org/cgi-bin/target_detail.cgi?targetID=1317302) | 45 | 91 | hsa-miR-29b-1-5p | [ZFAND5](http://www.ncbi.nlm.nih.gov/entrez/query.fcgi?db=gene&cmd=Retrieve&dopt=full_report&list_uids=7763) | zinc finger, AN1-type domain 5 |
| [Details](http://mirdb.org/cgi-bin/target_detail.cgi?targetID=1317079) | 46 | 90 | hsa-miR-29b-1-5p | [PPP1R15B](http://www.ncbi.nlm.nih.gov/entrez/query.fcgi?db=gene&cmd=Retrieve&dopt=full_report&list_uids=84919) | protein phosphatase 1, regulatory subunit 15B |
| [Details](http://mirdb.org/cgi-bin/target_detail.cgi?targetID=1317063) | 47 | 90 | hsa-miR-29b-1-5p | [AZIN1](http://www.ncbi.nlm.nih.gov/entrez/query.fcgi?db=gene&cmd=Retrieve&dopt=full_report&list_uids=51582) | antizyme inhibitor 1 |
| [Details](http://mirdb.org/cgi-bin/target_detail.cgi?targetID=1316969) | 48 | 90 | hsa-miR-29b-1-5p | [KIAA1598](http://www.ncbi.nlm.nih.gov/entrez/query.fcgi?db=gene&cmd=Retrieve&dopt=full_report&list_uids=57698) | KIAA1598 |
| [Details](http://mirdb.org/cgi-bin/target_detail.cgi?targetID=1317024) | 49 | 89 | hsa-miR-29b-1-5p | [RABGAP1](http://www.ncbi.nlm.nih.gov/entrez/query.fcgi?db=gene&cmd=Retrieve&dopt=full_report&list_uids=23637) | RAB GTPase activating protein 1 |
| [Details](http://mirdb.org/cgi-bin/target_detail.cgi?targetID=1317035) | 50 | 89 | hsa-miR-29b-1-5p | [SLC30A7](http://www.ncbi.nlm.nih.gov/entrez/query.fcgi?db=gene&cmd=Retrieve&dopt=full_report&list_uids=148867) | solute carrier family 30 (zinc transporter), member 7 |
| [Details](http://mirdb.org/cgi-bin/target_detail.cgi?targetID=1317103) | 51 | 89 | hsa-miR-29b-1-5p | [MLEC](http://www.ncbi.nlm.nih.gov/entrez/query.fcgi?db=gene&cmd=Retrieve&dopt=full_report&list_uids=9761) | malectin |
| [Details](http://mirdb.org/cgi-bin/target_detail.cgi?targetID=1317317) | 52 | 89 | hsa-miR-29b-1-5p | [LYRM1](http://www.ncbi.nlm.nih.gov/entrez/query.fcgi?db=gene&cmd=Retrieve&dopt=full_report&list_uids=57149) | LYR motif containing 1 |
| [Details](http://mirdb.org/cgi-bin/target_detail.cgi?targetID=1317060) | 53 | 89 | hsa-miR-29b-1-5p | [ALDH6A1](http://www.ncbi.nlm.nih.gov/entrez/query.fcgi?db=gene&cmd=Retrieve&dopt=full_report&list_uids=4329) | aldehyde dehydrogenase 6 family, member A1 |
| [Details](http://mirdb.org/cgi-bin/target_detail.cgi?targetID=1317192) | 54 | 89 | hsa-miR-29b-1-5p | [USP4](http://www.ncbi.nlm.nih.gov/entrez/query.fcgi?db=gene&cmd=Retrieve&dopt=full_report&list_uids=7375) | ubiquitin specific peptidase 4 (proto-oncogene) |
| [Details](http://mirdb.org/cgi-bin/target_detail.cgi?targetID=1317298) | 55 | 88 | hsa-miR-29b-1-5p | [RUNDC3A](http://www.ncbi.nlm.nih.gov/entrez/query.fcgi?db=gene&cmd=Retrieve&dopt=full_report&list_uids=10900) | RUN domain containing 3A |
| [Details](http://mirdb.org/cgi-bin/target_detail.cgi?targetID=1317294) | 56 | 88 | hsa-miR-29b-1-5p | [MLANA](http://www.ncbi.nlm.nih.gov/entrez/query.fcgi?db=gene&cmd=Retrieve&dopt=full_report&list_uids=2315) | melan-A |
| [Details](http://mirdb.org/cgi-bin/target_detail.cgi?targetID=1317100) | 57 | 88 | hsa-miR-29b-1-5p | [SOS2](http://www.ncbi.nlm.nih.gov/entrez/query.fcgi?db=gene&cmd=Retrieve&dopt=full_report&list_uids=6655) | son of sevenless homolog 2 (Drosophila) |
| [Details](http://mirdb.org/cgi-bin/target_detail.cgi?targetID=1317019) | 58 | 88 | hsa-miR-29b-1-5p | [ELL2](http://www.ncbi.nlm.nih.gov/entrez/query.fcgi?db=gene&cmd=Retrieve&dopt=full_report&list_uids=22936) | elongation factor, RNA polymerase II, 2 |
| [Details](http://mirdb.org/cgi-bin/target_detail.cgi?targetID=1317053) | 59 | 88 | hsa-miR-29b-1-5p | [NUP153](http://www.ncbi.nlm.nih.gov/entrez/query.fcgi?db=gene&cmd=Retrieve&dopt=full_report&list_uids=9972) | nucleoporin 153kDa |
| [Details](http://mirdb.org/cgi-bin/target_detail.cgi?targetID=1316970) | 60 | 88 | hsa-miR-29b-1-5p | [EP300](http://www.ncbi.nlm.nih.gov/entrez/query.fcgi?db=gene&cmd=Retrieve&dopt=full_report&list_uids=2033) | E1A binding protein p300 |
| [Details](http://mirdb.org/cgi-bin/target_detail.cgi?targetID=1317027) | 61 | 88 | hsa-miR-29b-1-5p | [ATF6](http://www.ncbi.nlm.nih.gov/entrez/query.fcgi?db=gene&cmd=Retrieve&dopt=full_report&list_uids=22926) | activating transcription factor 6 |
| [Details](http://mirdb.org/cgi-bin/target_detail.cgi?targetID=1317253) | 62 | 88 | hsa-miR-29b-1-5p | [RNF139](http://www.ncbi.nlm.nih.gov/entrez/query.fcgi?db=gene&cmd=Retrieve&dopt=full_report&list_uids=11236) | ring finger protein 139 |
| [Details](http://mirdb.org/cgi-bin/target_detail.cgi?targetID=1317320) | 63 | 88 | hsa-miR-29b-1-5p | [SLMO2](http://www.ncbi.nlm.nih.gov/entrez/query.fcgi?db=gene&cmd=Retrieve&dopt=full_report&list_uids=51012) | slowmo homolog 2 (Drosophila) |
| [Details](http://mirdb.org/cgi-bin/target_detail.cgi?targetID=1317339) | 64 | 88 | hsa-miR-29b-1-5p | [LOC400682](http://www.ncbi.nlm.nih.gov/entrez/query.fcgi?db=gene&cmd=Retrieve&dopt=full_report&list_uids=400682) | zinc finger protein 100-like |
| [Details](http://mirdb.org/cgi-bin/target_detail.cgi?targetID=1317239) | 65 | 87 | hsa-miR-29b-1-5p | [EYA4](http://www.ncbi.nlm.nih.gov/entrez/query.fcgi?db=gene&cmd=Retrieve&dopt=full_report&list_uids=2070) | eyes absent homolog 4 (Drosophila) |
| [Details](http://mirdb.org/cgi-bin/target_detail.cgi?targetID=1317085) | 66 | 87 | hsa-miR-29b-1-5p | [COL4A3BP](http://www.ncbi.nlm.nih.gov/entrez/query.fcgi?db=gene&cmd=Retrieve&dopt=full_report&list_uids=10087) | collagen, type IV, alpha 3 (Goodpasture antigen) binding protein |
| [Details](http://mirdb.org/cgi-bin/target_detail.cgi?targetID=1317201) | 67 | 87 | hsa-miR-29b-1-5p | [RIMS4](http://www.ncbi.nlm.nih.gov/entrez/query.fcgi?db=gene&cmd=Retrieve&dopt=full_report&list_uids=140730) | regulating synaptic membrane exocytosis 4 |
| [Details](http://mirdb.org/cgi-bin/target_detail.cgi?targetID=1317173) | 68 | 87 | hsa-miR-29b-1-5p | [KLF12](http://www.ncbi.nlm.nih.gov/entrez/query.fcgi?db=gene&cmd=Retrieve&dopt=full_report&list_uids=11278) | Kruppel-like factor 12 |
| [Details](http://mirdb.org/cgi-bin/target_detail.cgi?targetID=1317300) | 69 | 87 | hsa-miR-29b-1-5p | [SLC22A15](http://www.ncbi.nlm.nih.gov/entrez/query.fcgi?db=gene&cmd=Retrieve&dopt=full_report&list_uids=55356) | solute carrier family 22, member 15 |
| [Details](http://mirdb.org/cgi-bin/target_detail.cgi?targetID=1317175) | 70 | 87 | hsa-miR-29b-1-5p | [SLC35F6](http://www.ncbi.nlm.nih.gov/entrez/query.fcgi?db=gene&cmd=Retrieve&dopt=full_report&list_uids=54978) | solute carrier family 35, member F6 |
| [Details](http://mirdb.org/cgi-bin/target_detail.cgi?targetID=1317208) | 71 | 87 | hsa-miR-29b-1-5p | [FAM63B](http://www.ncbi.nlm.nih.gov/entrez/query.fcgi?db=gene&cmd=Retrieve&dopt=full_report&list_uids=54629) | family with sequence similarity 63, member B |
| [Details](http://mirdb.org/cgi-bin/target_detail.cgi?targetID=1317207) | 72 | 86 | hsa-miR-29b-1-5p | [ARGLU1](http://www.ncbi.nlm.nih.gov/entrez/query.fcgi?db=gene&cmd=Retrieve&dopt=full_report&list_uids=55082) | arginine and glutamate rich 1 |
| [Details](http://mirdb.org/cgi-bin/target_detail.cgi?targetID=1317037) | 73 | 86 | hsa-miR-29b-1-5p | [OPCML](http://www.ncbi.nlm.nih.gov/entrez/query.fcgi?db=gene&cmd=Retrieve&dopt=full_report&list_uids=4978) | opioid binding protein/cell adhesion molecule-like |
| [Details](http://mirdb.org/cgi-bin/target_detail.cgi?targetID=1317313) | 74 | 86 | hsa-miR-29b-1-5p | [PRRX1](http://www.ncbi.nlm.nih.gov/entrez/query.fcgi?db=gene&cmd=Retrieve&dopt=full_report&list_uids=5396) | paired related homeobox 1 |
| [Details](http://mirdb.org/cgi-bin/target_detail.cgi?targetID=1317187) | 75 | 86 | hsa-miR-29b-1-5p | [LUZP1](http://www.ncbi.nlm.nih.gov/entrez/query.fcgi?db=gene&cmd=Retrieve&dopt=full_report&list_uids=7798) | leucine zipper protein 1 |
| [Details](http://mirdb.org/cgi-bin/target_detail.cgi?targetID=1317102) | 76 | 86 | hsa-miR-29b-1-5p | [C1orf43](http://www.ncbi.nlm.nih.gov/entrez/query.fcgi?db=gene&cmd=Retrieve&dopt=full_report&list_uids=25912) | chromosome 1 open reading frame 43 |
| [Details](http://mirdb.org/cgi-bin/target_detail.cgi?targetID=1317226) | 77 | 86 | hsa-miR-29b-1-5p | [PROS1](http://www.ncbi.nlm.nih.gov/entrez/query.fcgi?db=gene&cmd=Retrieve&dopt=full_report&list_uids=5627) | protein S (alpha) |
| [Details](http://mirdb.org/cgi-bin/target_detail.cgi?targetID=1317284) | 78 | 86 | hsa-miR-29b-1-5p | [PCSK2](http://www.ncbi.nlm.nih.gov/entrez/query.fcgi?db=gene&cmd=Retrieve&dopt=full_report&list_uids=5126) | proprotein convertase subtilisin/kexin type 2 |
| [Details](http://mirdb.org/cgi-bin/target_detail.cgi?targetID=1317194) | 79 | 86 | hsa-miR-29b-1-5p | [EDIL3](http://www.ncbi.nlm.nih.gov/entrez/query.fcgi?db=gene&cmd=Retrieve&dopt=full_report&list_uids=10085) | EGF-like repeats and discoidin I-like domains 3 |
| [Details](http://mirdb.org/cgi-bin/target_detail.cgi?targetID=1317312) | 80 | 85 | hsa-miR-29b-1-5p | [CLEC2B](http://www.ncbi.nlm.nih.gov/entrez/query.fcgi?db=gene&cmd=Retrieve&dopt=full_report&list_uids=9976) | C-type lectin domain family 2, member B |
| [Details](http://mirdb.org/cgi-bin/target_detail.cgi?targetID=1317219) | 81 | 85 | hsa-miR-29b-1-5p | [DCAF5](http://www.ncbi.nlm.nih.gov/entrez/query.fcgi?db=gene&cmd=Retrieve&dopt=full_report&list_uids=8816) | DDB1 and CUL4 associated factor 5 |
| [Details](http://mirdb.org/cgi-bin/target_detail.cgi?targetID=1317265) | 82 | 85 | hsa-miR-29b-1-5p | [LRCH1](http://www.ncbi.nlm.nih.gov/entrez/query.fcgi?db=gene&cmd=Retrieve&dopt=full_report&list_uids=23143) | leucine-rich repeats and calponin homology (CH) domain containing 1 |
| [Details](http://mirdb.org/cgi-bin/target_detail.cgi?targetID=1317080) | 83 | 85 | hsa-miR-29b-1-5p | [DENND5A](http://www.ncbi.nlm.nih.gov/entrez/query.fcgi?db=gene&cmd=Retrieve&dopt=full_report&list_uids=23258) | DENN/MADD domain containing 5A |
| [Details](http://mirdb.org/cgi-bin/target_detail.cgi?targetID=1317001) | 84 | 85 | hsa-miR-29b-1-5p | [GCNT1](http://www.ncbi.nlm.nih.gov/entrez/query.fcgi?db=gene&cmd=Retrieve&dopt=full_report&list_uids=2650) | glucosaminyl (N-acetyl) transferase 1, core 2 |
| [Details](http://mirdb.org/cgi-bin/target_detail.cgi?targetID=1317236) | 85 | 85 | hsa-miR-29b-1-5p | [ARL17A](http://www.ncbi.nlm.nih.gov/entrez/query.fcgi?db=gene&cmd=Retrieve&dopt=full_report&list_uids=51326) | ADP-ribosylation factor-like 17A |
| [Details](http://mirdb.org/cgi-bin/target_detail.cgi?targetID=1317270) | 86 | 85 | hsa-miR-29b-1-5p | [NEURL1B](http://www.ncbi.nlm.nih.gov/entrez/query.fcgi?db=gene&cmd=Retrieve&dopt=full_report&list_uids=54492) | neuralized E3 ubiquitin protein ligase 1B |
| [Details](http://mirdb.org/cgi-bin/target_detail.cgi?targetID=1317276) | 87 | 84 | hsa-miR-29b-1-5p | [ETV6](http://www.ncbi.nlm.nih.gov/entrez/query.fcgi?db=gene&cmd=Retrieve&dopt=full_report&list_uids=2120) | ets variant 6 |
| [Details](http://mirdb.org/cgi-bin/target_detail.cgi?targetID=1317327) | 88 | 84 | hsa-miR-29b-1-5p | [LTBP1](http://www.ncbi.nlm.nih.gov/entrez/query.fcgi?db=gene&cmd=Retrieve&dopt=full_report&list_uids=4052) | latent transforming growth factor beta binding protein 1 |
| [Details](http://mirdb.org/cgi-bin/target_detail.cgi?targetID=1317051) | 89 | 84 | hsa-miR-29b-1-5p | [PHF20L1](http://www.ncbi.nlm.nih.gov/entrez/query.fcgi?db=gene&cmd=Retrieve&dopt=full_report&list_uids=51105) | PHD finger protein 20-like 1 |
| [Details](http://mirdb.org/cgi-bin/target_detail.cgi?targetID=1317196) | 90 | 84 | hsa-miR-29b-1-5p | [SMARCA5](http://www.ncbi.nlm.nih.gov/entrez/query.fcgi?db=gene&cmd=Retrieve&dopt=full_report&list_uids=8467) | SWI/SNF related, matrix associated, actin dependent regulator of chromatin, subfamily a, member 5 |
| [Details](http://mirdb.org/cgi-bin/target_detail.cgi?targetID=1317026) | 91 | 84 | hsa-miR-29b-1-5p | [SLC4A7](http://www.ncbi.nlm.nih.gov/entrez/query.fcgi?db=gene&cmd=Retrieve&dopt=full_report&list_uids=9497) | solute carrier family 4, sodium bicarbonate cotransporter, member 7 |
| [Details](http://mirdb.org/cgi-bin/target_detail.cgi?targetID=1317314) | 92 | 84 | hsa-miR-29b-1-5p | [CDH10](http://www.ncbi.nlm.nih.gov/entrez/query.fcgi?db=gene&cmd=Retrieve&dopt=full_report&list_uids=1008) | cadherin 10, type 2 (T2-cadherin) |
| [Details](http://mirdb.org/cgi-bin/target_detail.cgi?targetID=1317121) | 93 | 84 | hsa-miR-29b-1-5p | [TBC1D30](http://www.ncbi.nlm.nih.gov/entrez/query.fcgi?db=gene&cmd=Retrieve&dopt=full_report&list_uids=23329) | TBC1 domain family, member 30 |
| [Details](http://mirdb.org/cgi-bin/target_detail.cgi?targetID=1317032) | 94 | 84 | hsa-miR-29b-1-5p | [TMEM127](http://www.ncbi.nlm.nih.gov/entrez/query.fcgi?db=gene&cmd=Retrieve&dopt=full_report&list_uids=55654) | transmembrane protein 127 |
| [Details](http://mirdb.org/cgi-bin/target_detail.cgi?targetID=1317266) | 95 | 83 | hsa-miR-29b-1-5p | [PHTF2](http://www.ncbi.nlm.nih.gov/entrez/query.fcgi?db=gene&cmd=Retrieve&dopt=full_report&list_uids=57157) | putative homeodomain transcription factor 2 |
| [Details](http://mirdb.org/cgi-bin/target_detail.cgi?targetID=1317290) | 96 | 83 | hsa-miR-29b-1-5p | [PCDHB12](http://www.ncbi.nlm.nih.gov/entrez/query.fcgi?db=gene&cmd=Retrieve&dopt=full_report&list_uids=56124) | protocadherin beta 12 |
| [Details](http://mirdb.org/cgi-bin/target_detail.cgi?targetID=1316973) | 97 | 83 | hsa-miR-29b-1-5p | [NSFL1C](http://www.ncbi.nlm.nih.gov/entrez/query.fcgi?db=gene&cmd=Retrieve&dopt=full_report&list_uids=55968) | NSFL1 (p97) cofactor (p47) |
| [Details](http://mirdb.org/cgi-bin/target_detail.cgi?targetID=1317341) | 98 | 83 | hsa-miR-29b-1-5p | [GOLPH3](http://www.ncbi.nlm.nih.gov/entrez/query.fcgi?db=gene&cmd=Retrieve&dopt=full_report&list_uids=64083) | golgi phosphoprotein 3 (coat-protein) |
| [Details](http://mirdb.org/cgi-bin/target_detail.cgi?targetID=1317129) | 99 | 83 | hsa-miR-29b-1-5p | [DICER1](http://www.ncbi.nlm.nih.gov/entrez/query.fcgi?db=gene&cmd=Retrieve&dopt=full_report&list_uids=23405) | dicer 1, ribonuclease type III |
| [Details](http://mirdb.org/cgi-bin/target_detail.cgi?targetID=1316999) | 100 | 83 | hsa-miR-29b-1-5p | [TMX3](http://www.ncbi.nlm.nih.gov/entrez/query.fcgi?db=gene&cmd=Retrieve&dopt=full_report&list_uids=54495) | thioredoxin-related transmembrane protein 3 |
| [Details](http://mirdb.org/cgi-bin/target_detail.cgi?targetID=1317115) | 101 | 83 | hsa-miR-29b-1-5p | [REM1](http://www.ncbi.nlm.nih.gov/entrez/query.fcgi?db=gene&cmd=Retrieve&dopt=full_report&list_uids=28954) | RAS (RAD and GEM)-like GTP-binding 1 |
| [Details](http://mirdb.org/cgi-bin/target_detail.cgi?targetID=1317167) | 102 | 83 | hsa-miR-29b-1-5p | [CLIC4](http://www.ncbi.nlm.nih.gov/entrez/query.fcgi?db=gene&cmd=Retrieve&dopt=full_report&list_uids=25932) | chloride intracellular channel 4 |
| [Details](http://mirdb.org/cgi-bin/target_detail.cgi?targetID=1317252) | 103 | 82 | hsa-miR-29b-1-5p | [CECR6](http://www.ncbi.nlm.nih.gov/entrez/query.fcgi?db=gene&cmd=Retrieve&dopt=full_report&list_uids=27439) | cat eye syndrome chromosome region, candidate 6 |
| [Details](http://mirdb.org/cgi-bin/target_detail.cgi?targetID=1316961) | 104 | 82 | hsa-miR-29b-1-5p | [MED14](http://www.ncbi.nlm.nih.gov/entrez/query.fcgi?db=gene&cmd=Retrieve&dopt=full_report&list_uids=9282) | mediator complex subunit 14 |
| [Details](http://mirdb.org/cgi-bin/target_detail.cgi?targetID=1317090) | 105 | 82 | hsa-miR-29b-1-5p | [PDYN](http://www.ncbi.nlm.nih.gov/entrez/query.fcgi?db=gene&cmd=Retrieve&dopt=full_report&list_uids=5173) | prodynorphin |
| [Details](http://mirdb.org/cgi-bin/target_detail.cgi?targetID=1317092) | 106 | 82 | hsa-miR-29b-1-5p | [SEPT2](http://www.ncbi.nlm.nih.gov/entrez/query.fcgi?db=gene&cmd=Retrieve&dopt=full_report&list_uids=4735) | septin 2 |
| [Details](http://mirdb.org/cgi-bin/target_detail.cgi?targetID=1317112) | 107 | 82 | hsa-miR-29b-1-5p | [DLAT](http://www.ncbi.nlm.nih.gov/entrez/query.fcgi?db=gene&cmd=Retrieve&dopt=full_report&list_uids=1737) | dihydrolipoamide S-acetyltransferase |
| [Details](http://mirdb.org/cgi-bin/target_detail.cgi?targetID=1317011) | 108 | 82 | hsa-miR-29b-1-5p | [BNIP3L](http://www.ncbi.nlm.nih.gov/entrez/query.fcgi?db=gene&cmd=Retrieve&dopt=full_report&list_uids=665) | BCL2/adenovirus E1B 19kDa interacting protein 3-like |
| [Details](http://mirdb.org/cgi-bin/target_detail.cgi?targetID=1317153) | 109 | 82 | hsa-miR-29b-1-5p | [FRMD6](http://www.ncbi.nlm.nih.gov/entrez/query.fcgi?db=gene&cmd=Retrieve&dopt=full_report&list_uids=122786) | FERM domain containing 6 |
| [Details](http://mirdb.org/cgi-bin/target_detail.cgi?targetID=1317308) | 110 | 82 | hsa-miR-29b-1-5p | [PPP6R3](http://www.ncbi.nlm.nih.gov/entrez/query.fcgi?db=gene&cmd=Retrieve&dopt=full_report&list_uids=55291) | protein phosphatase 6, regulatory subunit 3 |
| [Details](http://mirdb.org/cgi-bin/target_detail.cgi?targetID=1317124) | 111 | 82 | hsa-miR-29b-1-5p | [TNPO1](http://www.ncbi.nlm.nih.gov/entrez/query.fcgi?db=gene&cmd=Retrieve&dopt=full_report&list_uids=3842) | transportin 1 |
| [Details](http://mirdb.org/cgi-bin/target_detail.cgi?targetID=1316989) | 112 | 82 | hsa-miR-29b-1-5p | [ITM2B](http://www.ncbi.nlm.nih.gov/entrez/query.fcgi?db=gene&cmd=Retrieve&dopt=full_report&list_uids=9445) | integral membrane protein 2B |
| [Details](http://mirdb.org/cgi-bin/target_detail.cgi?targetID=1317148) | 113 | 82 | hsa-miR-29b-1-5p | [AGAP3](http://www.ncbi.nlm.nih.gov/entrez/query.fcgi?db=gene&cmd=Retrieve&dopt=full_report&list_uids=116988) | ArfGAP with GTPase domain, ankyrin repeat and PH domain 3 |
| [Details](http://mirdb.org/cgi-bin/target_detail.cgi?targetID=1317083) | 114 | 81 | hsa-miR-29b-1-5p | [POLE3](http://www.ncbi.nlm.nih.gov/entrez/query.fcgi?db=gene&cmd=Retrieve&dopt=full_report&list_uids=54107) | polymerase (DNA directed), epsilon 3, accessory subunit |
| [Details](http://mirdb.org/cgi-bin/target_detail.cgi?targetID=1316978) | 115 | 81 | hsa-miR-29b-1-5p | [MAGI3](http://www.ncbi.nlm.nih.gov/entrez/query.fcgi?db=gene&cmd=Retrieve&dopt=full_report&list_uids=260425) | membrane associated guanylate kinase, WW and PDZ domain containing 3 |
| [Details](http://mirdb.org/cgi-bin/target_detail.cgi?targetID=1317048) | 116 | 81 | hsa-miR-29b-1-5p | [NUFIP1](http://www.ncbi.nlm.nih.gov/entrez/query.fcgi?db=gene&cmd=Retrieve&dopt=full_report&list_uids=26747) | nuclear fragile X mental retardation protein interacting protein 1 |
| [Details](http://mirdb.org/cgi-bin/target_detail.cgi?targetID=1316967) | 117 | 81 | hsa-miR-29b-1-5p | [BPNT1](http://www.ncbi.nlm.nih.gov/entrez/query.fcgi?db=gene&cmd=Retrieve&dopt=full_report&list_uids=10380) | 3'(2'), 5'-bisphosphate nucleotidase 1 |
| [Details](http://mirdb.org/cgi-bin/target_detail.cgi?targetID=1317249) | 118 | 80 | hsa-miR-29b-1-5p | [C8orf46](http://www.ncbi.nlm.nih.gov/entrez/query.fcgi?db=gene&cmd=Retrieve&dopt=full_report&list_uids=254778) | chromosome 8 open reading frame 46 |
| [Details](http://mirdb.org/cgi-bin/target_detail.cgi?targetID=1317307) | 119 | 80 | hsa-miR-29b-1-5p | [PATL1](http://www.ncbi.nlm.nih.gov/entrez/query.fcgi?db=gene&cmd=Retrieve&dopt=full_report&list_uids=219988) | protein associated with topoisomerase II homolog 1 (yeast) |
| [Details](http://mirdb.org/cgi-bin/target_detail.cgi?targetID=1317028) | 120 | 80 | hsa-miR-29b-1-5p | [RASSF8](http://www.ncbi.nlm.nih.gov/entrez/query.fcgi?db=gene&cmd=Retrieve&dopt=full_report&list_uids=11228) | Ras association (RalGDS/AF-6) domain family (N-terminal) member 8 |
| [Details](http://mirdb.org/cgi-bin/target_detail.cgi?targetID=1317344) | 121 | 80 | hsa-miR-29b-1-5p | [MIER3](http://www.ncbi.nlm.nih.gov/entrez/query.fcgi?db=gene&cmd=Retrieve&dopt=full_report&list_uids=166968) | mesoderm induction early response 1, family member 3 |
| [Details](http://mirdb.org/cgi-bin/target_detail.cgi?targetID=1317292) | 122 | 80 | hsa-miR-29b-1-5p | [KNSTRN](http://www.ncbi.nlm.nih.gov/entrez/query.fcgi?db=gene&cmd=Retrieve&dopt=full_report&list_uids=90417) | kinetochore-localized astrin/SPAG5 binding protein |
| [Details](http://mirdb.org/cgi-bin/target_detail.cgi?targetID=1317133) | 123 | 79 | hsa-miR-29b-1-5p | [CMIP](http://www.ncbi.nlm.nih.gov/entrez/query.fcgi?db=gene&cmd=Retrieve&dopt=full_report&list_uids=80790) | c-Maf inducing protein |
| [Details](http://mirdb.org/cgi-bin/target_detail.cgi?targetID=1317202) | 124 | 79 | hsa-miR-29b-1-5p | [EPS8L2](http://www.ncbi.nlm.nih.gov/entrez/query.fcgi?db=gene&cmd=Retrieve&dopt=full_report&list_uids=64787) | EPS8-like 2 |
| [Details](http://mirdb.org/cgi-bin/target_detail.cgi?targetID=1317257) | 125 | 79 | hsa-miR-29b-1-5p | [DACT1](http://www.ncbi.nlm.nih.gov/entrez/query.fcgi?db=gene&cmd=Retrieve&dopt=full_report&list_uids=51339) | dishevelled-binding antagonist of beta-catenin 1 |
| [Details](http://mirdb.org/cgi-bin/target_detail.cgi?targetID=1317139) | 126 | 78 | hsa-miR-29b-1-5p | [FEZF2](http://www.ncbi.nlm.nih.gov/entrez/query.fcgi?db=gene&cmd=Retrieve&dopt=full_report&list_uids=55079) | FEZ family zinc finger 2 |
| [Details](http://mirdb.org/cgi-bin/target_detail.cgi?targetID=1317324) | 127 | 78 | hsa-miR-29b-1-5p | [SRGAP2](http://www.ncbi.nlm.nih.gov/entrez/query.fcgi?db=gene&cmd=Retrieve&dopt=full_report&list_uids=23380) | SLIT-ROBO Rho GTPase activating protein 2 |
| [Details](http://mirdb.org/cgi-bin/target_detail.cgi?targetID=1317225) | 128 | 78 | hsa-miR-29b-1-5p | [VSIG1](http://www.ncbi.nlm.nih.gov/entrez/query.fcgi?db=gene&cmd=Retrieve&dopt=full_report&list_uids=340547) | V-set and immunoglobulin domain containing 1 |
| [Details](http://mirdb.org/cgi-bin/target_detail.cgi?targetID=1317068) | 129 | 78 | hsa-miR-29b-1-5p | [PLCL1](http://www.ncbi.nlm.nih.gov/entrez/query.fcgi?db=gene&cmd=Retrieve&dopt=full_report&list_uids=5334) | phospholipase C-like 1 |
| [Details](http://mirdb.org/cgi-bin/target_detail.cgi?targetID=1317272) | 130 | 78 | hsa-miR-29b-1-5p | [FGB](http://www.ncbi.nlm.nih.gov/entrez/query.fcgi?db=gene&cmd=Retrieve&dopt=full_report&list_uids=2244) | fibrinogen beta chain |
| [Details](http://mirdb.org/cgi-bin/target_detail.cgi?targetID=1317016) | 131 | 78 | hsa-miR-29b-1-5p | [AQP4](http://www.ncbi.nlm.nih.gov/entrez/query.fcgi?db=gene&cmd=Retrieve&dopt=full_report&list_uids=361) | aquaporin 4 |
| [Details](http://mirdb.org/cgi-bin/target_detail.cgi?targetID=1317015) | 132 | 78 | hsa-miR-29b-1-5p | [WDR48](http://www.ncbi.nlm.nih.gov/entrez/query.fcgi?db=gene&cmd=Retrieve&dopt=full_report&list_uids=57599) | WD repeat domain 48 |
| [Details](http://mirdb.org/cgi-bin/target_detail.cgi?targetID=1316956) | 133 | 78 | hsa-miR-29b-1-5p | [DPP8](http://www.ncbi.nlm.nih.gov/entrez/query.fcgi?db=gene&cmd=Retrieve&dopt=full_report&list_uids=54878) | dipeptidyl-peptidase 8 |
| [Details](http://mirdb.org/cgi-bin/target_detail.cgi?targetID=1317316) | 134 | 78 | hsa-miR-29b-1-5p | [CNRIP1](http://www.ncbi.nlm.nih.gov/entrez/query.fcgi?db=gene&cmd=Retrieve&dopt=full_report&list_uids=25927) | cannabinoid receptor interacting protein 1 |
| [Details](http://mirdb.org/cgi-bin/target_detail.cgi?targetID=1317160) | 135 | 77 | hsa-miR-29b-1-5p | [GABPA](http://www.ncbi.nlm.nih.gov/entrez/query.fcgi?db=gene&cmd=Retrieve&dopt=full_report&list_uids=2551) | GA binding protein transcription factor, alpha subunit 60kDa |
| [Details](http://mirdb.org/cgi-bin/target_detail.cgi?targetID=1317289) | 136 | 77 | hsa-miR-29b-1-5p | [SEZ6L2](http://www.ncbi.nlm.nih.gov/entrez/query.fcgi?db=gene&cmd=Retrieve&dopt=full_report&list_uids=26470) | seizure related 6 homolog (mouse)-like 2 |
| [Details](http://mirdb.org/cgi-bin/target_detail.cgi?targetID=1317141) | 137 | 77 | hsa-miR-29b-1-5p | [RGS22](http://www.ncbi.nlm.nih.gov/entrez/query.fcgi?db=gene&cmd=Retrieve&dopt=full_report&list_uids=26166) | regulator of G-protein signaling 22 |
| [Details](http://mirdb.org/cgi-bin/target_detail.cgi?targetID=1317176) | 138 | 77 | hsa-miR-29b-1-5p | [ABHD2](http://www.ncbi.nlm.nih.gov/entrez/query.fcgi?db=gene&cmd=Retrieve&dopt=full_report&list_uids=11057) | abhydrolase domain containing 2 |
| [Details](http://mirdb.org/cgi-bin/target_detail.cgi?targetID=1317331) | 139 | 76 | hsa-miR-29b-1-5p | [PTGFRN](http://www.ncbi.nlm.nih.gov/entrez/query.fcgi?db=gene&cmd=Retrieve&dopt=full_report&list_uids=5738) | prostaglandin F2 receptor inhibitor |
| [Details](http://mirdb.org/cgi-bin/target_detail.cgi?targetID=1317021) | 140 | 76 | hsa-miR-29b-1-5p | [DDIT4L](http://www.ncbi.nlm.nih.gov/entrez/query.fcgi?db=gene&cmd=Retrieve&dopt=full_report&list_uids=115265) | DNA-damage-inducible transcript 4-like |
| [Details](http://mirdb.org/cgi-bin/target_detail.cgi?targetID=1317213) | 141 | 76 | hsa-miR-29b-1-5p | [KAT6A](http://www.ncbi.nlm.nih.gov/entrez/query.fcgi?db=gene&cmd=Retrieve&dopt=full_report&list_uids=7994) | K(lysine) acetyltransferase 6A |
| [Details](http://mirdb.org/cgi-bin/target_detail.cgi?targetID=1317191) | 142 | 76 | hsa-miR-29b-1-5p | [BRD2](http://www.ncbi.nlm.nih.gov/entrez/query.fcgi?db=gene&cmd=Retrieve&dopt=full_report&list_uids=6046) | bromodomain containing 2 |
| [Details](http://mirdb.org/cgi-bin/target_detail.cgi?targetID=1317138) | 143 | 76 | hsa-miR-29b-1-5p | [PHLPP1](http://www.ncbi.nlm.nih.gov/entrez/query.fcgi?db=gene&cmd=Retrieve&dopt=full_report&list_uids=23239) | PH domain and leucine rich repeat protein phosphatase 1 |
| [Details](http://mirdb.org/cgi-bin/target_detail.cgi?targetID=1317222) | 144 | 76 | hsa-miR-29b-1-5p | [CHSY1](http://www.ncbi.nlm.nih.gov/entrez/query.fcgi?db=gene&cmd=Retrieve&dopt=full_report&list_uids=22856) | chondroitin sulfate synthase 1 |
| [Details](http://mirdb.org/cgi-bin/target_detail.cgi?targetID=1317199) | 145 | 75 | hsa-miR-29b-1-5p | [NPR2](http://www.ncbi.nlm.nih.gov/entrez/query.fcgi?db=gene&cmd=Retrieve&dopt=full_report&list_uids=4882) | natriuretic peptide receptor 2 |
| [Details](http://mirdb.org/cgi-bin/target_detail.cgi?targetID=1317003) | 146 | 75 | hsa-miR-29b-1-5p | [NOTCH2NL](http://www.ncbi.nlm.nih.gov/entrez/query.fcgi?db=gene&cmd=Retrieve&dopt=full_report&list_uids=388677) | notch 2 N-terminal like |
| [Details](http://mirdb.org/cgi-bin/target_detail.cgi?targetID=1317233) | 147 | 75 | hsa-miR-29b-1-5p | [ATG13](http://www.ncbi.nlm.nih.gov/entrez/query.fcgi?db=gene&cmd=Retrieve&dopt=full_report&list_uids=9776) | autophagy related 13 |
| [Details](http://mirdb.org/cgi-bin/target_detail.cgi?targetID=1317123) | 148 | 75 | hsa-miR-29b-1-5p | [WFDC10B](http://www.ncbi.nlm.nih.gov/entrez/query.fcgi?db=gene&cmd=Retrieve&dopt=full_report&list_uids=280664) | WAP four-disulfide core domain 10B |
| [Details](http://mirdb.org/cgi-bin/target_detail.cgi?targetID=1317325) | 149 | 75 | hsa-miR-29b-1-5p | [KAT6B](http://www.ncbi.nlm.nih.gov/entrez/query.fcgi?db=gene&cmd=Retrieve&dopt=full_report&list_uids=23522) | K(lysine) acetyltransferase 6B |
| [Details](http://mirdb.org/cgi-bin/target_detail.cgi?targetID=1317058) | 150 | 75 | hsa-miR-29b-1-5p | [TSPAN32](http://www.ncbi.nlm.nih.gov/entrez/query.fcgi?db=gene&cmd=Retrieve&dopt=full_report&list_uids=10077) | tetraspanin 32 |
| [Details](http://mirdb.org/cgi-bin/target_detail.cgi?targetID=1317095) | 151 | 75 | hsa-miR-29b-1-5p | [WFDC10A](http://www.ncbi.nlm.nih.gov/entrez/query.fcgi?db=gene&cmd=Retrieve&dopt=full_report&list_uids=140832) | WAP four-disulfide core domain 10A |
| [Details](http://mirdb.org/cgi-bin/target_detail.cgi?targetID=1317084) | 152 | 75 | hsa-miR-29b-1-5p | [PTPRK](http://www.ncbi.nlm.nih.gov/entrez/query.fcgi?db=gene&cmd=Retrieve&dopt=full_report&list_uids=5796) | protein tyrosine phosphatase, receptor type, K |
| [Details](http://mirdb.org/cgi-bin/target_detail.cgi?targetID=1317285) | 153 | 74 | hsa-miR-29b-1-5p | [ST8SIA5](http://www.ncbi.nlm.nih.gov/entrez/query.fcgi?db=gene&cmd=Retrieve&dopt=full_report&list_uids=29906) | ST8 alpha-N-acetyl-neuraminide alpha-2,8-sialyltransferase 5 |
| [Details](http://mirdb.org/cgi-bin/target_detail.cgi?targetID=1317340) | 154 | 74 | hsa-miR-29b-1-5p | [PARP2](http://www.ncbi.nlm.nih.gov/entrez/query.fcgi?db=gene&cmd=Retrieve&dopt=full_report&list_uids=10038) | poly (ADP-ribose) polymerase 2 |
| [Details](http://mirdb.org/cgi-bin/target_detail.cgi?targetID=1317322) | 155 | 74 | hsa-miR-29b-1-5p | [ENPP2](http://www.ncbi.nlm.nih.gov/entrez/query.fcgi?db=gene&cmd=Retrieve&dopt=full_report&list_uids=5168) | ectonucleotide pyrophosphatase/phosphodiesterase 2 |
| [Details](http://mirdb.org/cgi-bin/target_detail.cgi?targetID=1316997) | 156 | 74 | hsa-miR-29b-1-5p | [HEY1](http://www.ncbi.nlm.nih.gov/entrez/query.fcgi?db=gene&cmd=Retrieve&dopt=full_report&list_uids=23462) | hes-related family bHLH transcription factor with YRPW motif 1 |
| [Details](http://mirdb.org/cgi-bin/target_detail.cgi?targetID=1317214) | 157 | 74 | hsa-miR-29b-1-5p | [NAA25](http://www.ncbi.nlm.nih.gov/entrez/query.fcgi?db=gene&cmd=Retrieve&dopt=full_report&list_uids=80018) | N(alpha)-acetyltransferase 25, NatB auxiliary subunit |
| [Details](http://mirdb.org/cgi-bin/target_detail.cgi?targetID=1317135) | 158 | 73 | hsa-miR-29b-1-5p | [ABHD5](http://www.ncbi.nlm.nih.gov/entrez/query.fcgi?db=gene&cmd=Retrieve&dopt=full_report&list_uids=51099) | abhydrolase domain containing 5 |
| [Details](http://mirdb.org/cgi-bin/target_detail.cgi?targetID=1317154) | 159 | 73 | hsa-miR-29b-1-5p | [PCDHB13](http://www.ncbi.nlm.nih.gov/entrez/query.fcgi?db=gene&cmd=Retrieve&dopt=full_report&list_uids=56123) | protocadherin beta 13 |
| [Details](http://mirdb.org/cgi-bin/target_detail.cgi?targetID=1317057) | 160 | 73 | hsa-miR-29b-1-5p | [PLEKHM3](http://www.ncbi.nlm.nih.gov/entrez/query.fcgi?db=gene&cmd=Retrieve&dopt=full_report&list_uids=389072) | pleckstrin homology domain containing, family M, member 3 |
| [Details](http://mirdb.org/cgi-bin/target_detail.cgi?targetID=1316965) | 161 | 73 | hsa-miR-29b-1-5p | [FIGN](http://www.ncbi.nlm.nih.gov/entrez/query.fcgi?db=gene&cmd=Retrieve&dopt=full_report&list_uids=55137) | fidgetin |
| [Details](http://mirdb.org/cgi-bin/target_detail.cgi?targetID=1317050) | 162 | 73 | hsa-miR-29b-1-5p | [DCUN1D4](http://www.ncbi.nlm.nih.gov/entrez/query.fcgi?db=gene&cmd=Retrieve&dopt=full_report&list_uids=23142) | DCN1, defective in cullin neddylation 1, domain containing 4 |
| [Details](http://mirdb.org/cgi-bin/target_detail.cgi?targetID=1317074) | 163 | 73 | hsa-miR-29b-1-5p | [DPH2](http://www.ncbi.nlm.nih.gov/entrez/query.fcgi?db=gene&cmd=Retrieve&dopt=full_report&list_uids=1802) | DPH2 homolog (S. cerevisiae) |
| [Details](http://mirdb.org/cgi-bin/target_detail.cgi?targetID=1317299) | 164 | 73 | hsa-miR-29b-1-5p | [ZNF654](http://www.ncbi.nlm.nih.gov/entrez/query.fcgi?db=gene&cmd=Retrieve&dopt=full_report&list_uids=55279) | zinc finger protein 654 |
| [Details](http://mirdb.org/cgi-bin/target_detail.cgi?targetID=1317229) | 165 | 72 | hsa-miR-29b-1-5p | [SAA2](http://www.ncbi.nlm.nih.gov/entrez/query.fcgi?db=gene&cmd=Retrieve&dopt=full_report&list_uids=6289) | serum amyloid A2 |
| [Details](http://mirdb.org/cgi-bin/target_detail.cgi?targetID=1317044) | 166 | 72 | hsa-miR-29b-1-5p | [SNX30](http://www.ncbi.nlm.nih.gov/entrez/query.fcgi?db=gene&cmd=Retrieve&dopt=full_report&list_uids=401548) | sorting nexin family member 30 |
| [Details](http://mirdb.org/cgi-bin/target_detail.cgi?targetID=1317256) | 167 | 72 | hsa-miR-29b-1-5p | [RNF150](http://www.ncbi.nlm.nih.gov/entrez/query.fcgi?db=gene&cmd=Retrieve&dopt=full_report&list_uids=57484) | ring finger protein 150 |
| [Details](http://mirdb.org/cgi-bin/target_detail.cgi?targetID=1316968) | 168 | 72 | hsa-miR-29b-1-5p | [RUFY3](http://www.ncbi.nlm.nih.gov/entrez/query.fcgi?db=gene&cmd=Retrieve&dopt=full_report&list_uids=22902) | RUN and FYVE domain containing 3 |
| [Details](http://mirdb.org/cgi-bin/target_detail.cgi?targetID=1316993) | 169 | 72 | hsa-miR-29b-1-5p | [ZNF652](http://www.ncbi.nlm.nih.gov/entrez/query.fcgi?db=gene&cmd=Retrieve&dopt=full_report&list_uids=22834) | zinc finger protein 652 |
| [Details](http://mirdb.org/cgi-bin/target_detail.cgi?targetID=1316984) | 170 | 71 | hsa-miR-29b-1-5p | [ZKSCAN8](http://www.ncbi.nlm.nih.gov/entrez/query.fcgi?db=gene&cmd=Retrieve&dopt=full_report&list_uids=7745) | zinc finger with KRAB and SCAN domains 8 |
| [Details](http://mirdb.org/cgi-bin/target_detail.cgi?targetID=1316988) | 171 | 71 | hsa-miR-29b-1-5p | [PBRM1](http://www.ncbi.nlm.nih.gov/entrez/query.fcgi?db=gene&cmd=Retrieve&dopt=full_report&list_uids=55193) | polybromo 1 |
| [Details](http://mirdb.org/cgi-bin/target_detail.cgi?targetID=1316976) | 172 | 71 | hsa-miR-29b-1-5p | [KMT2D](http://www.ncbi.nlm.nih.gov/entrez/query.fcgi?db=gene&cmd=Retrieve&dopt=full_report&list_uids=8085) | lysine (K)-specific methyltransferase 2D |
| [Details](http://mirdb.org/cgi-bin/target_detail.cgi?targetID=1317025) | 173 | 71 | hsa-miR-29b-1-5p | [HNRNPC](http://www.ncbi.nlm.nih.gov/entrez/query.fcgi?db=gene&cmd=Retrieve&dopt=full_report&list_uids=3183) | heterogeneous nuclear ribonucleoprotein C (C1/C2) |
| [Details](http://mirdb.org/cgi-bin/target_detail.cgi?targetID=1317165) | 174 | 71 | hsa-miR-29b-1-5p | [RPS6KB1](http://www.ncbi.nlm.nih.gov/entrez/query.fcgi?db=gene&cmd=Retrieve&dopt=full_report&list_uids=6198) | ribosomal protein S6 kinase, 70kDa, polypeptide 1 |
| [Details](http://mirdb.org/cgi-bin/target_detail.cgi?targetID=1317109) | 175 | 71 | hsa-miR-29b-1-5p | [SMDT1](http://www.ncbi.nlm.nih.gov/entrez/query.fcgi?db=gene&cmd=Retrieve&dopt=full_report&list_uids=91689) | single-pass membrane protein with aspartate-rich tail 1 |
| [Details](http://mirdb.org/cgi-bin/target_detail.cgi?targetID=1317071) | 176 | 71 | hsa-miR-29b-1-5p | [MAP3K1](http://www.ncbi.nlm.nih.gov/entrez/query.fcgi?db=gene&cmd=Retrieve&dopt=full_report&list_uids=4214) | mitogen-activated protein kinase kinase kinase 1, E3 ubiquitin protein ligase |
| [Details](http://mirdb.org/cgi-bin/target_detail.cgi?targetID=1317227) | 177 | 70 | hsa-miR-29b-1-5p | [ABCB5](http://www.ncbi.nlm.nih.gov/entrez/query.fcgi?db=gene&cmd=Retrieve&dopt=full_report&list_uids=340273) | ATP-binding cassette, sub-family B (MDR/TAP), member 5 |
| [Details](http://mirdb.org/cgi-bin/target_detail.cgi?targetID=1317240) | 178 | 70 | hsa-miR-29b-1-5p | [ALDH1A3](http://www.ncbi.nlm.nih.gov/entrez/query.fcgi?db=gene&cmd=Retrieve&dopt=full_report&list_uids=220) | aldehyde dehydrogenase 1 family, member A3 |
| [Details](http://mirdb.org/cgi-bin/target_detail.cgi?targetID=1317116) | 179 | 70 | hsa-miR-29b-1-5p | [ASF1A](http://www.ncbi.nlm.nih.gov/entrez/query.fcgi?db=gene&cmd=Retrieve&dopt=full_report&list_uids=25842) | anti-silencing function 1A histone chaperone |
| [Details](http://mirdb.org/cgi-bin/target_detail.cgi?targetID=1317338) | 180 | 70 | hsa-miR-29b-1-5p | [POU2F2](http://www.ncbi.nlm.nih.gov/entrez/query.fcgi?db=gene&cmd=Retrieve&dopt=full_report&list_uids=5452) | POU class 2 homeobox 2 |
| [Details](http://mirdb.org/cgi-bin/target_detail.cgi?targetID=1317036) | 181 | 69 | hsa-miR-29b-1-5p | [TMEM216](http://www.ncbi.nlm.nih.gov/entrez/query.fcgi?db=gene&cmd=Retrieve&dopt=full_report&list_uids=51259) | transmembrane protein 216 |
| [Details](http://mirdb.org/cgi-bin/target_detail.cgi?targetID=1317172) | 182 | 69 | hsa-miR-29b-1-5p | [PELI1](http://www.ncbi.nlm.nih.gov/entrez/query.fcgi?db=gene&cmd=Retrieve&dopt=full_report&list_uids=57162) | pellino E3 ubiquitin protein ligase 1 |
| [Details](http://mirdb.org/cgi-bin/target_detail.cgi?targetID=1317237) | 183 | 69 | hsa-miR-29b-1-5p | [CALCR](http://www.ncbi.nlm.nih.gov/entrez/query.fcgi?db=gene&cmd=Retrieve&dopt=full_report&list_uids=799) | calcitonin receptor |
| [Details](http://mirdb.org/cgi-bin/target_detail.cgi?targetID=1317029) | 184 | 69 | hsa-miR-29b-1-5p | [NAV3](http://www.ncbi.nlm.nih.gov/entrez/query.fcgi?db=gene&cmd=Retrieve&dopt=full_report&list_uids=89795) | neuron navigator 3 |
| [Details](http://mirdb.org/cgi-bin/target_detail.cgi?targetID=1317183) | 185 | 69 | hsa-miR-29b-1-5p | [AGO2](http://www.ncbi.nlm.nih.gov/entrez/query.fcgi?db=gene&cmd=Retrieve&dopt=full_report&list_uids=27161) | argonaute RISC catalytic component 2 |
| [Details](http://mirdb.org/cgi-bin/target_detail.cgi?targetID=1317182) | 186 | 69 | hsa-miR-29b-1-5p | [SLC22A23](http://www.ncbi.nlm.nih.gov/entrez/query.fcgi?db=gene&cmd=Retrieve&dopt=full_report&list_uids=63027) | solute carrier family 22, member 23 |
| [Details](http://mirdb.org/cgi-bin/target_detail.cgi?targetID=1317303) | 187 | 69 | hsa-miR-29b-1-5p | [SLC25A4](http://www.ncbi.nlm.nih.gov/entrez/query.fcgi?db=gene&cmd=Retrieve&dopt=full_report&list_uids=291) | solute carrier family 25 (mitochondrial carrier; adenine nucleotide translocator), member 4 |
| [Details](http://mirdb.org/cgi-bin/target_detail.cgi?targetID=1317264) | 188 | 69 | hsa-miR-29b-1-5p | [CLOCK](http://www.ncbi.nlm.nih.gov/entrez/query.fcgi?db=gene&cmd=Retrieve&dopt=full_report&list_uids=9575) | clock circadian regulator |
| [Details](http://mirdb.org/cgi-bin/target_detail.cgi?targetID=1317008) | 189 | 69 | hsa-miR-29b-1-5p | [RNF41](http://www.ncbi.nlm.nih.gov/entrez/query.fcgi?db=gene&cmd=Retrieve&dopt=full_report&list_uids=10193) | ring finger protein 41, E3 ubiquitin protein ligase |
| [Details](http://mirdb.org/cgi-bin/target_detail.cgi?targetID=1317162) | 190 | 69 | hsa-miR-29b-1-5p | [RBM18](http://www.ncbi.nlm.nih.gov/entrez/query.fcgi?db=gene&cmd=Retrieve&dopt=full_report&list_uids=92400) | RNA binding motif protein 18 |
| [Details](http://mirdb.org/cgi-bin/target_detail.cgi?targetID=1316958) | 191 | 68 | hsa-miR-29b-1-5p | [CCDC117](http://www.ncbi.nlm.nih.gov/entrez/query.fcgi?db=gene&cmd=Retrieve&dopt=full_report&list_uids=150275) | coiled-coil domain containing 117 |
| [Details](http://mirdb.org/cgi-bin/target_detail.cgi?targetID=1317336) | 192 | 68 | hsa-miR-29b-1-5p | [FCHO2](http://www.ncbi.nlm.nih.gov/entrez/query.fcgi?db=gene&cmd=Retrieve&dopt=full_report&list_uids=115548) | FCH domain only 2 |
| [Details](http://mirdb.org/cgi-bin/target_detail.cgi?targetID=1317198) | 193 | 68 | hsa-miR-29b-1-5p | [ARHGAP25](http://www.ncbi.nlm.nih.gov/entrez/query.fcgi?db=gene&cmd=Retrieve&dopt=full_report&list_uids=9938) | Rho GTPase activating protein 25 |
| [Details](http://mirdb.org/cgi-bin/target_detail.cgi?targetID=1317337) | 194 | 68 | hsa-miR-29b-1-5p | [ANKRD28](http://www.ncbi.nlm.nih.gov/entrez/query.fcgi?db=gene&cmd=Retrieve&dopt=full_report&list_uids=23243) | ankyrin repeat domain 28 |
| [Details](http://mirdb.org/cgi-bin/target_detail.cgi?targetID=1317076) | 195 | 68 | hsa-miR-29b-1-5p | [NR1D2](http://www.ncbi.nlm.nih.gov/entrez/query.fcgi?db=gene&cmd=Retrieve&dopt=full_report&list_uids=9975) | nuclear receptor subfamily 1, group D, member 2 |
| [Details](http://mirdb.org/cgi-bin/target_detail.cgi?targetID=1317224) | 196 | 68 | hsa-miR-29b-1-5p | [ZC3H12D](http://www.ncbi.nlm.nih.gov/entrez/query.fcgi?db=gene&cmd=Retrieve&dopt=full_report&list_uids=340152) | zinc finger CCCH-type containing 12D |
| [Details](http://mirdb.org/cgi-bin/target_detail.cgi?targetID=1317110) | 197 | 68 | hsa-miR-29b-1-5p | [EIF4EBP2](http://www.ncbi.nlm.nih.gov/entrez/query.fcgi?db=gene&cmd=Retrieve&dopt=full_report&list_uids=1979) | eukaryotic translation initiation factor 4E binding protein 2 |
| [Details](http://mirdb.org/cgi-bin/target_detail.cgi?targetID=1317023) | 198 | 67 | hsa-miR-29b-1-5p | [CCNA2](http://www.ncbi.nlm.nih.gov/entrez/query.fcgi?db=gene&cmd=Retrieve&dopt=full_report&list_uids=890) | cyclin A2 |
| [Details](http://mirdb.org/cgi-bin/target_detail.cgi?targetID=1317030) | 199 | 67 | hsa-miR-29b-1-5p | [PDGFC](http://www.ncbi.nlm.nih.gov/entrez/query.fcgi?db=gene&cmd=Retrieve&dopt=full_report&list_uids=56034) | platelet derived growth factor C |
| [Details](http://mirdb.org/cgi-bin/target_detail.cgi?targetID=1317246) | 200 | 67 | hsa-miR-29b-1-5p | [ARHGAP28](http://www.ncbi.nlm.nih.gov/entrez/query.fcgi?db=gene&cmd=Retrieve&dopt=full_report&list_uids=79822) | Rho GTPase activating protein 28 |
| [Details](http://mirdb.org/cgi-bin/target_detail.cgi?targetID=1317082) | 201 | 67 | hsa-miR-29b-1-5p | [PDE6A](http://www.ncbi.nlm.nih.gov/entrez/query.fcgi?db=gene&cmd=Retrieve&dopt=full_report&list_uids=5145) | phosphodiesterase 6A, cGMP-specific, rod, alpha |
| [Details](http://mirdb.org/cgi-bin/target_detail.cgi?targetID=1317171) | 202 | 67 | hsa-miR-29b-1-5p | [IL6ST](http://www.ncbi.nlm.nih.gov/entrez/query.fcgi?db=gene&cmd=Retrieve&dopt=full_report&list_uids=3572) | interleukin 6 signal transducer (gp130, oncostatin M receptor) |
| [Details](http://mirdb.org/cgi-bin/target_detail.cgi?targetID=1317046) | 203 | 67 | hsa-miR-29b-1-5p | [ZNF687](http://www.ncbi.nlm.nih.gov/entrez/query.fcgi?db=gene&cmd=Retrieve&dopt=full_report&list_uids=57592) | zinc finger protein 687 |
| [Details](http://mirdb.org/cgi-bin/target_detail.cgi?targetID=1317040) | 204 | 67 | hsa-miR-29b-1-5p | [CRIPT](http://www.ncbi.nlm.nih.gov/entrez/query.fcgi?db=gene&cmd=Retrieve&dopt=full_report&list_uids=9419) | cysteine-rich PDZ-binding protein |
| [Details](http://mirdb.org/cgi-bin/target_detail.cgi?targetID=1317346) | 205 | 67 | hsa-miR-29b-1-5p | [KIAA0513](http://www.ncbi.nlm.nih.gov/entrez/query.fcgi?db=gene&cmd=Retrieve&dopt=full_report&list_uids=9764) | KIAA0513 |
| [Details](http://mirdb.org/cgi-bin/target_detail.cgi?targetID=1317130) | 206 | 67 | hsa-miR-29b-1-5p | [IKZF2](http://www.ncbi.nlm.nih.gov/entrez/query.fcgi?db=gene&cmd=Retrieve&dopt=full_report&list_uids=22807) | IKAROS family zinc finger 2 (Helios) |
| [Details](http://mirdb.org/cgi-bin/target_detail.cgi?targetID=1317315) | 207 | 67 | hsa-miR-29b-1-5p | [ANKRD49](http://www.ncbi.nlm.nih.gov/entrez/query.fcgi?db=gene&cmd=Retrieve&dopt=full_report&list_uids=54851) | ankyrin repeat domain 49 |
| [Details](http://mirdb.org/cgi-bin/target_detail.cgi?targetID=1317206) | 208 | 67 | hsa-miR-29b-1-5p | [AREL1](http://www.ncbi.nlm.nih.gov/entrez/query.fcgi?db=gene&cmd=Retrieve&dopt=full_report&list_uids=9870) | apoptosis resistant E3 ubiquitin protein ligase 1 |
| [Details](http://mirdb.org/cgi-bin/target_detail.cgi?targetID=1317005) | 209 | 67 | hsa-miR-29b-1-5p | [PNISR](http://www.ncbi.nlm.nih.gov/entrez/query.fcgi?db=gene&cmd=Retrieve&dopt=full_report&list_uids=25957) | PNN-interacting serine/arginine-rich protein |
| [Details](http://mirdb.org/cgi-bin/target_detail.cgi?targetID=1317131) | 210 | 66 | hsa-miR-29b-1-5p | [RNF103](http://www.ncbi.nlm.nih.gov/entrez/query.fcgi?db=gene&cmd=Retrieve&dopt=full_report&list_uids=7844) | ring finger protein 103 |
| [Details](http://mirdb.org/cgi-bin/target_detail.cgi?targetID=1317185) | 211 | 66 | hsa-miR-29b-1-5p | [ILDR2](http://www.ncbi.nlm.nih.gov/entrez/query.fcgi?db=gene&cmd=Retrieve&dopt=full_report&list_uids=387597) | immunoglobulin-like domain containing receptor 2 |
| [Details](http://mirdb.org/cgi-bin/target_detail.cgi?targetID=1317072) | 212 | 66 | hsa-miR-29b-1-5p | [POU6F1](http://www.ncbi.nlm.nih.gov/entrez/query.fcgi?db=gene&cmd=Retrieve&dopt=full_report&list_uids=5463) | POU class 6 homeobox 1 |
| [Details](http://mirdb.org/cgi-bin/target_detail.cgi?targetID=1317144) | 213 | 66 | hsa-miR-29b-1-5p | [WWC1](http://www.ncbi.nlm.nih.gov/entrez/query.fcgi?db=gene&cmd=Retrieve&dopt=full_report&list_uids=23286) | WW and C2 domain containing 1 |
| [Details](http://mirdb.org/cgi-bin/target_detail.cgi?targetID=1316983) | 214 | 66 | hsa-miR-29b-1-5p | [PHC3](http://www.ncbi.nlm.nih.gov/entrez/query.fcgi?db=gene&cmd=Retrieve&dopt=full_report&list_uids=80012) | polyhomeotic homolog 3 (Drosophila) |
| [Details](http://mirdb.org/cgi-bin/target_detail.cgi?targetID=1317223) | 215 | 66 | hsa-miR-29b-1-5p | [PYURF](http://www.ncbi.nlm.nih.gov/entrez/query.fcgi?db=gene&cmd=Retrieve&dopt=full_report&list_uids=100996939) | PIGY upstream reading frame |
| [Details](http://mirdb.org/cgi-bin/target_detail.cgi?targetID=1317042) | 216 | 66 | hsa-miR-29b-1-5p | [QSER1](http://www.ncbi.nlm.nih.gov/entrez/query.fcgi?db=gene&cmd=Retrieve&dopt=full_report&list_uids=79832) | glutamine and serine rich 1 |
| [Details](http://mirdb.org/cgi-bin/target_detail.cgi?targetID=1317041) | 217 | 66 | hsa-miR-29b-1-5p | [MTMR9](http://www.ncbi.nlm.nih.gov/entrez/query.fcgi?db=gene&cmd=Retrieve&dopt=full_report&list_uids=66036) | myotubularin related protein 9 |
| [Details](http://mirdb.org/cgi-bin/target_detail.cgi?targetID=1316980) | 218 | 66 | hsa-miR-29b-1-5p | [NFAT5](http://www.ncbi.nlm.nih.gov/entrez/query.fcgi?db=gene&cmd=Retrieve&dopt=full_report&list_uids=10725) | nuclear factor of activated T-cells 5, tonicity-responsive |
| [Details](http://mirdb.org/cgi-bin/target_detail.cgi?targetID=1317184) | 219 | 65 | hsa-miR-29b-1-5p | [GPC4](http://www.ncbi.nlm.nih.gov/entrez/query.fcgi?db=gene&cmd=Retrieve&dopt=full_report&list_uids=2239) | glypican 4 |
| [Details](http://mirdb.org/cgi-bin/target_detail.cgi?targetID=1317161) | 220 | 65 | hsa-miR-29b-1-5p | [SHISA2](http://www.ncbi.nlm.nih.gov/entrez/query.fcgi?db=gene&cmd=Retrieve&dopt=full_report&list_uids=387914) | shisa family member 2 |
| [Details](http://mirdb.org/cgi-bin/target_detail.cgi?targetID=1317311) | 221 | 65 | hsa-miR-29b-1-5p | [DACH1](http://www.ncbi.nlm.nih.gov/entrez/query.fcgi?db=gene&cmd=Retrieve&dopt=full_report&list_uids=1602) | dachshund family transcription factor 1 |
| [Details](http://mirdb.org/cgi-bin/target_detail.cgi?targetID=1317251) | 222 | 65 | hsa-miR-29b-1-5p | [CAPSL](http://www.ncbi.nlm.nih.gov/entrez/query.fcgi?db=gene&cmd=Retrieve&dopt=full_report&list_uids=133690) | calcyphosine-like |
| [Details](http://mirdb.org/cgi-bin/target_detail.cgi?targetID=1317136) | 223 | 65 | hsa-miR-29b-1-5p | [IST1](http://www.ncbi.nlm.nih.gov/entrez/query.fcgi?db=gene&cmd=Retrieve&dopt=full_report&list_uids=9798) | increased sodium tolerance 1 homolog (yeast) |
| [Details](http://mirdb.org/cgi-bin/target_detail.cgi?targetID=1317328) | 224 | 64 | hsa-miR-29b-1-5p | [ST13](http://www.ncbi.nlm.nih.gov/entrez/query.fcgi?db=gene&cmd=Retrieve&dopt=full_report&list_uids=6767) | suppression of tumorigenicity 13 (colon carcinoma) (Hsp70 interacting protein) |
| [Details](http://mirdb.org/cgi-bin/target_detail.cgi?targetID=1317277) | 225 | 64 | hsa-miR-29b-1-5p | [STX16](http://www.ncbi.nlm.nih.gov/entrez/query.fcgi?db=gene&cmd=Retrieve&dopt=full_report&list_uids=8675) | syntaxin 16 |
| [Details](http://mirdb.org/cgi-bin/target_detail.cgi?targetID=1317200) | 226 | 64 | hsa-miR-29b-1-5p | [GBP5](http://www.ncbi.nlm.nih.gov/entrez/query.fcgi?db=gene&cmd=Retrieve&dopt=full_report&list_uids=115362) | guanylate binding protein 5 |
| [Details](http://mirdb.org/cgi-bin/target_detail.cgi?targetID=1317259) | 227 | 64 | hsa-miR-29b-1-5p | [REST](http://www.ncbi.nlm.nih.gov/entrez/query.fcgi?db=gene&cmd=Retrieve&dopt=full_report&list_uids=5978) | RE1-silencing transcription factor |
| [Details](http://mirdb.org/cgi-bin/target_detail.cgi?targetID=1317218) | 228 | 64 | hsa-miR-29b-1-5p | [ZC3H8](http://www.ncbi.nlm.nih.gov/entrez/query.fcgi?db=gene&cmd=Retrieve&dopt=full_report&list_uids=84524) | zinc finger CCCH-type containing 8 |
| [Details](http://mirdb.org/cgi-bin/target_detail.cgi?targetID=1317297) | 229 | 64 | hsa-miR-29b-1-5p | [SLC1A4](http://www.ncbi.nlm.nih.gov/entrez/query.fcgi?db=gene&cmd=Retrieve&dopt=full_report&list_uids=6509) | solute carrier family 1 (glutamate/neutral amino acid transporter), member 4 |
| [Details](http://mirdb.org/cgi-bin/target_detail.cgi?targetID=1317064) | 230 | 64 | hsa-miR-29b-1-5p | [STAT4](http://www.ncbi.nlm.nih.gov/entrez/query.fcgi?db=gene&cmd=Retrieve&dopt=full_report&list_uids=6775) | signal transducer and activator of transcription 4 |
| [Details](http://mirdb.org/cgi-bin/target_detail.cgi?targetID=1317022) | 231 | 64 | hsa-miR-29b-1-5p | [FAM78A](http://www.ncbi.nlm.nih.gov/entrez/query.fcgi?db=gene&cmd=Retrieve&dopt=full_report&list_uids=286336) | family with sequence similarity 78, member A |
| [Details](http://mirdb.org/cgi-bin/target_detail.cgi?targetID=1316971) | 232 | 64 | hsa-miR-29b-1-5p | [SPTSSB](http://www.ncbi.nlm.nih.gov/entrez/query.fcgi?db=gene&cmd=Retrieve&dopt=full_report&list_uids=165679) | serine palmitoyltransferase, small subunit B |
| [Details](http://mirdb.org/cgi-bin/target_detail.cgi?targetID=1317140) | 233 | 64 | hsa-miR-29b-1-5p | [CMTM3](http://www.ncbi.nlm.nih.gov/entrez/query.fcgi?db=gene&cmd=Retrieve&dopt=full_report&list_uids=123920) | CKLF-like MARVEL transmembrane domain containing 3 |
| [Details](http://mirdb.org/cgi-bin/target_detail.cgi?targetID=1317164) | 234 | 64 | hsa-miR-29b-1-5p | [PIGY](http://www.ncbi.nlm.nih.gov/entrez/query.fcgi?db=gene&cmd=Retrieve&dopt=full_report&list_uids=84992) | phosphatidylinositol glycan anchor biosynthesis, class Y |
| [Details](http://mirdb.org/cgi-bin/target_detail.cgi?targetID=1317077) | 235 | 64 | hsa-miR-29b-1-5p | [AKT3](http://www.ncbi.nlm.nih.gov/entrez/query.fcgi?db=gene&cmd=Retrieve&dopt=full_report&list_uids=10000) | v-akt murine thymoma viral oncogene homolog 3 |
| [Details](http://mirdb.org/cgi-bin/target_detail.cgi?targetID=1317330) | 236 | 63 | hsa-miR-29b-1-5p | [PLXNC1](http://www.ncbi.nlm.nih.gov/entrez/query.fcgi?db=gene&cmd=Retrieve&dopt=full_report&list_uids=10154) | plexin C1 |
| [Details](http://mirdb.org/cgi-bin/target_detail.cgi?targetID=1317146) | 237 | 63 | hsa-miR-29b-1-5p | [PROM2](http://www.ncbi.nlm.nih.gov/entrez/query.fcgi?db=gene&cmd=Retrieve&dopt=full_report&list_uids=150696) | prominin 2 |
| [Details](http://mirdb.org/cgi-bin/target_detail.cgi?targetID=1317212) | 238 | 63 | hsa-miR-29b-1-5p | [ZC2HC1C](http://www.ncbi.nlm.nih.gov/entrez/query.fcgi?db=gene&cmd=Retrieve&dopt=full_report&list_uids=79696) | zinc finger, C2HC-type containing 1C |
| [Details](http://mirdb.org/cgi-bin/target_detail.cgi?targetID=1317334) | 239 | 63 | hsa-miR-29b-1-5p | [CA2](http://www.ncbi.nlm.nih.gov/entrez/query.fcgi?db=gene&cmd=Retrieve&dopt=full_report&list_uids=760) | carbonic anhydrase II |
| [Details](http://mirdb.org/cgi-bin/target_detail.cgi?targetID=1317216) | 240 | 63 | hsa-miR-29b-1-5p | [STK35](http://www.ncbi.nlm.nih.gov/entrez/query.fcgi?db=gene&cmd=Retrieve&dopt=full_report&list_uids=140901) | serine/threonine kinase 35 |
| [Details](http://mirdb.org/cgi-bin/target_detail.cgi?targetID=1317193) | 241 | 63 | hsa-miR-29b-1-5p | [ZBTB8A](http://www.ncbi.nlm.nih.gov/entrez/query.fcgi?db=gene&cmd=Retrieve&dopt=full_report&list_uids=653121) | zinc finger and BTB domain containing 8A |
| [Details](http://mirdb.org/cgi-bin/target_detail.cgi?targetID=1316996) | 242 | 63 | hsa-miR-29b-1-5p | [CCDC83](http://www.ncbi.nlm.nih.gov/entrez/query.fcgi?db=gene&cmd=Retrieve&dopt=full_report&list_uids=220047) | coiled-coil domain containing 83 |
| [Details](http://mirdb.org/cgi-bin/target_detail.cgi?targetID=1317238) | 243 | 63 | hsa-miR-29b-1-5p | [CEP41](http://www.ncbi.nlm.nih.gov/entrez/query.fcgi?db=gene&cmd=Retrieve&dopt=full_report&list_uids=95681) | centrosomal protein 41kDa |
| [Details](http://mirdb.org/cgi-bin/target_detail.cgi?targetID=1317262) | 244 | 63 | hsa-miR-29b-1-5p | [PERP](http://www.ncbi.nlm.nih.gov/entrez/query.fcgi?db=gene&cmd=Retrieve&dopt=full_report&list_uids=64065) | PERP, TP53 apoptosis effector |
| [Details](http://mirdb.org/cgi-bin/target_detail.cgi?targetID=1317054) | 245 | 63 | hsa-miR-29b-1-5p | [RNF11](http://www.ncbi.nlm.nih.gov/entrez/query.fcgi?db=gene&cmd=Retrieve&dopt=full_report&list_uids=26994) | ring finger protein 11 |
| [Details](http://mirdb.org/cgi-bin/target_detail.cgi?targetID=1317122) | 246 | 63 | hsa-miR-29b-1-5p | [ZCCHC14](http://www.ncbi.nlm.nih.gov/entrez/query.fcgi?db=gene&cmd=Retrieve&dopt=full_report&list_uids=23174) | zinc finger, CCHC domain containing 14 |
| [Details](http://mirdb.org/cgi-bin/target_detail.cgi?targetID=1317066) | 247 | 63 | hsa-miR-29b-1-5p | [GRSF1](http://www.ncbi.nlm.nih.gov/entrez/query.fcgi?db=gene&cmd=Retrieve&dopt=full_report&list_uids=2926) | G-rich RNA sequence binding factor 1 |
| [Details](http://mirdb.org/cgi-bin/target_detail.cgi?targetID=1317190) | 248 | 63 | hsa-miR-29b-1-5p | [CLCF1](http://www.ncbi.nlm.nih.gov/entrez/query.fcgi?db=gene&cmd=Retrieve&dopt=full_report&list_uids=23529) | cardiotrophin-like cytokine factor 1 |
| [Details](http://mirdb.org/cgi-bin/target_detail.cgi?targetID=1317128) | 249 | 63 | hsa-miR-29b-1-5p | [GTF3C5](http://www.ncbi.nlm.nih.gov/entrez/query.fcgi?db=gene&cmd=Retrieve&dopt=full_report&list_uids=9328) | general transcription factor IIIC, polypeptide 5, 63kDa |
| [Details](http://mirdb.org/cgi-bin/target_detail.cgi?targetID=1317120) | 250 | 62 | hsa-miR-29b-1-5p | [RASAL2](http://www.ncbi.nlm.nih.gov/entrez/query.fcgi?db=gene&cmd=Retrieve&dopt=full_report&list_uids=9462) | RAS protein activator like 2 |
| [Details](http://mirdb.org/cgi-bin/target_detail.cgi?targetID=1317215) | 251 | 62 | hsa-miR-29b-1-5p | [ARIH1](http://www.ncbi.nlm.nih.gov/entrez/query.fcgi?db=gene&cmd=Retrieve&dopt=full_report&list_uids=25820) | ariadne RBR E3 ubiquitin protein ligase 1 |
| [Details](http://mirdb.org/cgi-bin/target_detail.cgi?targetID=1317333) | 252 | 62 | hsa-miR-29b-1-5p | [TIA1](http://www.ncbi.nlm.nih.gov/entrez/query.fcgi?db=gene&cmd=Retrieve&dopt=full_report&list_uids=7072) | TIA1 cytotoxic granule-associated RNA binding protein |
| [Details](http://mirdb.org/cgi-bin/target_detail.cgi?targetID=1317049) | 253 | 62 | hsa-miR-29b-1-5p | [HIVEP1](http://www.ncbi.nlm.nih.gov/entrez/query.fcgi?db=gene&cmd=Retrieve&dopt=full_report&list_uids=3096) | human immunodeficiency virus type I enhancer binding protein 1 |
| [Details](http://mirdb.org/cgi-bin/target_detail.cgi?targetID=1317332) | 254 | 62 | hsa-miR-29b-1-5p | [TNRC6A](http://www.ncbi.nlm.nih.gov/entrez/query.fcgi?db=gene&cmd=Retrieve&dopt=full_report&list_uids=27327) | trinucleotide repeat containing 6A |
| [Details](http://mirdb.org/cgi-bin/target_detail.cgi?targetID=1316960) | 255 | 62 | hsa-miR-29b-1-5p | [IKZF5](http://www.ncbi.nlm.nih.gov/entrez/query.fcgi?db=gene&cmd=Retrieve&dopt=full_report&list_uids=64376) | IKAROS family zinc finger 5 (Pegasus) |
| [Details](http://mirdb.org/cgi-bin/target_detail.cgi?targetID=1317283) | 256 | 62 | hsa-miR-29b-1-5p | [SLC41A2](http://www.ncbi.nlm.nih.gov/entrez/query.fcgi?db=gene&cmd=Retrieve&dopt=full_report&list_uids=84102) | solute carrier family 41 (magnesium transporter), member 2 |
| [Details](http://mirdb.org/cgi-bin/target_detail.cgi?targetID=1317186) | 257 | 61 | hsa-miR-29b-1-5p | [DGKE](http://www.ncbi.nlm.nih.gov/entrez/query.fcgi?db=gene&cmd=Retrieve&dopt=full_report&list_uids=8526) | diacylglycerol kinase, epsilon 64kDa |
| [Details](http://mirdb.org/cgi-bin/target_detail.cgi?targetID=1317197) | 258 | 61 | hsa-miR-29b-1-5p | [SPRED1](http://www.ncbi.nlm.nih.gov/entrez/query.fcgi?db=gene&cmd=Retrieve&dopt=full_report&list_uids=161742) | sprouty-related, EVH1 domain containing 1 |
| [Details](http://mirdb.org/cgi-bin/target_detail.cgi?targetID=1317217) | 259 | 61 | hsa-miR-29b-1-5p | [NCALD](http://www.ncbi.nlm.nih.gov/entrez/query.fcgi?db=gene&cmd=Retrieve&dopt=full_report&list_uids=83988) | neurocalcin delta |
| [Details](http://mirdb.org/cgi-bin/target_detail.cgi?targetID=1317278) | 260 | 61 | hsa-miR-29b-1-5p | [APOBEC3F](http://www.ncbi.nlm.nih.gov/entrez/query.fcgi?db=gene&cmd=Retrieve&dopt=full_report&list_uids=200316) | apolipoprotein B mRNA editing enzyme, catalytic polypeptide-like 3F |
| [Details](http://mirdb.org/cgi-bin/target_detail.cgi?targetID=1317318) | 261 | 61 | hsa-miR-29b-1-5p | [COX11](http://www.ncbi.nlm.nih.gov/entrez/query.fcgi?db=gene&cmd=Retrieve&dopt=full_report&list_uids=1353) | cytochrome c oxidase assembly homolog 11 (yeast) |
| [Details](http://mirdb.org/cgi-bin/target_detail.cgi?targetID=1317004) | 262 | 61 | hsa-miR-29b-1-5p | [LRRTM2](http://www.ncbi.nlm.nih.gov/entrez/query.fcgi?db=gene&cmd=Retrieve&dopt=full_report&list_uids=26045) | leucine rich repeat transmembrane neuronal 2 |
| [Details](http://mirdb.org/cgi-bin/target_detail.cgi?targetID=1317151) | 263 | 61 | hsa-miR-29b-1-5p | [SRFBP1](http://www.ncbi.nlm.nih.gov/entrez/query.fcgi?db=gene&cmd=Retrieve&dopt=full_report&list_uids=153443) | serum response factor binding protein 1 |
| [Details](http://mirdb.org/cgi-bin/target_detail.cgi?targetID=1317301) | 264 | 61 | hsa-miR-29b-1-5p | [GUCA2A](http://www.ncbi.nlm.nih.gov/entrez/query.fcgi?db=gene&cmd=Retrieve&dopt=full_report&list_uids=2980) | guanylate cyclase activator 2A (guanylin) |
| [Details](http://mirdb.org/cgi-bin/target_detail.cgi?targetID=1316986) | 265 | 60 | hsa-miR-29b-1-5p | [NTN4](http://www.ncbi.nlm.nih.gov/entrez/query.fcgi?db=gene&cmd=Retrieve&dopt=full_report&list_uids=59277) | netrin 4 |
| [Details](http://mirdb.org/cgi-bin/target_detail.cgi?targetID=1317038) | 266 | 60 | hsa-miR-29b-1-5p | [SP6](http://www.ncbi.nlm.nih.gov/entrez/query.fcgi?db=gene&cmd=Retrieve&dopt=full_report&list_uids=80320) | Sp6 transcription factor |
| [Details](http://mirdb.org/cgi-bin/target_detail.cgi?targetID=1317231) | 267 | 60 | hsa-miR-29b-1-5p | [IGF2BP2](http://www.ncbi.nlm.nih.gov/entrez/query.fcgi?db=gene&cmd=Retrieve&dopt=full_report&list_uids=10644) | insulin-like growth factor 2 mRNA binding protein 2 |
| [Details](http://mirdb.org/cgi-bin/target_detail.cgi?targetID=1317007) | 268 | 60 | hsa-miR-29b-1-5p | [CD2AP](http://www.ncbi.nlm.nih.gov/entrez/query.fcgi?db=gene&cmd=Retrieve&dopt=full_report&list_uids=23607) | CD2-associated protein |
| [Details](http://mirdb.org/cgi-bin/target_detail.cgi?targetID=1317248) | 269 | 60 | hsa-miR-29b-1-5p | [MAPK9](http://www.ncbi.nlm.nih.gov/entrez/query.fcgi?db=gene&cmd=Retrieve&dopt=full_report&list_uids=5601) | mitogen-activated protein kinase 9 |
| [Details](http://mirdb.org/cgi-bin/target_detail.cgi?targetID=1317178) | 270 | 60 | hsa-miR-29b-1-5p | [COL4A6](http://www.ncbi.nlm.nih.gov/entrez/query.fcgi?db=gene&cmd=Retrieve&dopt=full_report&list_uids=1288) | collagen, type IV, alpha 6 |
| [Details](http://mirdb.org/cgi-bin/target_detail.cgi?targetID=1317242) | 271 | 60 | hsa-miR-29b-1-5p | [TULP4](http://www.ncbi.nlm.nih.gov/entrez/query.fcgi?db=gene&cmd=Retrieve&dopt=full_report&list_uids=56995) | tubby like protein 4 |
| [Details](http://mirdb.org/cgi-bin/target_detail.cgi?targetID=1317168) | 272 | 60 | hsa-miR-29b-1-5p | [RP11-35G9.3](http://www.ncbi.nlm.nih.gov/entrez/query.fcgi?db=gene&cmd=Retrieve&dopt=full_report&list_uids=100505549) | uncharacterized LOC100505549 |
| [Details](http://mirdb.org/cgi-bin/target_detail.cgi?targetID=1317345) | 273 | 60 | hsa-miR-29b-1-5p | [TEAD1](http://www.ncbi.nlm.nih.gov/entrez/query.fcgi?db=gene&cmd=Retrieve&dopt=full_report&list_uids=7003) | TEA domain family member 1 (SV40 transcriptional enhancer factor) |
| [Details](http://mirdb.org/cgi-bin/target_detail.cgi?targetID=1316974) | 274 | 60 | hsa-miR-29b-1-5p | [ZNF226](http://www.ncbi.nlm.nih.gov/entrez/query.fcgi?db=gene&cmd=Retrieve&dopt=full_report&list_uids=7769) | zinc finger protein 226 |
| [Details](http://mirdb.org/cgi-bin/target_detail.cgi?targetID=1317273) | 275 | 60 | hsa-miR-29b-1-5p | [FAM199X](http://www.ncbi.nlm.nih.gov/entrez/query.fcgi?db=gene&cmd=Retrieve&dopt=full_report&list_uids=139231) | family with sequence similarity 199, X-linked |
| [Details](http://mirdb.org/cgi-bin/target_detail.cgi?targetID=1317055) | 276 | 60 | hsa-miR-29b-1-5p | [GLTSCR1L](http://www.ncbi.nlm.nih.gov/entrez/query.fcgi?db=gene&cmd=Retrieve&dopt=full_report&list_uids=23506) | GLTSCR1-like |
| [Details](http://mirdb.org/cgi-bin/target_detail.cgi?targetID=1317059) | 277 | 60 | hsa-miR-29b-1-5p | [FAM169A](http://www.ncbi.nlm.nih.gov/entrez/query.fcgi?db=gene&cmd=Retrieve&dopt=full_report&list_uids=26049) | family with sequence similarity 169, member A |
| [Details](http://mirdb.org/cgi-bin/target_detail.cgi?targetID=1317210) | 278 | 60 | hsa-miR-29b-1-5p | [AIF1L](http://www.ncbi.nlm.nih.gov/entrez/query.fcgi?db=gene&cmd=Retrieve&dopt=full_report&list_uids=83543) | allograft inflammatory factor 1-like |
| [Details](http://mirdb.org/cgi-bin/target_detail.cgi?targetID=1317132) | 279 | 60 | hsa-miR-29b-1-5p | [COPS8](http://www.ncbi.nlm.nih.gov/entrez/query.fcgi?db=gene&cmd=Retrieve&dopt=full_report&list_uids=10920) | COP9 signalosome subunit 8 |
| [Details](http://mirdb.org/cgi-bin/target_detail.cgi?targetID=1317150) | 280 | 59 | hsa-miR-29b-1-5p | [DUSP16](http://www.ncbi.nlm.nih.gov/entrez/query.fcgi?db=gene&cmd=Retrieve&dopt=full_report&list_uids=80824) | dual specificity phosphatase 16 |
| [Details](http://mirdb.org/cgi-bin/target_detail.cgi?targetID=1317093) | 281 | 59 | hsa-miR-29b-1-5p | [ZBED1](http://www.ncbi.nlm.nih.gov/entrez/query.fcgi?db=gene&cmd=Retrieve&dopt=full_report&list_uids=9189) | zinc finger, BED-type containing 1 |
| [Details](http://mirdb.org/cgi-bin/target_detail.cgi?targetID=1317086) | 282 | 59 | hsa-miR-29b-1-5p | [KCNH7](http://www.ncbi.nlm.nih.gov/entrez/query.fcgi?db=gene&cmd=Retrieve&dopt=full_report&list_uids=90134) | potassium voltage-gated channel, subfamily H (eag-related), member 7 |
| [Details](http://mirdb.org/cgi-bin/target_detail.cgi?targetID=1317261) | 283 | 59 | hsa-miR-29b-1-5p | [NR1D1](http://www.ncbi.nlm.nih.gov/entrez/query.fcgi?db=gene&cmd=Retrieve&dopt=full_report&list_uids=9572) | nuclear receptor subfamily 1, group D, member 1 |
| [Details](http://mirdb.org/cgi-bin/target_detail.cgi?targetID=1317155) | 284 | 59 | hsa-miR-29b-1-5p | [TMEM151B](http://www.ncbi.nlm.nih.gov/entrez/query.fcgi?db=gene&cmd=Retrieve&dopt=full_report&list_uids=441151) | transmembrane protein 151B |
| [Details](http://mirdb.org/cgi-bin/target_detail.cgi?targetID=1317009) | 285 | 59 | hsa-miR-29b-1-5p | [ZNF800](http://www.ncbi.nlm.nih.gov/entrez/query.fcgi?db=gene&cmd=Retrieve&dopt=full_report&list_uids=168850) | zinc finger protein 800 |
| [Details](http://mirdb.org/cgi-bin/target_detail.cgi?targetID=1317245) | 286 | 58 | hsa-miR-29b-1-5p | [STOML2](http://www.ncbi.nlm.nih.gov/entrez/query.fcgi?db=gene&cmd=Retrieve&dopt=full_report&list_uids=30968) | stomatin (EPB72)-like 2 |
| [Details](http://mirdb.org/cgi-bin/target_detail.cgi?targetID=1317013) | 287 | 58 | hsa-miR-29b-1-5p | [GABBR1](http://www.ncbi.nlm.nih.gov/entrez/query.fcgi?db=gene&cmd=Retrieve&dopt=full_report&list_uids=2550) | gamma-aminobutyric acid (GABA) B receptor, 1 |
| [Details](http://mirdb.org/cgi-bin/target_detail.cgi?targetID=1316990) | 288 | 58 | hsa-miR-29b-1-5p | [INSIG1](http://www.ncbi.nlm.nih.gov/entrez/query.fcgi?db=gene&cmd=Retrieve&dopt=full_report&list_uids=3638) | insulin induced gene 1 |
| [Details](http://mirdb.org/cgi-bin/target_detail.cgi?targetID=1317205) | 289 | 58 | hsa-miR-29b-1-5p | [FTSJ1](http://www.ncbi.nlm.nih.gov/entrez/query.fcgi?db=gene&cmd=Retrieve&dopt=full_report&list_uids=24140) | FtsJ RNA methyltransferase homolog 1 (E. coli) |
| [Details](http://mirdb.org/cgi-bin/target_detail.cgi?targetID=1317163) | 290 | 58 | hsa-miR-29b-1-5p | [C16orf58](http://www.ncbi.nlm.nih.gov/entrez/query.fcgi?db=gene&cmd=Retrieve&dopt=full_report&list_uids=64755) | chromosome 16 open reading frame 58 |
| [Details](http://mirdb.org/cgi-bin/target_detail.cgi?targetID=1317281) | 291 | 58 | hsa-miR-29b-1-5p | [AHDC1](http://www.ncbi.nlm.nih.gov/entrez/query.fcgi?db=gene&cmd=Retrieve&dopt=full_report&list_uids=27245) | AT hook, DNA binding motif, containing 1 |
| [Details](http://mirdb.org/cgi-bin/target_detail.cgi?targetID=1317033) | 292 | 57 | hsa-miR-29b-1-5p | [LRRK1](http://www.ncbi.nlm.nih.gov/entrez/query.fcgi?db=gene&cmd=Retrieve&dopt=full_report&list_uids=79705) | leucine-rich repeat kinase 1 |
| [Details](http://mirdb.org/cgi-bin/target_detail.cgi?targetID=1316954) | 293 | 57 | hsa-miR-29b-1-5p | [HECA](http://www.ncbi.nlm.nih.gov/entrez/query.fcgi?db=gene&cmd=Retrieve&dopt=full_report&list_uids=51696) | headcase homolog (Drosophila) |
| [Details](http://mirdb.org/cgi-bin/target_detail.cgi?targetID=1317031) | 294 | 57 | hsa-miR-29b-1-5p | [ARHGEF28](http://www.ncbi.nlm.nih.gov/entrez/query.fcgi?db=gene&cmd=Retrieve&dopt=full_report&list_uids=64283) | Rho guanine nucleotide exchange factor (GEF) 28 |
| [Details](http://mirdb.org/cgi-bin/target_detail.cgi?targetID=1317250) | 295 | 57 | hsa-miR-29b-1-5p | [PLS1](http://www.ncbi.nlm.nih.gov/entrez/query.fcgi?db=gene&cmd=Retrieve&dopt=full_report&list_uids=5357) | plastin 1 |
| [Details](http://mirdb.org/cgi-bin/target_detail.cgi?targetID=1317034) | 296 | 57 | hsa-miR-29b-1-5p | [LRRC58](http://www.ncbi.nlm.nih.gov/entrez/query.fcgi?db=gene&cmd=Retrieve&dopt=full_report&list_uids=116064) | leucine rich repeat containing 58 |
| [Details](http://mirdb.org/cgi-bin/target_detail.cgi?targetID=1317189) | 297 | 57 | hsa-miR-29b-1-5p | [HECTD2](http://www.ncbi.nlm.nih.gov/entrez/query.fcgi?db=gene&cmd=Retrieve&dopt=full_report&list_uids=143279) | HECT domain containing E3 ubiquitin protein ligase 2 |
| [Details](http://mirdb.org/cgi-bin/target_detail.cgi?targetID=1317181) | 298 | 57 | hsa-miR-29b-1-5p | [PTGS2](http://www.ncbi.nlm.nih.gov/entrez/query.fcgi?db=gene&cmd=Retrieve&dopt=full_report&list_uids=5743) | prostaglandin-endoperoxide synthase 2 (prostaglandin G/H synthase and cyclooxygenase) |
| [Details](http://mirdb.org/cgi-bin/target_detail.cgi?targetID=1317230) | 299 | 57 | hsa-miR-29b-1-5p | [SMG1](http://www.ncbi.nlm.nih.gov/entrez/query.fcgi?db=gene&cmd=Retrieve&dopt=full_report&list_uids=23049) | SMG1 phosphatidylinositol 3-kinase-related kinase |
| [Details](http://mirdb.org/cgi-bin/target_detail.cgi?targetID=1317247) | 300 | 57 | hsa-miR-29b-1-5p | [PLA2G12A](http://www.ncbi.nlm.nih.gov/entrez/query.fcgi?db=gene&cmd=Retrieve&dopt=full_report&list_uids=81579) | phospholipase A2, group XIIA |
| [Details](http://mirdb.org/cgi-bin/target_detail.cgi?targetID=1317319) | 301 | 56 | hsa-miR-29b-1-5p | [SLC35A5](http://www.ncbi.nlm.nih.gov/entrez/query.fcgi?db=gene&cmd=Retrieve&dopt=full_report&list_uids=55032) | solute carrier family 35, member A5 |
| [Details](http://mirdb.org/cgi-bin/target_detail.cgi?targetID=1317291) | 302 | 56 | hsa-miR-29b-1-5p | [ETF1](http://www.ncbi.nlm.nih.gov/entrez/query.fcgi?db=gene&cmd=Retrieve&dopt=full_report&list_uids=2107) | eukaryotic translation termination factor 1 |
| [Details](http://mirdb.org/cgi-bin/target_detail.cgi?targetID=1316975) | 303 | 56 | hsa-miR-29b-1-5p | [HMGCLL1](http://www.ncbi.nlm.nih.gov/entrez/query.fcgi?db=gene&cmd=Retrieve&dopt=full_report&list_uids=54511) | 3-hydroxymethyl-3-methylglutaryl-CoA lyase-like 1 |
| [Details](http://mirdb.org/cgi-bin/target_detail.cgi?targetID=1317119) | 304 | 56 | hsa-miR-29b-1-5p | [TMEM254](http://www.ncbi.nlm.nih.gov/entrez/query.fcgi?db=gene&cmd=Retrieve&dopt=full_report&list_uids=80195) | transmembrane protein 254 |
| [Details](http://mirdb.org/cgi-bin/target_detail.cgi?targetID=1317234) | 305 | 56 | hsa-miR-29b-1-5p | [TBL1XR1](http://www.ncbi.nlm.nih.gov/entrez/query.fcgi?db=gene&cmd=Retrieve&dopt=full_report&list_uids=79718) | transducin (beta)-like 1 X-linked receptor 1 |
| [Details](http://mirdb.org/cgi-bin/target_detail.cgi?targetID=1317282) | 306 | 56 | hsa-miR-29b-1-5p | [STIM2](http://www.ncbi.nlm.nih.gov/entrez/query.fcgi?db=gene&cmd=Retrieve&dopt=full_report&list_uids=57620) | stromal interaction molecule 2 |
| [Details](http://mirdb.org/cgi-bin/target_detail.cgi?targetID=1317137) | 307 | 56 | hsa-miR-29b-1-5p | [CPPED1](http://www.ncbi.nlm.nih.gov/entrez/query.fcgi?db=gene&cmd=Retrieve&dopt=full_report&list_uids=55313) | calcineurin-like phosphoesterase domain containing 1 |
| [Details](http://mirdb.org/cgi-bin/target_detail.cgi?targetID=1317000) | 308 | 56 | hsa-miR-29b-1-5p | [UBE2J1](http://www.ncbi.nlm.nih.gov/entrez/query.fcgi?db=gene&cmd=Retrieve&dopt=full_report&list_uids=51465) | ubiquitin-conjugating enzyme E2, J1 |
| [Details](http://mirdb.org/cgi-bin/target_detail.cgi?targetID=1316966) | 309 | 56 | hsa-miR-29b-1-5p | [RBM42](http://www.ncbi.nlm.nih.gov/entrez/query.fcgi?db=gene&cmd=Retrieve&dopt=full_report&list_uids=79171) | RNA binding motif protein 42 |
| [Details](http://mirdb.org/cgi-bin/target_detail.cgi?targetID=1317310) | 310 | 56 | hsa-miR-29b-1-5p | [GPRIN3](http://www.ncbi.nlm.nih.gov/entrez/query.fcgi?db=gene&cmd=Retrieve&dopt=full_report&list_uids=285513) | GPRIN family member 3 |
| [Details](http://mirdb.org/cgi-bin/target_detail.cgi?targetID=1317078) | 311 | 56 | hsa-miR-29b-1-5p | [MRFAP1](http://www.ncbi.nlm.nih.gov/entrez/query.fcgi?db=gene&cmd=Retrieve&dopt=full_report&list_uids=93621) | Morf4 family associated protein 1 |
| [Details](http://mirdb.org/cgi-bin/target_detail.cgi?targetID=1317309) | 312 | 56 | hsa-miR-29b-1-5p | [RNF14](http://www.ncbi.nlm.nih.gov/entrez/query.fcgi?db=gene&cmd=Retrieve&dopt=full_report&list_uids=9604) | ring finger protein 14 |
| [Details](http://mirdb.org/cgi-bin/target_detail.cgi?targetID=1317342) | 313 | 56 | hsa-miR-29b-1-5p | [MLXIP](http://www.ncbi.nlm.nih.gov/entrez/query.fcgi?db=gene&cmd=Retrieve&dopt=full_report&list_uids=22877) | MLX interacting protein |
| [Details](http://mirdb.org/cgi-bin/target_detail.cgi?targetID=1317293) | 314 | 55 | hsa-miR-29b-1-5p | [FSD1L](http://www.ncbi.nlm.nih.gov/entrez/query.fcgi?db=gene&cmd=Retrieve&dopt=full_report&list_uids=83856) | fibronectin type III and SPRY domain containing 1-like |
| [Details](http://mirdb.org/cgi-bin/target_detail.cgi?targetID=1317280) | 315 | 55 | hsa-miR-29b-1-5p | [CNTNAP2](http://www.ncbi.nlm.nih.gov/entrez/query.fcgi?db=gene&cmd=Retrieve&dopt=full_report&list_uids=26047) | contactin associated protein-like 2 |
| [Details](http://mirdb.org/cgi-bin/target_detail.cgi?targetID=1317209) | 316 | 55 | hsa-miR-29b-1-5p | [CLDN18](http://www.ncbi.nlm.nih.gov/entrez/query.fcgi?db=gene&cmd=Retrieve&dopt=full_report&list_uids=51208) | claudin 18 |
| [Details](http://mirdb.org/cgi-bin/target_detail.cgi?targetID=1316977) | 317 | 55 | hsa-miR-29b-1-5p | [MAT2A](http://www.ncbi.nlm.nih.gov/entrez/query.fcgi?db=gene&cmd=Retrieve&dopt=full_report&list_uids=4144) | methionine adenosyltransferase II, alpha |
| [Details](http://mirdb.org/cgi-bin/target_detail.cgi?targetID=1316981) | 318 | 55 | hsa-miR-29b-1-5p | [MGAT4A](http://www.ncbi.nlm.nih.gov/entrez/query.fcgi?db=gene&cmd=Retrieve&dopt=full_report&list_uids=11320) | mannosyl (alpha-1,3-)-glycoprotein beta-1,4-N-acetylglucosaminyltransferase, isozyme A |
| [Details](http://mirdb.org/cgi-bin/target_detail.cgi?targetID=1317052) | 319 | 55 | hsa-miR-29b-1-5p | [TMED7](http://www.ncbi.nlm.nih.gov/entrez/query.fcgi?db=gene&cmd=Retrieve&dopt=full_report&list_uids=51014) | transmembrane emp24 protein transport domain containing 7 |
| [Details](http://mirdb.org/cgi-bin/target_detail.cgi?targetID=1317243) | 320 | 55 | hsa-miR-29b-1-5p | [MCL1](http://www.ncbi.nlm.nih.gov/entrez/query.fcgi?db=gene&cmd=Retrieve&dopt=full_report&list_uids=4170) | myeloid cell leukemia 1 |
| [Details](http://mirdb.org/cgi-bin/target_detail.cgi?targetID=1317099) | 321 | 55 | hsa-miR-29b-1-5p | [DUS4L](http://www.ncbi.nlm.nih.gov/entrez/query.fcgi?db=gene&cmd=Retrieve&dopt=full_report&list_uids=11062) | dihydrouridine synthase 4-like (S. cerevisiae) |
| [Details](http://mirdb.org/cgi-bin/target_detail.cgi?targetID=1317258) | 322 | 55 | hsa-miR-29b-1-5p | [SLC10A1](http://www.ncbi.nlm.nih.gov/entrez/query.fcgi?db=gene&cmd=Retrieve&dopt=full_report&list_uids=6554) | solute carrier family 10 (sodium/bile acid cotransporter), member 1 |
| [Details](http://mirdb.org/cgi-bin/target_detail.cgi?targetID=1317107) | 323 | 55 | hsa-miR-29b-1-5p | [DIEXF](http://www.ncbi.nlm.nih.gov/entrez/query.fcgi?db=gene&cmd=Retrieve&dopt=full_report&list_uids=27042) | digestive organ expansion factor homolog (zebrafish) |
| [Details](http://mirdb.org/cgi-bin/target_detail.cgi?targetID=1317305) | 324 | 55 | hsa-miR-29b-1-5p | [UBE2R2](http://www.ncbi.nlm.nih.gov/entrez/query.fcgi?db=gene&cmd=Retrieve&dopt=full_report&list_uids=54926) | ubiquitin-conjugating enzyme E2R 2 |
| [Details](http://mirdb.org/cgi-bin/target_detail.cgi?targetID=1317014) | 325 | 55 | hsa-miR-29b-1-5p | [TEX12](http://www.ncbi.nlm.nih.gov/entrez/query.fcgi?db=gene&cmd=Retrieve&dopt=full_report&list_uids=56158) | testis expressed 12 |
| [Details](http://mirdb.org/cgi-bin/target_detail.cgi?targetID=1317126) | 326 | 55 | hsa-miR-29b-1-5p | [GRIN2A](http://www.ncbi.nlm.nih.gov/entrez/query.fcgi?db=gene&cmd=Retrieve&dopt=full_report&list_uids=2903) | glutamate receptor, ionotropic, N-methyl D-aspartate 2A |
| [Details](http://mirdb.org/cgi-bin/target_detail.cgi?targetID=1317179) | 327 | 55 | hsa-miR-29b-1-5p | [TKTL1](http://www.ncbi.nlm.nih.gov/entrez/query.fcgi?db=gene&cmd=Retrieve&dopt=full_report&list_uids=8277) | transketolase-like 1 |
| [Details](http://mirdb.org/cgi-bin/target_detail.cgi?targetID=1317134) | 328 | 55 | hsa-miR-29b-1-5p | [PRMT8](http://www.ncbi.nlm.nih.gov/entrez/query.fcgi?db=gene&cmd=Retrieve&dopt=full_report&list_uids=56341) | protein arginine methyltransferase 8 |
| [Details](http://mirdb.org/cgi-bin/target_detail.cgi?targetID=1316982) | 329 | 55 | hsa-miR-29b-1-5p | [CASD1](http://www.ncbi.nlm.nih.gov/entrez/query.fcgi?db=gene&cmd=Retrieve&dopt=full_report&list_uids=64921) | CAS1 domain containing 1 |
| [Details](http://mirdb.org/cgi-bin/target_detail.cgi?targetID=1316972) | 330 | 54 | hsa-miR-29b-1-5p | [N4BP1](http://www.ncbi.nlm.nih.gov/entrez/query.fcgi?db=gene&cmd=Retrieve&dopt=full_report&list_uids=9683) | NEDD4 binding protein 1 |
| [Details](http://mirdb.org/cgi-bin/target_detail.cgi?targetID=1317335) | 331 | 54 | hsa-miR-29b-1-5p | [ARHGEF18](http://www.ncbi.nlm.nih.gov/entrez/query.fcgi?db=gene&cmd=Retrieve&dopt=full_report&list_uids=23370) | Rho/Rac guanine nucleotide exchange factor (GEF) 18 |
| [Details](http://mirdb.org/cgi-bin/target_detail.cgi?targetID=1317323) | 332 | 54 | hsa-miR-29b-1-5p | [SMC4](http://www.ncbi.nlm.nih.gov/entrez/query.fcgi?db=gene&cmd=Retrieve&dopt=full_report&list_uids=10051) | structural maintenance of chromosomes 4 |
| [Details](http://mirdb.org/cgi-bin/target_detail.cgi?targetID=1317020) | 333 | 54 | hsa-miR-29b-1-5p | [RAP1B](http://www.ncbi.nlm.nih.gov/entrez/query.fcgi?db=gene&cmd=Retrieve&dopt=full_report&list_uids=5908) | RAP1B, member of RAS oncogene family |
| [Details](http://mirdb.org/cgi-bin/target_detail.cgi?targetID=1317288) | 334 | 54 | hsa-miR-29b-1-5p | [CHDH](http://www.ncbi.nlm.nih.gov/entrez/query.fcgi?db=gene&cmd=Retrieve&dopt=full_report&list_uids=55349) | choline dehydrogenase |
| [Details](http://mirdb.org/cgi-bin/target_detail.cgi?targetID=1317306) | 335 | 54 | hsa-miR-29b-1-5p | [TERF2](http://www.ncbi.nlm.nih.gov/entrez/query.fcgi?db=gene&cmd=Retrieve&dopt=full_report&list_uids=7014) | telomeric repeat binding factor 2 |
| [Details](http://mirdb.org/cgi-bin/target_detail.cgi?targetID=1317094) | 336 | 54 | hsa-miR-29b-1-5p | [EIF2AK1](http://www.ncbi.nlm.nih.gov/entrez/query.fcgi?db=gene&cmd=Retrieve&dopt=full_report&list_uids=27102) | eukaryotic translation initiation factor 2-alpha kinase 1 |
| [Details](http://mirdb.org/cgi-bin/target_detail.cgi?targetID=1317145) | 337 | 54 | hsa-miR-29b-1-5p | [ZFP90](http://www.ncbi.nlm.nih.gov/entrez/query.fcgi?db=gene&cmd=Retrieve&dopt=full_report&list_uids=146198) | ZFP90 zinc finger protein |
| [Details](http://mirdb.org/cgi-bin/target_detail.cgi?targetID=1317235) | 338 | 54 | hsa-miR-29b-1-5p | [ALDH1B1](http://www.ncbi.nlm.nih.gov/entrez/query.fcgi?db=gene&cmd=Retrieve&dopt=full_report&list_uids=219) | aldehyde dehydrogenase 1 family, member B1 |
| [Details](http://mirdb.org/cgi-bin/target_detail.cgi?targetID=1317174) | 339 | 54 | hsa-miR-29b-1-5p | [HTRA4](http://www.ncbi.nlm.nih.gov/entrez/query.fcgi?db=gene&cmd=Retrieve&dopt=full_report&list_uids=203100) | HtrA serine peptidase 4 |
| [Details](http://mirdb.org/cgi-bin/target_detail.cgi?targetID=1317180) | 340 | 54 | hsa-miR-29b-1-5p | [ZNF608](http://www.ncbi.nlm.nih.gov/entrez/query.fcgi?db=gene&cmd=Retrieve&dopt=full_report&list_uids=57507) | zinc finger protein 608 |
| [Details](http://mirdb.org/cgi-bin/target_detail.cgi?targetID=1317045) | 341 | 54 | hsa-miR-29b-1-5p | [TCF12](http://www.ncbi.nlm.nih.gov/entrez/query.fcgi?db=gene&cmd=Retrieve&dopt=full_report&list_uids=6938) | transcription factor 12 |
| [Details](http://mirdb.org/cgi-bin/target_detail.cgi?targetID=1317127) | 342 | 54 | hsa-miR-29b-1-5p | [CASK](http://www.ncbi.nlm.nih.gov/entrez/query.fcgi?db=gene&cmd=Retrieve&dopt=full_report&list_uids=8573) | calcium/calmodulin-dependent serine protein kinase (MAGUK family) |
| [Details](http://mirdb.org/cgi-bin/target_detail.cgi?targetID=1317263) | 343 | 53 | hsa-miR-29b-1-5p | [CBLN2](http://www.ncbi.nlm.nih.gov/entrez/query.fcgi?db=gene&cmd=Retrieve&dopt=full_report&list_uids=147381) | cerebellin 2 precursor |
| [Details](http://mirdb.org/cgi-bin/target_detail.cgi?targetID=1317070) | 344 | 53 | hsa-miR-29b-1-5p | [CDRT1](http://www.ncbi.nlm.nih.gov/entrez/query.fcgi?db=gene&cmd=Retrieve&dopt=full_report&list_uids=374286) | CMT1A duplicated region transcript 1 |
| [Details](http://mirdb.org/cgi-bin/target_detail.cgi?targetID=1317073) | 345 | 53 | hsa-miR-29b-1-5p | [QRICH1](http://www.ncbi.nlm.nih.gov/entrez/query.fcgi?db=gene&cmd=Retrieve&dopt=full_report&list_uids=54870) | glutamine-rich 1 |
| [Details](http://mirdb.org/cgi-bin/target_detail.cgi?targetID=1317203) | 346 | 53 | hsa-miR-29b-1-5p | [CCDC177](http://www.ncbi.nlm.nih.gov/entrez/query.fcgi?db=gene&cmd=Retrieve&dopt=full_report&list_uids=56936) | coiled-coil domain containing 177 |
| [Details](http://mirdb.org/cgi-bin/target_detail.cgi?targetID=1317329) | 347 | 53 | hsa-miR-29b-1-5p | [HNRNPAB](http://www.ncbi.nlm.nih.gov/entrez/query.fcgi?db=gene&cmd=Retrieve&dopt=full_report&list_uids=3182) | heterogeneous nuclear ribonucleoprotein A/B |
| [Details](http://mirdb.org/cgi-bin/target_detail.cgi?targetID=1317204) | 348 | 53 | hsa-miR-29b-1-5p | [SFMBT2](http://www.ncbi.nlm.nih.gov/entrez/query.fcgi?db=gene&cmd=Retrieve&dopt=full_report&list_uids=57713) | Scm-like with four mbt domains 2 |
| [Details](http://mirdb.org/cgi-bin/target_detail.cgi?targetID=1316964) | 349 | 53 | hsa-miR-29b-1-5p | [HEPHL1](http://www.ncbi.nlm.nih.gov/entrez/query.fcgi?db=gene&cmd=Retrieve&dopt=full_report&list_uids=341208) | hephaestin-like 1 |
| [Details](http://mirdb.org/cgi-bin/target_detail.cgi?targetID=1317125) | 350 | 53 | hsa-miR-29b-1-5p | [CCNY](http://www.ncbi.nlm.nih.gov/entrez/query.fcgi?db=gene&cmd=Retrieve&dopt=full_report&list_uids=219771) | cyclin Y |
| [Details](http://mirdb.org/cgi-bin/target_detail.cgi?targetID=1317287) | 351 | 53 | hsa-miR-29b-1-5p | [RGS4](http://www.ncbi.nlm.nih.gov/entrez/query.fcgi?db=gene&cmd=Retrieve&dopt=full_report&list_uids=5999) | regulator of G-protein signaling 4 |
| [Details](http://mirdb.org/cgi-bin/target_detail.cgi?targetID=1317065) | 352 | 53 | hsa-miR-29b-1-5p | [ANKRD27](http://www.ncbi.nlm.nih.gov/entrez/query.fcgi?db=gene&cmd=Retrieve&dopt=full_report&list_uids=84079) | ankyrin repeat domain 27 (VPS9 domain) |
| [Details](http://mirdb.org/cgi-bin/target_detail.cgi?targetID=1317271) | 353 | 53 | hsa-miR-29b-1-5p | [PTPN4](http://www.ncbi.nlm.nih.gov/entrez/query.fcgi?db=gene&cmd=Retrieve&dopt=full_report&list_uids=5775) | protein tyrosine phosphatase, non-receptor type 4 (megakaryocyte) |
| [Details](http://mirdb.org/cgi-bin/target_detail.cgi?targetID=1316962) | 354 | 53 | hsa-miR-29b-1-5p | [NMT1](http://www.ncbi.nlm.nih.gov/entrez/query.fcgi?db=gene&cmd=Retrieve&dopt=full_report&list_uids=4836) | N-myristoyltransferase 1 |
| [Details](http://mirdb.org/cgi-bin/target_detail.cgi?targetID=1317056) | 355 | 53 | hsa-miR-29b-1-5p | [MTRF1L](http://www.ncbi.nlm.nih.gov/entrez/query.fcgi?db=gene&cmd=Retrieve&dopt=full_report&list_uids=54516) | mitochondrial translational release factor 1-like |
| [Details](http://mirdb.org/cgi-bin/target_detail.cgi?targetID=1317018) | 356 | 53 | hsa-miR-29b-1-5p | [CNR2](http://www.ncbi.nlm.nih.gov/entrez/query.fcgi?db=gene&cmd=Retrieve&dopt=full_report&list_uids=1269) | cannabinoid receptor 2 (macrophage) |
| [Details](http://mirdb.org/cgi-bin/target_detail.cgi?targetID=1317188) | 357 | 52 | hsa-miR-29b-1-5p | [CPSF2](http://www.ncbi.nlm.nih.gov/entrez/query.fcgi?db=gene&cmd=Retrieve&dopt=full_report&list_uids=53981) | cleavage and polyadenylation specific factor 2, 100kDa |
| [Details](http://mirdb.org/cgi-bin/target_detail.cgi?targetID=1317321) | 358 | 52 | hsa-miR-29b-1-5p | [REEP5](http://www.ncbi.nlm.nih.gov/entrez/query.fcgi?db=gene&cmd=Retrieve&dopt=full_report&list_uids=7905) | receptor accessory protein 5 |
| [Details](http://mirdb.org/cgi-bin/target_detail.cgi?targetID=1316955) | 359 | 52 | hsa-miR-29b-1-5p | [ADAL](http://www.ncbi.nlm.nih.gov/entrez/query.fcgi?db=gene&cmd=Retrieve&dopt=full_report&list_uids=161823) | adenosine deaminase-like |
| [Details](http://mirdb.org/cgi-bin/target_detail.cgi?targetID=1317143) | 360 | 52 | hsa-miR-29b-1-5p | [HP1BP3](http://www.ncbi.nlm.nih.gov/entrez/query.fcgi?db=gene&cmd=Retrieve&dopt=full_report&list_uids=50809) | heterochromatin protein 1, binding protein 3 |
| [Details](http://mirdb.org/cgi-bin/target_detail.cgi?targetID=1317017) | 361 | 52 | hsa-miR-29b-1-5p | [MAST4](http://www.ncbi.nlm.nih.gov/entrez/query.fcgi?db=gene&cmd=Retrieve&dopt=full_report&list_uids=375449) | microtubule associated serine/threonine kinase family member 4 |
| [Details](http://mirdb.org/cgi-bin/target_detail.cgi?targetID=1317268) | 362 | 52 | hsa-miR-29b-1-5p | [FBXO17](http://www.ncbi.nlm.nih.gov/entrez/query.fcgi?db=gene&cmd=Retrieve&dopt=full_report&list_uids=115290) | F-box protein 17 |
| [Details](http://mirdb.org/cgi-bin/target_detail.cgi?targetID=1317157) | 363 | 52 | hsa-miR-29b-1-5p | [SNX9](http://www.ncbi.nlm.nih.gov/entrez/query.fcgi?db=gene&cmd=Retrieve&dopt=full_report&list_uids=51429) | sorting nexin 9 |
| [Details](http://mirdb.org/cgi-bin/target_detail.cgi?targetID=1317156) | 364 | 52 | hsa-miR-29b-1-5p | [RET](http://www.ncbi.nlm.nih.gov/entrez/query.fcgi?db=gene&cmd=Retrieve&dopt=full_report&list_uids=5979) | ret proto-oncogene |
| [Details](http://mirdb.org/cgi-bin/target_detail.cgi?targetID=1317255) | 365 | 52 | hsa-miR-29b-1-5p | [ASTN1](http://www.ncbi.nlm.nih.gov/entrez/query.fcgi?db=gene&cmd=Retrieve&dopt=full_report&list_uids=460) | astrotactin 1 |
| [Details](http://mirdb.org/cgi-bin/target_detail.cgi?targetID=1317152) | 366 | 52 | hsa-miR-29b-1-5p | [KLHL24](http://www.ncbi.nlm.nih.gov/entrez/query.fcgi?db=gene&cmd=Retrieve&dopt=full_report&list_uids=54800) | kelch-like family member 24 |
| [Details](http://mirdb.org/cgi-bin/target_detail.cgi?targetID=1317166) | 367 | 52 | hsa-miR-29b-1-5p | [TRIM60](http://www.ncbi.nlm.nih.gov/entrez/query.fcgi?db=gene&cmd=Retrieve&dopt=full_report&list_uids=166655) | tripartite motif containing 60 |
| [Details](http://mirdb.org/cgi-bin/target_detail.cgi?targetID=1317091) | 368 | 52 | hsa-miR-29b-1-5p | [LCE1B](http://www.ncbi.nlm.nih.gov/entrez/query.fcgi?db=gene&cmd=Retrieve&dopt=full_report&list_uids=353132) | late cornified envelope 1B |
| [Details](http://mirdb.org/cgi-bin/target_detail.cgi?targetID=1317002) | 369 | 52 | hsa-miR-29b-1-5p | [BLOC1S6](http://www.ncbi.nlm.nih.gov/entrez/query.fcgi?db=gene&cmd=Retrieve&dopt=full_report&list_uids=26258) | biogenesis of lysosomal organelles complex-1, subunit 6, pallidin |
| [Details](http://mirdb.org/cgi-bin/target_detail.cgi?targetID=1317097) | 370 | 52 | hsa-miR-29b-1-5p | [WDR55](http://www.ncbi.nlm.nih.gov/entrez/query.fcgi?db=gene&cmd=Retrieve&dopt=full_report&list_uids=54853) | WD repeat domain 55 |
| [Details](http://mirdb.org/cgi-bin/target_detail.cgi?targetID=1317147) | 371 | 51 | hsa-miR-29b-1-5p | [MBD1](http://www.ncbi.nlm.nih.gov/entrez/query.fcgi?db=gene&cmd=Retrieve&dopt=full_report&list_uids=4152) | methyl-CpG binding domain protein 1 |
| [Details](http://mirdb.org/cgi-bin/target_detail.cgi?targetID=1317158) | 372 | 51 | hsa-miR-29b-1-5p | [RAP2A](http://www.ncbi.nlm.nih.gov/entrez/query.fcgi?db=gene&cmd=Retrieve&dopt=full_report&list_uids=5911) | RAP2A, member of RAS oncogene family |
| [Details](http://mirdb.org/cgi-bin/target_detail.cgi?targetID=1317267) | 373 | 51 | hsa-miR-29b-1-5p | [SGCB](http://www.ncbi.nlm.nih.gov/entrez/query.fcgi?db=gene&cmd=Retrieve&dopt=full_report&list_uids=6443) | sarcoglycan, beta (43kDa dystrophin-associated glycoprotein) |
| [Details](http://mirdb.org/cgi-bin/target_detail.cgi?targetID=1317043) | 374 | 51 | hsa-miR-29b-1-5p | [GTF2E1](http://www.ncbi.nlm.nih.gov/entrez/query.fcgi?db=gene&cmd=Retrieve&dopt=full_report&list_uids=2960) | general transcription factor IIE, polypeptide 1, alpha 56kDa |
| [Details](http://mirdb.org/cgi-bin/target_detail.cgi?targetID=1317039) | 375 | 51 | hsa-miR-29b-1-5p | [SPON1](http://www.ncbi.nlm.nih.gov/entrez/query.fcgi?db=gene&cmd=Retrieve&dopt=full_report&list_uids=10418) | spondin 1, extracellular matrix protein |
| [Details](http://mirdb.org/cgi-bin/target_detail.cgi?targetID=1316985) | 376 | 51 | hsa-miR-29b-1-5p | [PRNP](http://www.ncbi.nlm.nih.gov/entrez/query.fcgi?db=gene&cmd=Retrieve&dopt=full_report&list_uids=5621) | prion protein |
| [Details](http://mirdb.org/cgi-bin/target_detail.cgi?targetID=1317149) | 377 | 51 | hsa-miR-29b-1-5p | [COPS5](http://www.ncbi.nlm.nih.gov/entrez/query.fcgi?db=gene&cmd=Retrieve&dopt=full_report&list_uids=10987) | COP9 signalosome subunit 5 |
| [Details](http://mirdb.org/cgi-bin/target_detail.cgi?targetID=1317062) | 378 | 51 | hsa-miR-29b-1-5p | [LAMB2](http://www.ncbi.nlm.nih.gov/entrez/query.fcgi?db=gene&cmd=Retrieve&dopt=full_report&list_uids=3913) | laminin, beta 2 (laminin S) |
| [Details](http://mirdb.org/cgi-bin/target_detail.cgi?targetID=1317098) | 379 | 51 | hsa-miR-29b-1-5p | [VPS35](http://www.ncbi.nlm.nih.gov/entrez/query.fcgi?db=gene&cmd=Retrieve&dopt=full_report&list_uids=55737) | vacuolar protein sorting 35 homolog (S. cerevisiae) |
| [Details](http://mirdb.org/cgi-bin/target_detail.cgi?targetID=1317061) | 380 | 51 | hsa-miR-29b-1-5p | [CAV3](http://www.ncbi.nlm.nih.gov/entrez/query.fcgi?db=gene&cmd=Retrieve&dopt=full_report&list_uids=859) | caveolin 3 |
| [Details](http://mirdb.org/cgi-bin/target_detail.cgi?targetID=1317111) | 381 | 50 | hsa-miR-29b-1-5p | [RANBP17](http://www.ncbi.nlm.nih.gov/entrez/query.fcgi?db=gene&cmd=Retrieve&dopt=full_report&list_uids=64901) | RAN binding protein 17 |
| [Details](http://mirdb.org/cgi-bin/target_detail.cgi?targetID=1317069) | 382 | 50 | hsa-miR-29b-1-5p | [NEUROD6](http://www.ncbi.nlm.nih.gov/entrez/query.fcgi?db=gene&cmd=Retrieve&dopt=full_report&list_uids=63974) | neuronal differentiation 6 |
| [Details](http://mirdb.org/cgi-bin/target_detail.cgi?targetID=1316957) | 383 | 50 | hsa-miR-29b-1-5p | [GRM5](http://www.ncbi.nlm.nih.gov/entrez/query.fcgi?db=gene&cmd=Retrieve&dopt=full_report&list_uids=2915) | glutamate receptor, metabotropic 5 |
| [Details](http://mirdb.org/cgi-bin/target_detail.cgi?targetID=1317221) | 384 | 50 | hsa-miR-29b-1-5p | [ZNF384](http://www.ncbi.nlm.nih.gov/entrez/query.fcgi?db=gene&cmd=Retrieve&dopt=full_report&list_uids=171017) | zinc finger protein 384 |
| [Details](http://mirdb.org/cgi-bin/target_detail.cgi?targetID=1317067) | 385 | 50 | hsa-miR-29b-1-5p | [ZNF649](http://www.ncbi.nlm.nih.gov/entrez/query.fcgi?db=gene&cmd=Retrieve&dopt=full_report&list_uids=65251) | zinc finger protein 649 |
| [Details](http://mirdb.org/cgi-bin/target_detail.cgi?targetID=1317295) | 386 | 50 | hsa-miR-29b-1-5p | [DNAJC10](http://www.ncbi.nlm.nih.gov/entrez/query.fcgi?db=gene&cmd=Retrieve&dopt=full_report&list_uids=54431) | DnaJ (Hsp40) homolog, subfamily C, member 10 |
| [Details](http://mirdb.org/cgi-bin/target_detail.cgi?targetID=1317075) | 387 | 50 | hsa-miR-29b-1-5p | [EIF2S3](http://www.ncbi.nlm.nih.gov/entrez/query.fcgi?db=gene&cmd=Retrieve&dopt=full_report&list_uids=1968) | eukaryotic translation initiation factor 2, subunit 3 gamma, 52kDa |
| [Details](http://mirdb.org/cgi-bin/target_detail.cgi?targetID=1317089) | 388 | 50 | hsa-miR-29b-1-5p | [PIK3C2B](http://www.ncbi.nlm.nih.gov/entrez/query.fcgi?db=gene&cmd=Retrieve&dopt=full_report&list_uids=5287) | phosphatidylinositol-4-phosphate 3-kinase, catalytic subunit type 2 beta |
| [Details](http://mirdb.org/cgi-bin/target_detail.cgi?targetID=1316994) | 389 | 50 | hsa-miR-29b-1-5p | [CALML4](http://www.ncbi.nlm.nih.gov/entrez/query.fcgi?db=gene&cmd=Retrieve&dopt=full_report&list_uids=91860) | calmodulin-like 4 |
| [Details](http://mirdb.org/cgi-bin/target_detail.cgi?targetID=1317118) | 390 | 50 | hsa-miR-29b-1-5p | [PCDH19](http://www.ncbi.nlm.nih.gov/entrez/query.fcgi?db=gene&cmd=Retrieve&dopt=full_report&list_uids=57526) | protocadherin 19 |
| [Details](http://mirdb.org/cgi-bin/target_detail.cgi?targetID=1317081) | 391 | 50 | hsa-miR-29b-1-5p | [BIRC2](http://www.ncbi.nlm.nih.gov/entrez/query.fcgi?db=gene&cmd=Retrieve&dopt=full_report&list_uids=329) | baculoviral IAP repeat containing 2 |
| [Details](http://mirdb.org/cgi-bin/target_detail.cgi?targetID=1317142) | 392 | 50 | hsa-miR-29b-1-5p | [EBF4](http://www.ncbi.nlm.nih.gov/entrez/query.fcgi?db=gene&cmd=Retrieve&dopt=full_report&list_uids=57593) | early B-cell factor 4 |
| [Details](http://mirdb.org/cgi-bin/target_detail.cgi?targetID=1316979) | 393 | 50 | hsa-miR-29b-1-5p | [HOXA3](http://www.ncbi.nlm.nih.gov/entrez/query.fcgi?db=gene&cmd=Retrieve&dopt=full_report&list_uids=3200) | homeobox A3 |
