# Supplementary material for: Suppressive role exerted by microRNA-29b-1-5p in triple negative breast cancer through SPIN1 regulation
Source: Oncotarget. 2017 Mar 7;8(17):28939–58. doi: 10.18632/oncotarget.15960 (PMC5438704; doi:10.18632/oncotarget.15960)
Supplement: Supplementary file 3 [file oncotarget-08-28939-s003.doc]

| Supplementary Table 2: Clinicopathologic characteristics of patients | | | | |
| --- | --- | --- | --- | --- |
| **Patient** | **Age** | **On Tx** | **Specimen type** | **Type** |
| N1 | 47 | NO | Normal |  |
| N2 | 16 | NO | Fibroadenoma - check for normal tissue |  |
| N3 | 46 | NO | Unremarkable sections from TNBC pt (T8) |  |
| N4 | 35 | NO | Normal breast tissue blocks from tumour case (T2) |  |
| N5 | 30 | NO | Normal breast tissue blocks from tumour case (T1) |  |
| N6 | 30 | NO | Unremarkable sections from TNBC pt (T5) |  |
| T1 | 30 | NO | TNBC | Lymphoepithelial type breast carcinoma, poorly differentiated GIII |
| T2 | 35 | NO | TNBC | GIII ductal, basal phenotype |
| T3 | 38 | NO | TNBC | Grade III infiltrative Ductal Carcinoma, Triple Negative |
| T4 | 32 | NO | TNBC | Grade III infiltrative Ductal Carcinoma, Triple Negative |
| T5 | 30 | NO | TNBC | Grade III infiltrative Ductal Carcinoma, Triple Negative |
| T6 | 45 | NO | TNBC | Grade III infiltrative Ductal Carcinoma, Triple Negative Basal phenotype |
| T7 | 46 | NO | TNBC | Grade III infiltrative Ductal Carcinoma, Triple Negative |
| T8 | 46 | NO | TNBC | Grade III infiltrative Ductal Carcinoma, Triple Negative, Heterogenous but both types TNBC |
| T9 | 41 | NO | TNBC | Grade III infiltrative Ductal Carcinoma, Triple Negative Basal phenotype |
| T10 | 44 | NO | TNBC | Grade III infiltrative Ductal Carcinoma, Triple Negative |
| T11 | 55 | NO | TNBC | Grade III infiltrative Ductal Carcinoma, Triple Negative Basal phenotype |
| T12 | 56 | NO | TNBC | Grade III infiltrative Ductal Carcinoma, Triple Negative |
| T13 | 50 | NO | TNBC | Grade III infiltrative Ductal Carcinoma, Triple Negative, Heterogenous but both types TNBC |
| T14 | 56 | NO | TNBC | Grade III infiltrative Ductal Carcinoma, Triple Negative Basal phenotype |
| T15 | 54 | NO | TNBC | Grade III infiltrative Ductal Carcinoma, Triple Negative |
| T16 | 64 | NO | TNBC |  |
| T17 | 66 | NO | TNBC |  |
| T18 | 69 | NO | TNBC | Grade III infiltrative Ductal Carcinoma, Triple Negative Basal phenotype |
| T19 | 65 | NO | TNBC | Grade III infiltrative Ductal Carcinoma, Triple Negative Basal phenotype |
| T20 | 84 | NO | TNBC | Grade III infiltrative Ductal Carcinoma, Triple Negative Basal phenotype |
| T21 | 64 | NO | TNBC | Grade III infiltrative Ductal Carcinoma, Triple Negative Basal phenotype |
